# Modulation of dopamine tone induces frequency shifts in cortico-basal ganglia beta oscillations

**DOI:** 10.1038/s41467-021-27375-5

**Published:** 2021-12-02

**Authors:** L. Iskhakova, P. Rappel, M. Deffains, G. Fonar, O. Marmor, R. Paz, Z. Israel, R. Eitan, H. Bergman

**Affiliations:** 1 Department of Medical Neurobiology, Institute of Medical Research Israel–Canada (IMRIC), The Hebrew University–Hadassah Medical School, Jerusalem, Israel; 2 The Edmond and Lily Safra Center for Brain Sciences, The Hebrew University of Jerusalem, Jerusalem, Israel; 3 Department of Neurobiology, Weizmann Institute of Science, Rehovot, Israel; 4 University of Bordeaux, UMR 5293, IMN, Bordeaux, France; 5 CNRS, UMR 5293, IMN, Bordeaux, France; 6 Department of Neurosurgery, Hadassah University Hospital, Jerusalem, Israel; 7 Jerusalem Mental Health Center, Hebrew University Medical School, Jerusalem, Israel; 8 Department of Psychiatry, Brigham and Women’s Hospital, Harvard Medical School, Boston, MA USA

**Keywords:** Neurodegeneration, Parkinson's disease

## Abstract

Βeta oscillatory activity (human: 13–35 Hz; primate: 8–24 Hz) is pervasive within the cortex and basal ganglia. Studies in Parkinson’s disease patients and animal models suggest that beta-power increases with dopamine depletion. However, the exact relationship between oscillatory power, frequency and dopamine tone remains unclear. We recorded neural activity in the cortex and basal ganglia of healthy non-human primates while acutely and chronically up- and down-modulating dopamine levels. We assessed changes in beta oscillations in patients with Parkinson’s following acute and chronic changes in dopamine tone. Here we show beta oscillation frequency is strongly coupled with dopamine tone in both monkeys and humans. Power, coherence between single-units and local field potentials (LFP), spike-LFP phase-locking, and phase-amplitude coupling are not systematically regulated by dopamine levels. These results demonstrate that beta frequency is a key property of pathological oscillations in cortical and basal ganglia networks.

## Abstract (web summary)

Dopamine tone modulation generates changes in beta oscillation physiology. Here the authors show beta frequency, and not power, coherence, phase-locking, or PAC is monotonically linked to dopamine tone and is likely the key property of pathological oscillations in cortical and basal ganglia networks.

## Introduction

Oscillatory behavior in different frequency bands is common in the cortico-subcortical circuits that loop through the basal ganglia (BG). Activity in the beta-range (human: 13–35 Hz; primate: 8–24 Hz) has been suggested to play a role in several cognitive and motor behaviors. Beta activity is commonly thought to encode the promotion of a “status quo” by maintaining an active process that preserves the current motor directive at the cost of switching to a new one^1^. However, the mechanisms that determine and regulate properties of beta oscillatory activity, like frequency, power and coherence, are still not well defined. There is a significant upregulation of the power (amplitude) of beta activity in the BG of patients with Parkinson’s disease (PD)^2–4^ and PD animal models^5–7^. In line with the “status quo” hypothesis, PD beta activity has been correlated with akinetic symptoms^8^. Thus, traditionally, expression of beta activity in the CBG network has been considered a marker for PD and consequently, many researchers, including our group, focused on studying beta activity amplitude in patients with PD and animal models^5,9^.

The neuropathological hallmark of PD motor symptoms is progressive degeneration of midbrain dopaminergic neurons and their projections to CBG networks. Therefore, a high prevalence of beta oscillations in PD patients and animal models hinted to a potential role for dopamine in generation of beta oscillations. Acute dopamine modulation in rodent animal models has not always resulted in changes in beta properties^10,11^. However, in 6-OHDA rodent models of PD an upregulation of dopamine with apomorphine, a dopamine agonist, resulted in an increase in beta-frequency and a decrease in beta-power^12^. Analysis of PD patients^13^ and MPTP NHP models of PD^5,14^ revealed a high-power beta activity compared to dopamine treated PD patients or NHP normal controls. In the PD circuitry, the high power of beta activity was accompanied by an increase in coherence within and between CBG networks^6,10,12,15,16^. Both were alleviated by dopamine-replacement therapy (DRT) or deep brain stimulation (DBS)^10,12,17–19^. Based on these studies of beta activity in PD patients and animal models, dopamine is thought to play an important role in modulating the power of beta signaling in CBG networks. However, because most of this evidence was derived from dopamine-depleted PD patients and animals models we cannot reliably assume that what we gleaned from these studies provides us with a comprehensive depiction of interactions between dopamine tone and beta oscillation properties, including power, frequency, coherence and single-unit entrainment.

To examine whether dopamine tone is regulating beta activity we recorded single-unit activity (SUA) and local field potential (LFP) in the CBG circuit of two sets of awake, behaving, non-human primates (NHPs). The first pair of healthy NHPs were recorded from after acute dopamine up- and down-modulation using apomorphine (dopamine agonist), amphetamine (dopamine transporter (DAT) inhibitor), and haloperidol (dopamine antagonist). The second pair of NHPs contributed recordings from control and post-MPTP-induced chronic dopamine degeneration. To examine the effects of acute and chronic dopamine modulation in humans we recorded STN LFP in PD patients before and after DRT (Medtronic Inc., Minneapolis, MN, USA). This allowed us to measure changes in beta properties as a function of acute dopamine modulation and progressive dopamine loss that is a hallmark of PD.

In this work we aimed to assemble a comprehensive and nuanced understanding of beta physiology in the CBG circuitry. Our study reveals that beta-frequency, rather than power or any other property, is the key marker of dopamine tone and pathological oscillations in the CBG networks. We further propose that progressive beta-frequency decline, and not a beta-power amplitude, could be used as a more effective indicator of PD progression and as a trigger for adaptive DBS procedures.

## Results

**Fig. 1:**
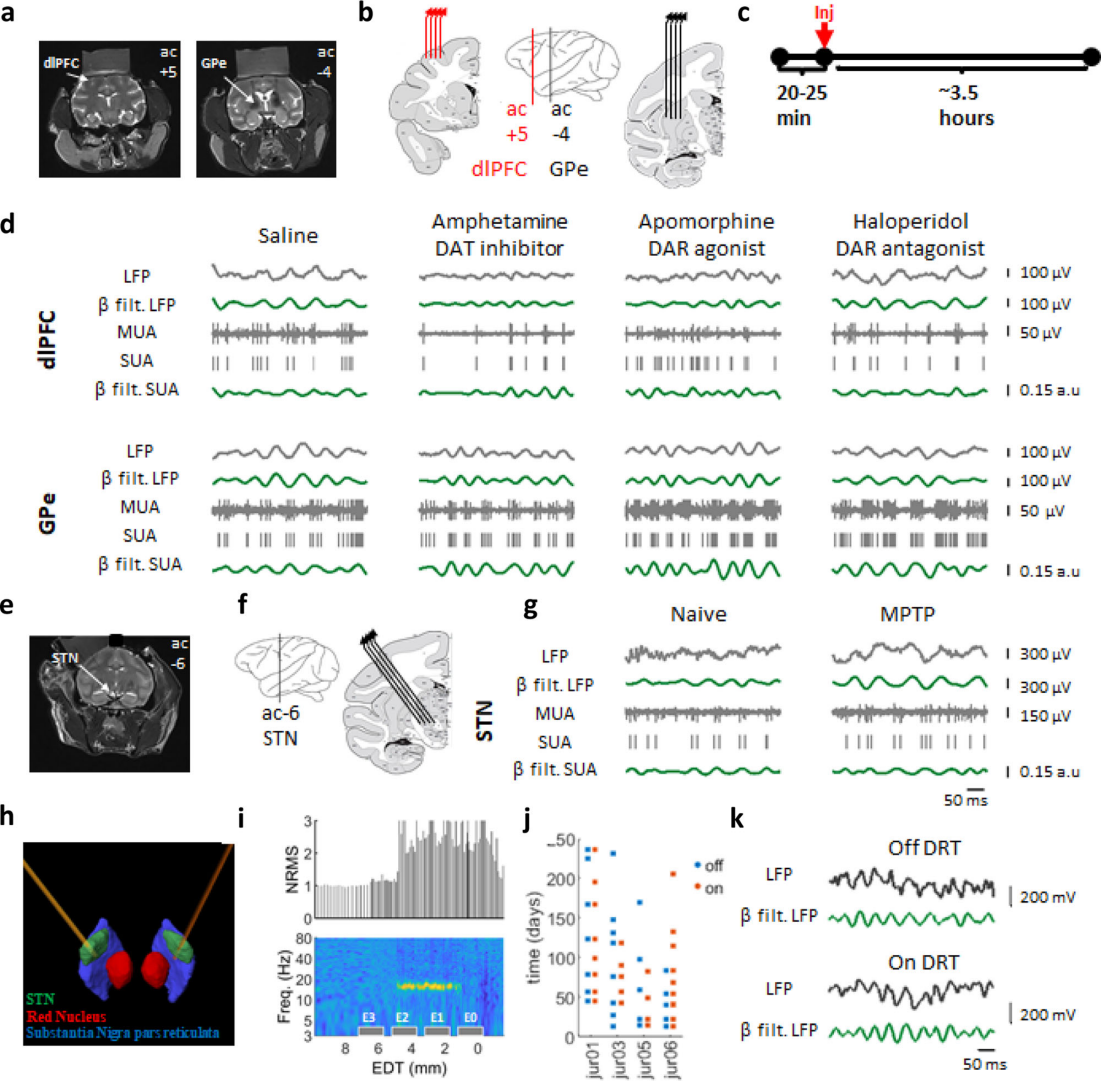
Experiment design. **a** MRI of monkey-G. Coronal images showing recording targets. **b** A scheme of ac+5 and ac−4 coronal planes with four electrodes in each recording target. Middle: coronal plane positions marked on an atlas scheme (Martin and Bowden^22^; http://braininfo.rprc.washington.edu/copyright.aspx). **c** Daily timeline scheme with pre- and post-injection times. **d** 500 ms traces from the dlPFC and GPe under each drug condition. LFP: local field potential, MUA: multi-unit activity, SUA: single-unit activity (top: cortical narrow units, bottom: pallidal units), β filt: beta (8–24 Hz) bandpass filtered. **e**, **f**, **g** Same as **a**, **b**, **d** of monkey K STN. **i** Electrode position marked on a reconstruction of one patient atlas, based on the post-op CT with the pre-op MRI. **i** Electrode contact position relative to STN electrophysiological activity of the same patient as in **h**. *x*-axis indicates estimated distance from target (EDT). The target was set preoperatively close to the imaging based ventro-lateral border of the STN. Top: MUA total power evaluated as normalized root mean square (NRMS). NRMS elevation and decline indicate STN entry and exit, respectively. Bottom: Rectified MUA normalized power spectral density (nPSD, percentage of total power, filtered with a Gaussian window for presentation purposes). Contact positions marked as gray boxes. The STN dorsolateral motor area can be identified according to its pronounced beta activity. **j** Recording schedule of PD patients included in this study. **k** 500 ms traces from the STN of PD patient (same patient as in **h**–**i**) on and off DRT. DAT: dopamine transporter, DAR: dopamine receptor. dlPFC: dorsolateral prefrontal cortex, GPe: globus pallidus pars externa, STN: subthalamic nucleus, DRT: dopamine-replacement therapy.

We examined SUA and LFP recordings collected with multiple micro-electrodes from the dorsolateral prefrontal cortex (dlPFC), and the external segment of the globus pallidus (GPe), the central nucleus of BG circuitry^5^ in awake, behaving monkeys under acute up- and down-modulation of dopamine tone (Fig. 1a–d and Table-S1)^20^. Cortical units were separated into putative-pyramidal cells (wide) and putative-interneurons (narrow) based on the width of the spike shape (Fig. S1)^21^, but see Lemon et.al^22^. We also examined LFP and SUA recordings from the subthalamic nucleus (STN), a BG input nucleus, from monkeys pre- and post-chronic dopamine-denervation via MPTP injection regimen (1-methyl-4-phenyl-1,2,3,6-tetrahydropyridine) (Fig. 1e–g and Table-S1). Additionally, we studied human electrophysiology utilizing multiple post-surgery recordings of bilateral STN LFP from 4 PD patients on and off DRT over a period of 170-250 days (Fig. 1h–k and Table-S2).

To confirm drug effects on NHP behavior, eye physiology was analyzed for changes induced by acute modulation of the dopamine tone. Dopamine level modulation has been shown to significantly affect eye behaviors such as blinking, pupil size, and saccade frequency and amplitude^23–25^. Analysis of eye tracking recordings revealed significant bidirectional effects of dopamine tone on pupil size, saccade frequency and amplitude, closed-eye time, and blink frequency (Fig. S2 and Table-S3). Blinks were classified as shut-eye events 20–500 ms in duration and closed-eye-states included events longer than 500 ms. Dopamine tone downregulation by haloperidol (dopamine receptor (DAR) antagonist) caused decreases in pupil size, saccade frequency and amplitude, and an increase in time spent with eyes closed. Dopamine tone upregulation by amphetamine (DAT blocker) lead to increased pupil size, saccade frequency and amplitude, and a decrease in time spent with eyes closed (Fig. S2). Apomorphine (DAR agonist) effect on behavior was biphasic and mixed. At first (Apo1), the pupil size and blink rate were increased while eye-closed probability decreased. During the Apo2, the trends reversed. Post-injection behavioral effects can be viewed in video recordings (Supplementary Movies 1–4).

### Up- and down-modulation of dopamine tone up- and down-shifts LFP beta-frequency, respectively

**Fig. 2:**
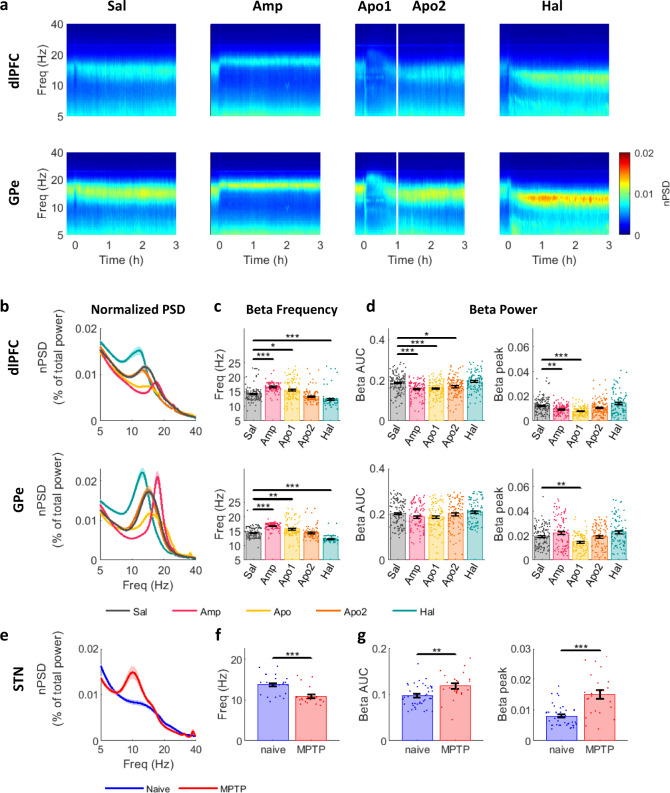
Up- and down-modulation of dopamine tone up- and down-shifts LFP beta-frequency in NHP CBG circuit. **a**–**d** dlPFC and GPe LFP properties after acute dopamine modulation. **a** Average spectrogram of dlPFC and GPe LFP. Time 0 indicates injection time. Color scale indicates nPSD. White line divides the post-apomorphine period into Apo1 and Apo2 phases. **b**–**d** LFP beta properties in dlPFC and GPe **b** Average nPSD during drug influence time (mean ± STE). **c** Frequency of LFP beta peaks (mean ± STE, *N*_dlPFC_ = 527, *N*_GPe_ = 491 LFP sites with detected beta-peak). Beta-frequency was modulated by dopaminergic drugs in the dlPFC (*p* = 9.9e-9^a^, 0.023, 1.6e-8 for Amp, Apo1 and Hal, respectively), and GPe (*p* = 9.9e-9^a^, 0.003, 9.9e-9^a^ for Amp, Apo1 and Hal, respectively). **d** Beta-power evaluated as area under the curve (AUC) of the nPSD, or as nPSD beta peak within 8–24 Hz range (mean ± STE, *N*_dlPFC_ = 611, *N*_GPe_ = 499 LFP sites). Beta AUC was modulated by dopaminergic drugs in the dlPFC (*p* = 2.5e-6, 2.8e-5, 0.017 for Amp, Apo1 and Apo2, respectively). Beta-peak was modulated by dopaminergic drugs in the dlPFC (*p* = 0.003, 1.1e-8, for Amp and Apo1, respectively), and GPe (*p* = 0.003 for Apo1). **c**, **d** Single points indicate individual LFP sites. Outlier values were excluded from the figure, for presentation purposes. Outlier values were defined as data points exceeding 8,5,5 standard deviations distance from the mean for frequency, AUC, and beta-peak, respectively. Drug influence was evaluated by Kruskal–Wallis test followed by post-hoc Tukey test. **e**, **f**, **g** STN LFP properties after chronic MPTP dopamine lesion. Conventions same as **b**, **c**, **d**, respectively. Chronic MPTP modulated beta-frequency (**f**; *N* = 45; *p* = 1.1e-4) and beta power (**g**; *N* = 65; AUC: *p* = 1.1e-4, beta-peak: *p* = 7.3e-7) in the STN. Drug influence was evaluated by two-sided student’s two-sample *t*-test. Test results can be found in Table-S4. Source data are provided as a Source Data file. *p < 0.05, **p < 0.01, ***p < 0.001, ^a^post-hoc *p*-value resolution was limited to 9.9e-9. LFP: local field potential, NHP: non-human primate, CBG: cortico-basal ganglia, dlPFC: dorsolateral prefrontal cortex, GPe: globus pallidus pars externa, STN: subthalamic nucleus, Sal: saline, Amp: amphetamine, Apo1/2: Apomorphine phase 1/2, Hal: haloperidol, (n)PSD: (normalized) power spectrum density.

Acute modulation of dopamine tone shifted the frequency of LFP beta oscillations in both the cortex and GPe (Fig. 2a–c and Table-S4). The population average spectrogram of LFP from dlPFC and GPe revealed a clear shift in frequency and amplitude of beta oscillations in response to modulation of dopamine tone (Fig. 2a). Acute upregulation of dopamine tone by amphetamine shifted the frequency of beta oscillations up and downregulation of dopamine tone by haloperidol shifted beta-frequency down. Apomorphine injection resulted in two distinct phases. At first, similar to amphetamine, frequency of beta oscillations shifted up (Apo1), but about 1 h after injection, and in-line with rapid apomorphine pharmacokinetics^26^, beta-frequency returned to baseline levels (Apo2). Diverging from amphetamine effect, beta-power decreased during Apo1, and as the beta-frequency declined in Apo2, beta-power was reestablished.

Similar to acute treatment, chronic post-MPTP dopamine-denervation resulted in a downward shift in beta-frequency in the STN LFP (Fig. 2e–f; Table-S4) and an increased beta-power (Fig. 2g and Table-S4) and number of oscillatory sites (Fig. S3 and Table-S5).

Effect of dopamine tone modulation on beta-power, as measured by the amplitude of the peak and area under curve (AUC) in beta-range (8–24 Hz), was inconsistent after acute treatment (Fig. 2d and Table-S4). In dlPFC, up-modulation of dopamine tone by amphetamine or apomorphine (Apo1) consistently resulted in decreased beta-power. In the GPe, beta-power decreased during Apo1, but not under amphetamine. However, analysis of the highest 20% of beta-peak values in each drug category showed a significant effect of drug treatment on beta-power within this subsection. Beta-power was higher post-amphetamine and haloperidol and lower post-apomorphine (Apo1), suggesting that in some cases both up-and down-modulation of dopamine tone can induce increases in beta-power.

Since eye behavior was affected by dopamine tone, we further investigated the interaction between LFP beta properties, dopamine tone, and eye-state (Figs. S4, and Fig. S5; Table-S6). This analysis revealed decreases in CBG network beta-frequency during closed-eye-states in all drug conditions except haloperidol. Importantly, drug effects maintained significance when eye-state was included in the analysis. These findings confirmed that the shifts in beta-frequency were not influenced by changes in eye-state, because drug effects on beta-frequency were not compromised by the inclusion of eye-state in the model.

### Up- and down-modulation of dopamine tone up- and down-shifts beta-frequency of spiking activity, respectively

Initial test of single-unit firing rate (FR) confirmed that, as previously reported^9^, acute dopamine up-and down-modulation increases and decreases the FR of single-units in the CBG, respectively (Fig. S6 and Table-S7). Once the single-unit FRs were confirmed to follow previously established patterns, the SUA was examined for expression of beta oscillations.

**Fig. 3:**
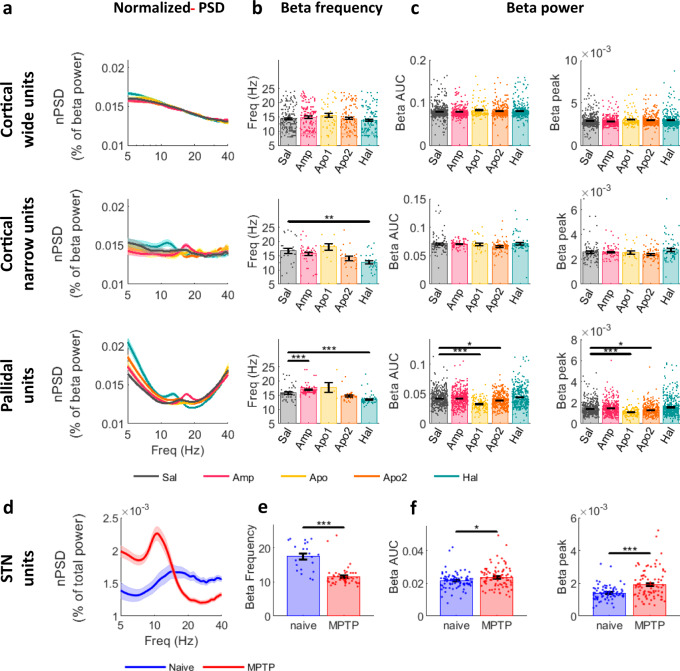
Up- and down-modulation of dopamine tone up- and down-shifts SUA beta-frequency in the cortical narrow, pallidal, and STN units of NHPs. **a**–**c** Single-unit beta properties in cortical wide, narrow, and pallidal units after acute dopamine modulation. **a** Average nPSD during drug influence time (mean ± STE) normalized to extended range beta-power (5–40 Hz) for presentation purposes. **b** Frequency of beta peaks in oscillatory units (mean ± STE, *N*_wide_ = 575, *N*_narrow_ = 124, and *N*_pallidal_ = 227 units). Beta frequency was decreased by haloperidol in narrow (*p* = 0.03) and pallidal (*p* = 2.4e-6) units, and increased by amphetamine in pallidal units (*p* = 0.00086). **c** Beta-power evaluated as area under the curve (AUC) of the nPSD, or as nPSD beta peak within 8–24 Hz frequency range (mean ± STE, *N*_wide_ = 1715, *N*_narrow_ = 318, *N*_pallidal_ = 1627 units). Pallidal unit beta-power was reduced during apomorphine phase 1 (AUC: *p* = 9.9e-9^a^, peak: 1.2e-8) and phase 2 (AUC: *p* = 0.017, peak: 0.04). **b**, **c** Single points indicate individual unit values. Drug influence was evaluated by Kruskal–Wallis test followed by post-hoc Tukey test. **d** nPSD of oscillatory STN units normalized to total power. nPSD of the full dataset can be found in Fig. S7. **e**, **f** STN SUA beta frequency (**e**) and power (**f**) after chronic MPTP dopamine lesion. Conventions same as (**b**, **c**), respectively. Chronic MPTP decreased STN unit beta frequency (**e**; *N* = 71 oscillatory units, *p* = 0.004) and increased beta power (**f**; *N* = 175 units, AUC: *p* = 0.019, beta-peak: *p* = 2.7e-6). Drug influence was evaluated by two-sided student’s two-sample *t*-test. Test results can be found in Table-S7. Source data are provided as a Source Data file. *p < 0.05, **p < 0.01, ***p < 0.001, ^a^post-hoc *p*-value resolution was limited to 9.9e-9. SUA: single-unit activity, NHP: non-human primate, dlPFC: dorsolateral prefrontal cortex, GPe: globus pallidus pars externa, STN: subthalamic nucleus, Sal: saline, Amp: amphetamine, Apo1/2: Apomorphine phase 1/2, Hal: haloperidol, (n)PSD: (normalized) power spectrum density.

In agreement with LFP results, acute dopamine tone modulation up- and down-shifted the frequency of beta oscillations in narrow cortical and pallidal units, but not in the wide cortical units (Fig. 3a, b and Table-S7).

Acute dopamine tone changes also modulated the power of beta oscillations in pallidal units (Fig. 3c and Table-S7), but the relationship between dopamine tone and beta-power was not monotonic. Apomorphine induced a significant decrease in beta-power, while haloperidol and amphetamine showed no statistical difference from control (Fig. 3a, c). However, amphetamine did significantly increase the number of oscillatory cortical narrow and pallidal units (Fig. S7 and Table-S5).

Following previous trends in beta shifts, post-MPTP dopamine-denervated STN SUA also resulted in a downward shift in beta-frequency (Fig. 3d–f and Table-S7), an increased beta-power (Fig. 3f and Table-S7) and number of oscillatory units (Fig. S7 and Table-S5).

LFP and SUA in the saline condition of acutely treated animals were also compared to that of the drug-naïve, to test for injection effect (Figs. S8 and  aS9). In both cortical and pallidal LFP, beta oscillations in the saline condition had a slightly lower frequency relative to that of drug-naïve animals. Pallidal units’ beta-power was a little higher in the saline condition (Table-S8). These effects could be explained as an outcome of minimal damage caused by chronic microelectrode recording and/or mild dopamine depletion during the experiment period. However, no frequency shift was detected in single-unit oscillations between drug-naïve and saline conditions.

### Frequency of beta LFP coherence in the dlPFC and GPe is shifted by acute up- and down-modulations of dopamine tone

**Fig. 4:**
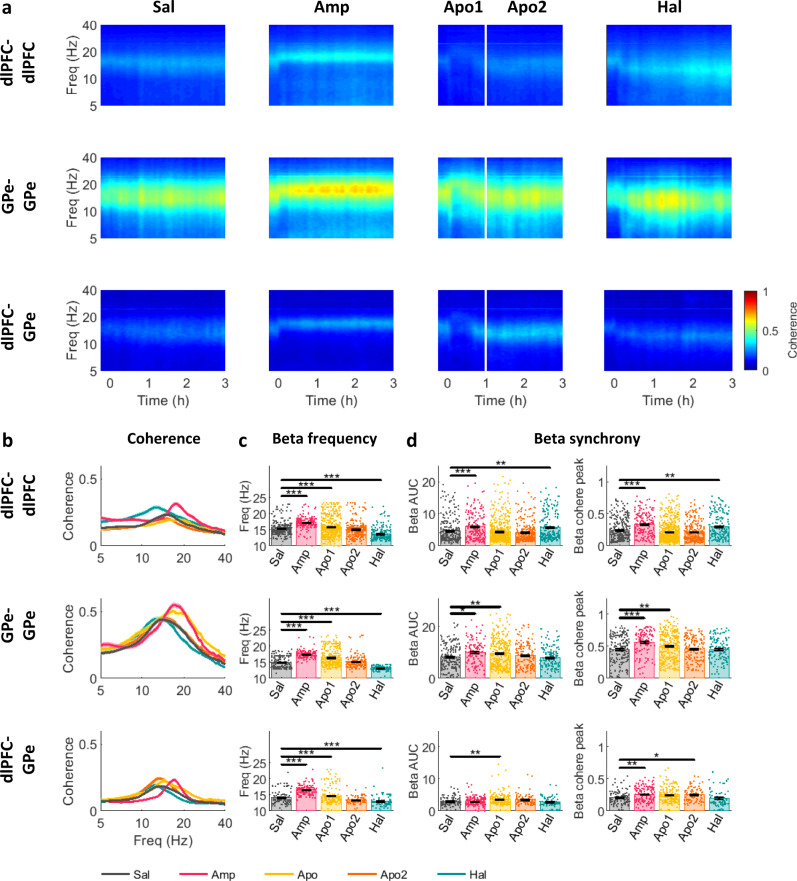
Acute up- and down-modulation of dopamine tone up- and down-shifts LFP beta coherence-frequency in the CBG network of NHPs. **a** Average coherogram of dlPFC-dlPFC, GPe-GPe, and dlPFC-GPe LFP pairs. Time 0 indicates injection time. Color scale indicates magnitude-squared coherence values. White line divides the post-apomorphine period into Apo1 and Apo2 phases. **b**–**d** LFP beta coherence properties in dlPFC-dlPFC, GPe-GPe, and dlPFC-GPe LFP pairs under each drug condition (*N*_dlPFC-dlPFC_ = 1162, *N*_GPe-GPe_ = 45, *N*_dlPFC-GPe_ = 480 LFP pairs). **b** Magnitude-squared coherence (mean ± STE). **c** Frequency of beta coherence peaks (mean ± STE). Beta frequency was increased by amphetamine and apomorphine (phase 1) and reduced by haloperidol in dlPFC-dlPFC (Amp: *p* = 9.9e-9^a^, Apo: *p* = 5.8e-5, Hal: *p* = 9.9e-9^a^) GPe-GPe (Amp: *p* = 9.9e-9^a^, Apo1: *p* = 9.9e-9^a^, Hal: *p* = 9.9e-9^a^) and dlPFC-GPe (Amp: *p* = 9.9e-9^a^, Apo: *p* = 2.9e-5, Hal: *p* = 7.8e-4) LFP pairs. **d** Beta synchrony evaluated as area under the curve (AUC) of the coherence function in 8–24 Hz range, and as coherence peak within 8–24 Hz frequency band (mean ± STE). dlPFC-dlPFC synchrony was increased by amphetamine (AUC: *p* = 5.6e-5, peak: *p* = 6.0e-7) and haloperidol (AUC: *p* = 0.004, peak: *p* = 0.007). GPe-GPe synchrony was increased by amphetamine (AUC: *p* = 0.033, peak: *p* = 4.0e-5) and apomorphine phase 1 (AUC: *p* = 0.001, peak=0.001). dlPFC-GPe synchrony was increased by amphetamine (peak: *p* = 0.004), apomorphine phase 1 (AUC: *p* = 0.003) and phase 2 (peak: *p* = 0.04). **c**, **d** Single points indicate individual LFP pair values. Outlier values were excluded from the figure, for presentation purposes. Outlier results were defined as data points exceeding 8 standard deviations above the mean. Drug influence was evaluated by Kruskal–Wallis test followed by post-hoc Tukey test. Test results can be found in Table-S9. Source data are provided as a Source Data file. *p < 0.05, **p < 0.01, ***p < 0.001, ^a^post-hoc *p*-value resolution was limited to 9.9e-9. LFP: local field potential, CBG: cortico-basal ganglia, NHP: non-human primate, dlPFC: dorsolateral prefrontal cortex, GPe: globus pallidus pars externa, Sal: saline, Amp: amphetamine, Apo1/2: Apomorphine phase 1/2, Hal: haloperidol, (n)PSD: (normalized) power spectrum density, Freq: frequency.

Coherence is used to measure synchrony within and between neural populations in the beta-frequency domain. In agreement with beta trends at single sites (Figs. 2 and 3), up- and downregulation of dopamine tone up- and down-shifted the central frequency of LFP coherence, respectively, in the beta-range within and between dlPFC and GPe (Fig. 4a–c and Table-S9). On the single-unit level, pallidal-pallidal and dlPFC narrow-narrow unit pairs exhibited dopamine tone dependence in coherence beta-frequency (Fig. S10).

Effects of up- and down-modulation of dopamine tone on cortical and pallidal beta coherence were inconsistent (Fig. 4d and Table-S9). In the dlPFC, LFP beta coherence was increased by both dopamine up-modulation via amphetamine and down-modulation by haloperidol (Fig. 4d). However, in the GPe, LFP beta coherence was only increased by dopamine up-modulation by amphetamine or apomorphine (Apo1). LFP beta coherence between dlPFC and GPe was also increased by dopamine up-modulation by amphetamine and apomorphine (Apo1&2). Importantly, apomorphine first induced a reduction in dlPFC-GPe beta coherence (Fig. 4a), similar to its effect on beta-power (Fig. 2a). The significant enhancement seen during Apo1 (Fig. 4d) is probably due to some overlap between Apo1 and Apo2. Analysis of LFP-LFP phase-locking value (PLV) revealed similar shifts in the frequency and degree of beta synchrony (Fig. S11 and Table-S10). Interestingly, while cortical LFP coherence within hemispheres was greater than that between hemispheres, in the GPe, coherence within and between hemispheres was comparable (Fig. S12).

### Acute up- and down-modulation of dopamine tone redirects spike-LFP entrainment to opposing beta-phases

**Fig. 5:**
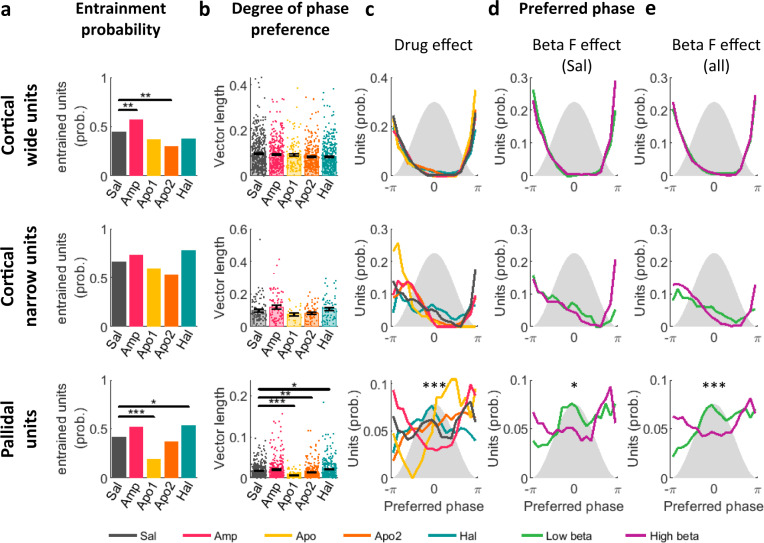
LFP beta-oscillation frequency affects preferred phase of entrained units. Properties of unit-LFP entrainment in the beta-range frequency band of cortical wide, narrow, and pallidal units. **a** Fraction of entrained units out of all units recorded in parallel to oscillatory LFP (*N*_wide_ = 1559, *N*_narrow_ = 303, *N*_pallidal_ = 1374). Dopaminergic drugs modulated entrainment probability of cortical wide (Chi-square test, Amp: *p* = 0.005, Apo2: 0.0017) and pallidal (Apo1: *p* = 1.9e-4, Hal: *p* = 0.045) units. **b** Degree of phase preference, assessed per unit by the vector-length of the spike phase circular average (mean ± STE). Single points indicate individual unit values. Dopaminergic drugs modulated pallidal unit vector-length (*N* = 1374, Kruskal–Wallis test followed by post-hoc Tukey test, Apo1: *p* = 9.9e-9^a^, Apo2: 0.0047, Hal: 0.03). **c**–**e** Preferred phase of entrained units. Gray shadow: LFP beta-cycle, *x*-axis: LFP beta phase. *y*-axis: unit probability to lock to a given phase. **c** Entrained units grouped by drug condition. **d** Oscillatory entrained saline units, grouped by the unit beta frequency. **e** Oscillatory entrained units from all drug conditions, grouped by the unit beta frequency. **d**–**e** Units were segregated into low-beta (green) and high-beta (purple) groups according to the unit beta-frequency using a 15 Hz cutoff. Pallidal unit preferred phase was modulated by drug condition (**c**; circular-median test, *N* = 605, *p* = 9.7e-7), and unit beta-frequency in saline units (**d**; *N* = 143, *p* = 0.049) and for all drug conditions (**e**; *N* = 553, *p* = 0.0006). Test results can be found in Table-S11. Source data are provided as a Source Data file. *p < 0.05, **p < 0.01, ***p < 0.001, ^a^post-hoc *p*-value resolution was limited to 9.9e-9. LFP: local field potential, Sal: saline, Amp: amphetamine, Apo1/2: Apomorphine phase 1/2, Hal: haloperidol, prob: probability.

Dopamine modulation changed the number of units entrained to LFP beta oscillations in cortical wide, narrow and pallidal units (Fig. 5a and Table-S11). Up-modulation of dopamine tone by amphetamine and apomorphine resulted in opposing effects on entrainment probability, whereas amphetamine increased the number of entrained wide cortical units, while apomorphine led to a reduction in entrained cortical and pallidal units. Haloperidol increased the number of entrained pallidal units. Results in narrow cortical units showed similar patterns to pallidal units, though comparisons did not yield a significant difference between control and drug conditions.

The degree of spike-to-LFP entrainment can be assessed by the vector-length of the spike phase circular average. A large vector-length indicates a high tendency of spikes to cluster around a specific phase of the LFP oscillation. Modulation of dopamine tone affected the vector-length in pallidal units. Downregulation of dopamine tone by haloperidol increased the vector-length, and upregulation of dopamine tone by apomorphine (Apo1) decreased the vector-length. During the Apo2 period vector-length recovered and exceeded control values. In cortical wide units our results indicated a significant effect of dopamine modulation on vector-length, but post-hoc comparisons failed to reveal significance. In cortical narrow units, the trends were similar to pallidal units, but with no statistical significance (Fig. 5b and Table-S11).

Next, we focused on the effect of acute drug injections on the preferred phase of entrained units (Fig. 5c left; and Table-S11). These results showed that cortical wide and narrow units maintained their phase-locking to the trough of the beta-cycle throughout the different acute dopamine treatments. In contrast, pallidal units showed a significant modulation of preferred phase by dopamine modulation. Post-hoc comparisons of the circular-median test failed to reveal the origin of this effect, probably due to the low statistical power of the statistical test available for this type of circular data. A visual inspection suggested that in the control condition, preferred phase distribution is bimodal with high probability for units to be entrained to either the peak or trough of the LFP beta-cycle. Dopamine down-modulation shifted pallidal preferred phase to the peak of the beta-cycle, while dopamine up-modulation shifted pallidal preferred phase to either the descending phase (Apo1) or trough (amphetamine) of the beta-cycle.

Given the opposing effect of dopamine up- and downregulation on beta-frequency, we decided to further examine the effect of beta-frequency on the preferred phase of entrained units. First, we examined only units recorded in the control condition that were both oscillatory and entrained to the LFP beta-cycle (Fig. S13). Then these units were segregated into low and high beta-frequency clusters according to their beta-frequency with the cutoff at 15 Hz. In pallidal units, low-frequency beta oscillations increased the entrainment to LFP beta-cycle peak and decreased the entrainment to LFP beta-trough (Fig. 5c and Table-S11). Next, this analysis was repeated for all oscillatory entrained units, regardless of their drug condition, with similar results (Fig. 5c and Table-S11). We also conducted a similar analysis using the LFP beta-frequency as the grouping factor instead of the unit beta-frequency. While this analysis did not reveal any significant effect when applied on saline units, when applied on the entire cohort it showed that low-frequency LFP beta oscillations increased the entrainment of units to LFP beta-peak (Table-S11). As expected, in all cell-type categories, low-beta clusters were heavily occupied by low-dopamine haloperidol recordings and high-beta clusters were mostly composed of high-dopamine amphetamine and Apo1 recordings. Control and Apo2 recordings distributed equally between the two groups.

### Acute up- and down-modulation of dopamine tone up- and down-shifts PAC-preferred beta-frequency in the CBG circuit of NHPs

**Fig. 6:**
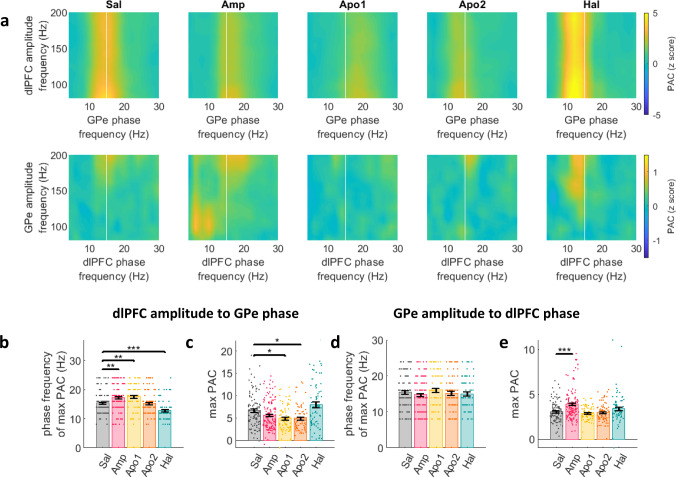
Acute up- and down-modulation of dopamine tone up- and down-shifts frequency and degree of HF-beta PAC. **a** PAC of dlPFC HF amplitude to GPe beta phase (*N* = 459 LFP pairs) and PAC of GPe HF amplitude to dlPFC beta phase (*N* = 457 LFP pairs). *x*-axis: phase frequency, *y*-axis: amplitude frequency. White line marks the 15 Hz phase frequency for reference. **b**–**e** phase frequency (**b**, **d**) and PAC value (**c**, **e**) at maximum PAC (mean ± STE) for dlPFC-to-GPe (**b**, **c**) and GPe-to-dlPFC (**d**, **e**) PAC. Single points indicate electrode pairs. **b**, **c** Dopaminergic drugs modulated phase frequency (**b**; Amp: *p* = 0.003, Apo1: *p* = 0.003, Hal: 9.3e-4) and maximum PAC value (**c**; Apo1: *p* = 0.01, Apo2: *p* = 0.01) of dlPFC-to-GPe PAC (**d**) Dopaminergic drugs did not modulate phase frequency of GPe-to-dlPFC PAC. **e** Amphetamine increased GPe-to-dlPFC maximal PAC value (*p* = 2.6e-5). Drug influence was evaluated by one-way ANOVA test followed by post-hoc Tukey test. Test results can be found in Table-S12. Source data are provided as a Source Data file. *p < 0.05, **p < 0.01,***p < 0.001. HF: high frequency, PAC: phase-amplitude coupling, LFP: local field potential, dlPFC: dorsolateral prefrontal cortex, GPe: globus pallidus pars externa, Sal: saline, Amp: amphetamine, Apo1/2: Apomorphine phase 1/2, Hal: haloperidol.

We examined dlPFC high-frequency (HF) coupling to GPe beta-phase and GPe-HF coupling to the dlPFC beta-phase (Fig. 6a and Table-S12). Similar to other dopamine tone-dependent beta shifts, haloperidol-induced dopamine tone decrease lead to cortical-HF amplitude coupling to a lower GPe beta-frequency, while an increase in dopamine tone lead to coupling at a higher beta-frequency (Fig. 6b). This effect was not present in the GPe-HF amplitude coupling to cortical beta-phase (Fig. 6d). Dopamine-tone upregulation (Apo1) lead to significant decreases in dlPFC-HF to GPe-beta PAC (Fig. 6c). However, in the GPe-HF to dlPFC-beta coupling, amphetamine lead to an increase in PAC (Fig. 6e). Dopamine-tone down-modulation lead to increased PAC trends that did not reach significance (Fig. 6a–c–e).

### In human PD patients, frequency of STN beta oscillations is modulated by DRT and disease progression

**Fig. 7:**
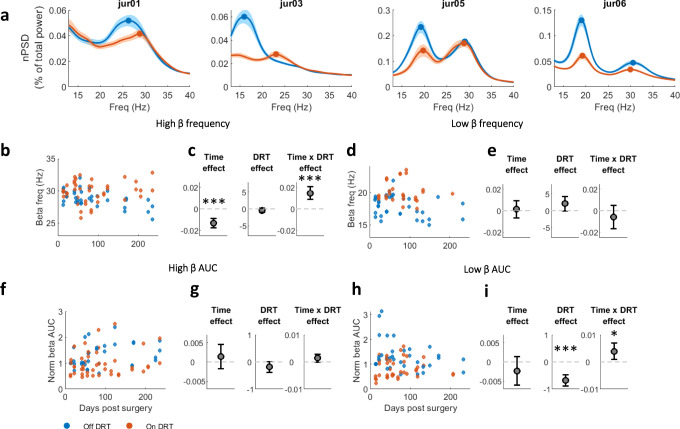
Dopamine modulation shifts STN LFP beta-frequency in patients with PD. **a** normalized PSD (mean ± STE) off (blue) and on (red) dopamine-replacement therapy (DRT) in each patient. **b**, **d** Beta-peak frequency in high (**b**)/low (**d**) beta-domains as function of time post-surgery. Each point represents average per day of beta peak-frequency in one STN on (red) and off (blue) DRT condition. **c**, **e** Time, DRT, and interaction effects on beta-peak frequency as estimated by a mixed linear effect model (MLEM), constructed for *N* = 466 and 418 independent recordings for high-beta frequency (**c**) and low-beta frequency (**e**) models, respectively. Only pairs with significant beta peak were included in the model. Time (*p* = 1.1e-8) and time*DRT interaction (*p* = 6.8e-7) had significant effect on high-beta frequency. Gray circles indicate each factor’s coefficient in the MLEM, and whiskers indicate confidence intervals. Positive and negative coefficients indicate positive and negative linear relation, respectively. In the interaction effect, the coefficient presented is of time given on DRT condition. **f**–**i** Time and DRT effects on beta-power in the high (**f**, **g**) and low (**h**, **i**) beta-domains. Plot conventions same as (**b**–**e**). Beta-power evaluated as baseline-corrected area under the curve (AUC) of normalized PSD in the high (**f**–**g**) and low (**h**–**i**) beta-domains. MLEM was constructed for *N* = 740 and 669 independent recordings for high-beta AUC (**g**) and low-beta AUC (**i**) models, respectively. DRT (*p* = 2.9e-10) and time*DRT interaction (*p* = 0.012) had significant effect on low-beta AUC. The significance of the fixed effects was estimated with ANOVA test (Table-S13). Source data are provided as a Source Data file. *p < 0.05, **p < 0.01, ***p < 0.001.

In humans, beta-range is wider than the 8–24 Hz range in NHP, and is divided into two bands: low-beta (13–23 Hz) and high-beta (23–35 Hz) (Fig. 7a). A PD patient can exhibit beta oscillations in either or both beta-ranges (Fig. 7a). Since PD is a progressive neurodegenerative disease, the time factor probably coincides with the disease-induced chronic degeneration of dopaminergic cells and decrease in dopamine tone. Therefore we expected to see a progressive decline in beta-frequency with time in PD patients.

Assessment of beta oscillation frequency over a period of 250 days post-surgery exposed a consistent decline in frequency of high, but not low, beta oscillations (Table-S13). This decline was more robust in the off-DRT condition relative to the on-DRT condition (Fig. 7b and Fig. S14). DRT did not significantly affect beta-frequency.

The effect of DRT generated similar trends on the power of high- and low-beta domains. However, this decline in power was significant only in the low-beta domain. This can also be seen on the single patient level (Fig. 7a, Fig. S14, Table-S13). DRT affected the time-induced changes in low beta-power suggesting that off-DRT beta-power was reduced over time, while on-DRT beta-power was mildly elevated (Fig. 7i).

### Up- and down-modulation of dopamine tone shifts the frequency of interhemispheric STN beta coherence in PD patients

**Fig. 8:**
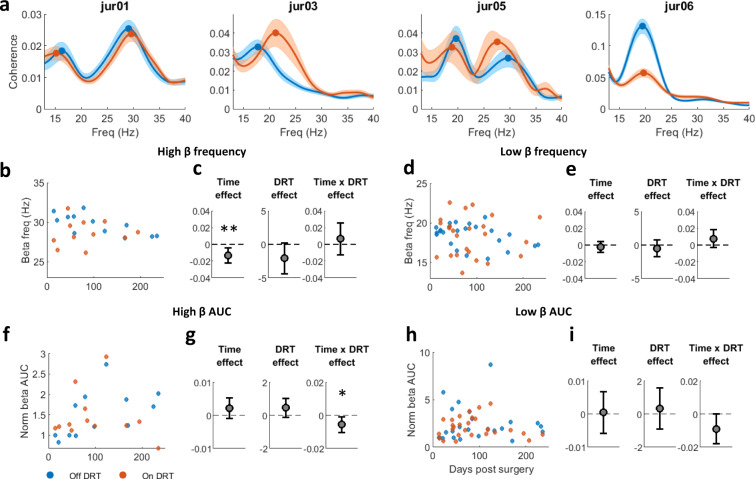
Dopamine modulation shifts beta-frequency of interhemispheric STN LFP coherence in patients with PD. **a** Magnitude-squared coherence (mean ± STE) off (blue) and on (red) dopamine-replacement therapy (DRT) in each patient. **b**, **d** Frequency of beta-coherence peak in the high (**b**)/low (**d**) beta-domains as function of time post-surgery. Each point represents average per day of beta-coherence peak-frequency on (red) and off (blue) DRT. Right: Time, DRT, and interaction effects on beta-coherence peak-frequency estimated by a mixed linear effect model (MLEM), constructed for *N* = 124 and 205 independent interhemispheric electrode pairs for high-beta frequency (**c**) and low-beta frequency (**e**) models, respectively. Only pairs with significant beta peak were included in the model. Time (*p* = 0.005) had significant effect on high-beta coherence peak-frequency. Gray circles indicate each factor’s coefficient in the model, and whiskers indicate confidence intervals. Positive and negative coefficients indicate positive and negative linear relationship, respectively. In the interaction effect, the coefficient presented is of time given on DRT condition. **f**–**i** Time and DRT effects on beta synchrony in the high (**f**, **g**)/low (**h**, **i**) beta-domains. Plot conventions same as **b**–**e**. Beta synchrony is evaluated as baseline-corrected area under the curve (AUC) of the coherence in the high (**f**, **g**) and low (**h**, **i**) beta-domains. MLEM was constructed for *N* = 204 and 480 independent interhemispheric electrode pairs for high-beta AUC (**g**) and low-beta AUC (**i**) models, respectively. Time*DRT interaction had significant effect on high-beta synchrony (*p* = 0.0185). Significance of the fixed effects was estimated with ANOVA test (Table-S13). Source data are provided as a Source Data file. *p < 0.05, **p < 0.01, ***p < 0.001.

In PD patients interhemispheric STN LFP-LFP coherence-frequency was mildly dependent on dopamine tone (Fig. 8a and Table-S13). Chronic dopamine degeneration induced a decrease in frequency of only high-beta coherence (Fig. 8b), while DRT had no effect (Fig. 8c–e). This can also be seen in single patients (Fig. S16). Our results did not reveal any significant medication or time-dependent changes in LFP synchrony (Fig. 8f–i and Table-S13). The effects of time on synchrony in the high and low-beta domains were inconsistent between patients (Fig. S17).

## Discussion

**Fig. 9:**
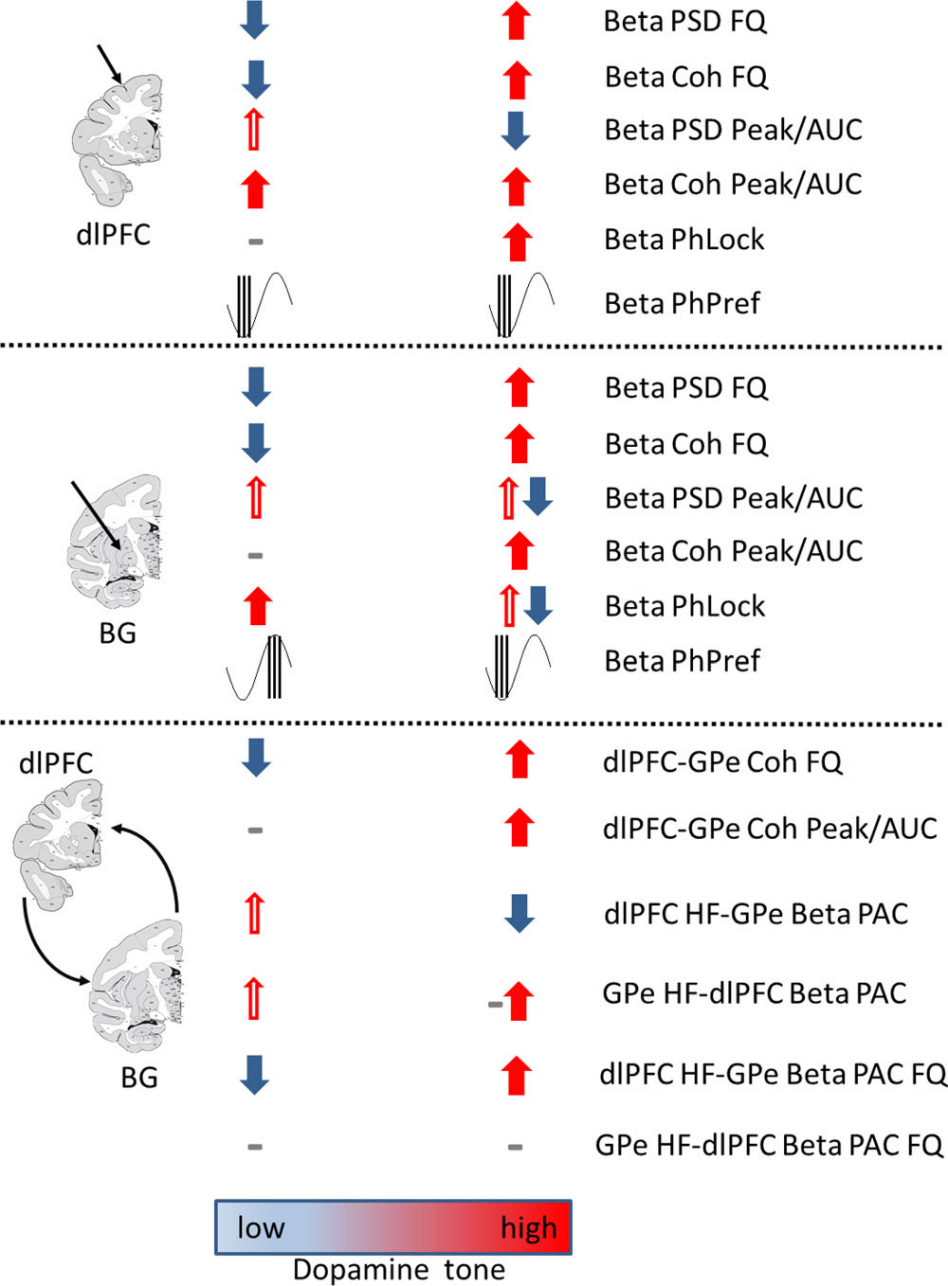
Results summary. Synopsis of LFP and SUA results. Thick arrows indicate statistically significant effects. Thin arrows indicate trends that did not reach statistical significance. A dash indicates no statistical difference. Red arrows indicate increases, blue arrows indicate decreases. Combination of symbols indicates mixed effects. For BG Beta PSD Peak/AUC parameter, thin red arrows represent trends that were significant for the top 20% of all units. dlPFC: dorsolateral prefrontal cortex, BG: basal ganglia, LFP: local field potential, SUA: single-unit activity, PSD: power spectral density, Beta PSD FQ: frequency of beta oscillation. Beta Coh FQ: frequency of beta coherence; Beta PSD Peak/AUC: power of beta oscillation, measured as nPSD beta peak/AUC; Beta Coh Peak/AUC: beta synchrony, measured as magnitude square coherence beta peak/AUC; Beta PhLock: spike to beta LFP phase locking; Beta PhPref: spikes preferred phase in LFP beta-cycle; PAC: phase amplitude coupling, HF: high frequency. Coronal plane positions marked on atlas schemes (Martin and Bowden^22^; http://braininfo.rprc.washington.edu/ copyright.aspx).

Analysis of beta properties in NHPs and PD patients showed that the frequency of beta oscillations, beta coherence, and PAC strongly correlated with acute and chronic changes in dopamine tone. In contrast, effects of dopamine tone on beta-power, beta synchrony, and spike/LFP phase-locking are non-monotonic, inconsistent and less robust (Fig. 9). These results emphasize beta-frequency, and not beta-power as the key property of physiological and pathological beta oscillations in CBG networks.

Previous studies in PD patients and animal models have examined the effects of acute dopamine tone changes by assessing properties of beta oscillations during off and on DRT. These studies suggested that while CBG LFP beta-frequency is not altered by changes in dopamine tone^11,13,27–29^, beta-power is inversely associated to dopamine tone and in a denervated system can be diminished by upregulating dopamine tone via L-Dopa or apomorphine^13,27,29–32^. In our study, acute dopamine up- and down-modulation resulted in clear shifts up and down the beta frequency domain (Figs. 2–3,7). Remarkably, beta-power increases could be generated in the LFP (Fig. 2) and SUA (Fig. 3) by either acute up- or downregulation of dopamine tone. These results suggest that in healthy systems both up- and down-modulations of dopamine tone have the capability to generate high-power beta activity.

Both amphetamine and apomorphine increase dopamine tone, but mechanisms and sites of action are fundamentally different between these two agents. Amphetamine inhibits DAT function, leading to increased activity-dependent dopamine concentration in the synaptic cleft and stimulation of synaptic DARs. Previous studies in rodents and humans measuring beta activity after DAT inhibition, via administration of methylphenidate and cocaine, showed a sharp increase in beta-power^33,34^. Apomorphine, on the other hand, directly stimulates DARs, probably with higher affinity for D2 receptors, in an activity-independent manner and irrespective of their location^35^. Consequently, apomorphine activates not only synaptic and extra-synaptic receptors in the projection nuclei but also autoreceptors that are activated by somatodendritically released dopamine. Once autoreceptors are activated, dopamine dynamics can be altered via inhibition of discharge of dopamine neurons, as well as by reduced dopamine synthesis and release^36–38^. These and other differences between amphetamine and apomorphine could explain the variability in dynamics of beta-power response.

Variability in targets and task difficulty could also explain the differences in beta-properties reported by other groups. Costa et al.^32^ used AMTP, a tyrosine hydroxylase inhibitor, and L-Dopa/carbidopa, a dopamine precursor, to manipulate the dopaminergic system in wild-type and DAT-knockout rodents. Activity in the Costa et al. study was recorded during normal locomotor behavior in a home or novel cage. Previous studies have shown that beta expression is task requirement-dependent, thus low levels of beta-power could be explained by the type of task employed. While beta expression was low during the pre-injection time in the home and novel cages, beta power increased in the post-AMTP period and decreased after L-Dopa/carbidopa administration, similar to our beta power decrease seen in Apo1. However, beta-frequency shifts were not detected, which is expected because little oscillatory activity was expressed in the control and upregulated states. Additionally, their analysis of oscillatory activity used baseline correction, which did not allow for detection of any spontaneous baseline oscillations during the pre-injection period. Conversely, we used the beta activity from our drug-naïve, control (saline), and post-injection recordings in order to build a comprehensive model of beta physiology.

Our results of LFP beta oscillations in PD patients off- and on-DRT (Fig. 7) are in-line with previously published reports confirming a significant medication effect on lowering of beta-power. Here, we have shown that the effect of medication on beta-power is accompanied by its effect on beta-frequency. Hints of this phenomenon can be found in previous reports^13,29^. Acute behavior-dependent changes in STN beta-frequency have been recently reported in PD patients. Whereas beta-frequency was high during walking and shifted down during standing^39^. This evidence suggests that beta activity is not simply associated with a single behavioral component, like status quo or movement idling, but certain types of beta activities, low vs. high frequency, could be linked to distinct behavioral phenotypes.

Previous studies of beta properties after chronic dopamine modulation in human and animal models produced inconsistent results^11,30,40^. In rodents, chronic dopamine-denervation resulted in either no change in frequency or power of beta^30,40^, or a progressive increase in beta-power but no change in beta-frequency^10^. In NHPs, beta-frequency was shown to decline with progressive parkinsonism caused by staggered MPTP administrations^41,42^. This decrease in beta-frequency was accompanied with an increase in power. A human study assessing beta-frequency over a 7 year period in post-DBS surgery PD patients showed no change in beta-frequency and a decrease in beta-power^43^. Our results from NHPs showed an increase in beta power post-MPTP dopamine loss (Fig. 2e–g). Human PD patient results disclosed a consistent and significant time effect post-surgery in high beta-frequency, accompanied with less consistent beta-power changes (Fig. 6b–d and Figs. S14,S15). The difference in data collection methods from human patients could explain the disparity between our results and those previously published^43^. Our method involved multiple recordings in off and on states from each patient over a span of 170 to 250 days post-surgery, creating a chronological trajectory for each patient off and on DRT. While dopamine tone was not directly measured in our patients, persistent dopaminergic degeneration has long been established in PD. These results further support our hypothesis that progressive beta-frequency decline, and not beta-power, could be used as a more effective marker for the progression of PD and as a trigger for adaptive DBS procedures.

Multiple reports concluded that coherence in the beta-range within and between the CBG networks is elevated in a parkinsonian state^6,10,12,15,17^. However, the effect of acute dopamine modulation on beta-range coherence is still debated. Dopamine-depleted rodents showed a decrease in coherence in the beta-range after apomorphine administration^12^, accompanied with an increase in coherence-frequency. Conversely, PD patients off and on DRT showed no change in beta coherence^27^. Here, we showed that acute changes in dopamine tone in NHPs shifted the frequency of LFP coherence in the beta-domain within and between dlPFC and GPe (Fig. 4). These shifts mimicked the shifts in beta-frequency in the power spectrums of single sites/units (Figs. 2–3). In PD patients, presumed chronic dopamine degeneration resulted in consistent decline in coherence-frequency within the high-beta-frequency domain (Fig. 8). However, acute changes in dopamine tone did not significantly affected the degree of synchronization. We therefore suggest that it is not sufficient to rely exclusively on the degree of beta coherence when studying dopamine modulation effect on the CBG circuit. Attention should also be paid to the frequency features of coherence.

Entrainment of spikes to a specific phase of LFP oscillation is a ubiquitous phenomenon in neural circuits. Previously published analysis supports the notion of an increase in single-unit phase-locking to beta LFP with dopamine loss^44^. Our results show that in the GPe, haloperidol-induced entrainment was higher than in control as evidenced in the number of entrained units and degree of entrainment. Low beta-frequency entrained spikes to the peak of LFP beta oscillation while high beta-frequency preferentially locked spikes to the trough of the LFP beta-cycle (Fig. 5d). This defined the relationship between beta-frequency and phase preference, and that in addition to the frequency of beta oscillation, the preferred phase of spike to LFP locking could also be a factor in behavioral outcomes. Previously published reports show that inputs arriving at the optimally excitable phase of beta-cycle of cortical cells lead to responses that are greater in amplitude and have a shorter latency of motor evoked potentials^45^. Additionally, this phase-dependence was found to be more robust during low-beta (16–19 Hz) as opposed to high-beta expression^46^.

Dopamine transmission pathologies lie at the root of many neurological and psychiatric diseases. In dopamine-depleted PD patients and animal models, increase in beta-power has become a hallmark of the disease. However, beta activity was also found in the CBG of subjects with no movement disorders^47,48^. Our study demonstrated that beta-frequency is a more reliable and accurate marker of acute and chronic up- and-downregulation of dopamine tone. Changes in beta-frequency and coherence-frequency were detected after acute and chronic modulation of dopamine tone in NHPs and during chronic progression of PD in human subjects. Whereas previous studies proposed that high beta-power is a unique indicator of dopamine degeneration our data showed that increases in beta-power can be generated via bidirectional shifts in dopamine tone. Notably, dopamine tone modulates cortical HF activity to BG beta PAC and its preferred beta frequency. This HF coupling to low-frequency activity has been suggested to be local spiking coupling to lower frequency oscillations^49^. Finally, acute up- and down-modulation of dopamine tone can lock spikes to opposite phases of beta LFP. Thus opening the possibility that the PAC and spike preferred phase, along with oscillation frequency, contribute to the electrophysiology behind the PD akinetic phenotype.

Further studies that can simultaneously record neuronal activity and detect extracellular dopamine levels could be useful in elaborating the relationship between dopamine tone and beta oscillation frequency. To better understand the unique patterns of oscillatory activity between the normal, hypo- and hyper-dopaminergic states it would be critical to decipher the relationship between beta oscillations and other frequency bands. Our study is limited because we did not selectively target a precise BG nucleus or cortical layer, or modulated specific dopaminergic circuits, or selectively stimulated pre- versus post-synaptic dopaminergic receptors. Utilization of additional technologies could further elucidate the role of specific network components in beta physiology. Laminar probes could be used to record cortical activity in different layers in order to assess layer-specific beta activity^50^. Drug infusion via injectrodes could explore beta physiology after modulation of dopamine tone within specific brain areas^51^. A better understanding of this beta physiology could potentially improve patient care, impact the therapeutic potential of adaptive DBS procedures, and can serve as a foundation and a springboard for further clinically relevant exploration.

## Methods

Further information and requests may be directed to and will be fulfilled by the Lead Contact, Dr. Lily Iskhakova (iskhakova.liliya@weizmann.ac.il).

### Statistics and reproducibility

An experiment comprised of a single recording in either NHP or patient with PD. Each experiment was conducted once.

### NHP experiment: experimental model and subject details

All NHP experimental protocols were conducted in accordance with the National Institutes of Health Guide for the Care and Use of Laboratory Animals (National Research Council, 2011) and with the Hebrew University guidelines for the use and care of laboratory animals in research. The experiments were supervised by the Institutional Animal Care and Use Committee of the Faculty of Medicine, the Hebrew University. The Hebrew University is an Association for Assessment and Accreditation of Laboratory Animal Care internationally accredited institute.

Data was collected from four healthy, young-adult, female vervet monkeys (Chlorocebus aethiops sabaeus), weighing ~4 kg. Data for NHP acute dopamine-modulation experiments was collected from monkeys G (MD-13-13518-4) and D (MD-15-14412-5), and data of NHP chronic dopamine-modulation experiments was collected from monkeys K (MD-14-13997-5), and S (MD-09-13518-5). Monkeys were obtained from the St. Kitts Monkey Farm. The age of monkeys at the time of the experiment was 5–8 years (G: 7-8, D: 5-6, K: 6-7; S: 6-7). Each monkey was trained in a task (see below) ~3 months prior to the implantation surgery and recording. After completion of the recordings, the chambers were removed. Once the monkeys recovered, they were transferred to the Ben Shemen Israeli Primate Sanctuary (www.ipsf.org.il/). One monkey (S) did not recover and was euthanized at the end of the experiment.

### NHP acute dopamine modulation experiment

#### Surgery and post-operative maintenance

A rectangular (34 × 27 mm (inner edge) Cilux recording chamber (AlphaOmega Engineering, Israel) was implanted above a burr hole in the skull under deep anesthesia in aseptic conditions^5^, after the monkeys completed their training in the task (~3 months, 5–6 days a week). A head holder (Crist Instruments, MD) was also implanted during the same surgical procedure. Both the headholder and chamber were manufactured from MRI-compatible Cilux material and were attached to the skull using titanium screws (Crist Instruments, MD) and wires (Fort Wayne metals, IN) embedded in acrylic cement. The central and arcuate sulci of both left and right lobes were located within the boundaries of the chamber, and thus provided bilateral access to the dlPFC and GPe (Fig. 1a, b). Finally, two titanium ground screws (Crist Instruments, MD) were placed in contact with the dura mater and connected to the chamber and head holder using a titanium wire.

About 4 days after the surgery an anatomical MRI scan was performed^5^ to estimate the chamber coordinates of the neuronal targets. Recordings commenced after a postoperative recovery period of 7 to 10 days. Throughout the entire course of the experiment, the chamber was flushed with sterile saline solution every 24–48 h. Chamber cap was sterilized and replaced at the end of each recording day. Once the experiment was completed, another MRI scan was performed to confirm the location of the recording sites and to rule out significant brain shifts.

#### Task and behavior monitoring

NHP subjects were awake and engaged in a behavioral task while head-fixed to the setup in a well lit room. The experiment was conducted in the first part of the day. The analysis of task behavior-related electrophysiology is out of the scope of the current publication. Briefly, a reversal-learning task included multiple blocks with trials consisting visual cues predicting outcomes of different valences. Task included five types of outcomes: palatable and less-palatable food, air puff to the eyes or nose, and neutral outcome (no food or air puff delivered). Predictive cues were shuffled between blocks, so the animals had to learn the new cue-outcome mapping at each block. Anticipatory and event-related licking behavior was recorded via laser lick sensors (Sick Sensor Intelligence).

*Eye tracking*. Eye-behavior data, including pupil size, and eye horizontal and vertical positions were collected using ISCAN system (ISCAN Inc., Woburn, MA U.S.A.). Raw data was sampled at 2750 Hz. Raw data units were arbitrary and relative to the camera, i.e. position units were relative to camera window and not calibrated to the screen. Likewise, pupil size units were proportional to the area on the camera window occupied by the pupil. For blink and saccade detection, raw data was smoothed with 10 millisecond Gaussian moving window.

#### Acute dopamine modulation

The doses for each drug were derived from previously published reports of same drugs administered to NHPs. Our aim was to produce a convincing behavioral change that would last for the duration of the recording using the lowest dose of the drug^52–54^. All injections were made during the last trial of the first block (20-25 m after the beginning of the recording (Fig. 1c). Saline (0.1cc), haloperidol (1 mg/kg) and amphetamine (0.5 mg/kg) were injected intramuscularly (IM) and apomorphine (0.5 mg/kg) was injected subcutaneously (SubQ). All injections were performed in accordance with the manufacturer instructions. For analysis purposes, drug influence time was considered to begin 5 min after drug injection and lasted until the end of the task (>3 h). Apomorphine has a fast dynamic. Its initial effect lasted for ~1 h and then activity returned to baseline. Therefore, we divided post-apomorphine recordings into two phases: Apo1 phase: 5–60 m after the injection followed by the Apo2 phase that lasted until the end of the task.

#### In vivo electrophysiology

During the recording sessions, the monkeys’ heads were immobilized with a head-holder. Local field potentials (LFPs) and single-unit spikes were simultaneously recorded (Fig. 1d) from eight glass-coated tungsten electrodes (impedance 0.45-0.8 MΩ measured at 1000 Hz). Electrodes were arranged in two towers with four electrodes per tower. Each tower was localized to allow targeting and recording from three configurations: bilateral GPe or dlPFC, and unilateral (left or right) GPe/dlPFC. Electrodes were navigated within the brain using the Electrode Positioning System (AlphaOmega Engineering, Israel).

The electrical activity was amplified by 5000, high-pass filtered at 1 Hz using a hardware two-pole Butterworth filter and low-pass filtered at 10 kHz using a hardware three-pole Butterworth filter. Raw data was sampled at 44 kHz by a 16-bit (±1.25 V input range) Analog/Digital (A/D) converter. LFP was low pass filtered at 200 Hz and sampled at 1375 Hz. (AlphaLab SnR Stimulation and Recording System, AlphaOmega Engineering, Israel).

Spiking activity was sorted online using a template-matching algorithm. Up to four different units could be simultaneously isolated from the same electrode. Off-line, the isolation quality of each unit was graded by calculating its isolation score^55^. The isolation score ranged from 0 (i.e., multi-unit activity) to 1 (i.e., perfect isolation). Only units that were recorded for over 1 min and had isolation score ≥0.7 were included in the database. Description of the full dataset can be found in Table-S1.

### NHP chronic dopamine modulation experiment

#### Surgery and post-operative maintenance

In this section, we reanalyzed data that was previously collected and published by our lab^5^. Surgery protocol was similar to that of the acute dopamine-modulation experiment described above, but differed in chamber positioning. Here, a 27*27 (inner edge) recording chamber was tilted at a 45˚ angle laterally in the coronal plane and stereotaxically placed to allow access to the right side of the basal ganglia (BG) nuclei, including the STN.

#### Task and behavior monitoring

Pre-MPTP NHP subjects (monkey K and monkey S) were seated in a well lit room and engaged in a temporal discounting classical conditioning task. Experiments were conducted in the first part of the day to ensure that the monkeys were awake and not drowsy. Monkeys were head-fixed to the setup during the experiment. The analysis of task behavior-related electrophysiology is out of the scope of the current publication. Briefly, the monkey was presented with six different fractal visual cues for 2 s each. Each cue predicted either a food/reward outcome, an air puff/aversive outcome (directed at both eyes) or neither of them/neutral, thus grouping them into three categories. Each cue was followed by the outcome either immediately of after a 6-second delay. Post-MPTP animals did not participate in a task and sat awake and alert during the recording. Licking behavior was recorded using an infra reflection detector (Dr. Bouis Devices, Germany) focused on the mouth area of the animal. Blinking movements were monitored using an infrared digital video camera, which recorded the animal’s face at 50 Hz.

#### Chronic MPTP-induced dopamine-denervation

To induce Parkinsonism, the monkeys were treated with the neurotoxin 1-methyl-4-phenyl-1,2,3,6-tetrahydropyridine (MPTP-hydrochloride, Sigma, Israel). Each animal received five (5) IM injections of 0.4 mg*kg-*injection under Ketamine-induced (10 mg/Kg) sedation. Injections that were administered over a period of 4 days, wherein two injections were given on the first day. Parkinsonian symptoms, including akinesia, rigidity, and tremor, developed gradually during the week of the injections. Akinesia and rigidity preceding the expression of tremor.

We used a modified Benazzouz primate parkinsonism scale^56^ (Benazzouz et al., ^56^) in order to assess the progression of parkinsonism in our primates. After MPTP treatment, both animals experienced rigidity, episodic low-frequency tremor, noticeably flexed posture and a reduction in spontaneous blinking occurrence. Post-MPTP monkeys were fed a high-calorie nutritional supplement (Ensure Plus, Abbott Labs Nutrition, OH) through a nasogastric tube 2–3 times/day because they could not easily feed themselves due to akinesia and tremor. To protect their skin against sores and ulcers, we padded their living quarters with soft mattresses and provided frequent diaper pad changes

Dopamine-replacement therapy (DRT; Dopicar, Teva Pharmaceutical Industries, Israel; 125 mg L-DOPA + 12.5 mg Carbidopa twice a day) was administered to each monkey during feeding after a period of two (monkey K) or three (monkey S) weeks post MPTP injection. Pills were crushed by hand into powder, dissolved in water, and administered orally before food. DRT resulted in significant clinical improvement in both animals and thus further verified the parkinsonism diagnosis. At the end of the experiment, monkey S was euthanized via deep anesthetized with a lethal dose of pentobarbital and then perfused through the heart with saline, followed by a 4% paraformaldehyde fixative solution. The brain was removed and processed for histological confirmation of dopamine depletion. Monkey K was transferred to Ben Shemen Primate Sanctuary.

#### In-vivo electrophysiology

During each experiment, eight glass-coated tungsten microelectrodes (impedance range at 1 kHz: 0.3–0.8 MW) were inserted separately (Electrode Positioning System, AlphaOmega Engineering, Israel) through the brain and toward the STN. This was done while the monkeys’ heads were immobilized with a head-holder. The electrical activity was amplified by 5000, followed by band-pass filtered from 1 to 8000 Hz via a hardware four-pole Butterworth filter. Then the signal was sampled at 25 kHz by a 12-bit (±5 V input range) Analog/Digital (A/D) converter (Multi Channel Processor, AlphaOmega Engineering, Israel). Individual spikes were sorted online using a template-matching algorithm (Alpha Spike Detector, AlphaOmega Engineering, Israel). Each electrode could present up to three different spiking unit types. The timestamp of each detected spike was sampled at 40 kHz. An isolation score program confirmed the quality of each recorded unit^55^. Only units exhibiting an isolation score of 0.6 or above and a stable firing rate for 9 m or more were included in the analysis. Description of the full dataset can be found in Table-S1.

### NHP experiment data analysis

#### Analysis of pupil size, blinks, and saccades

*Blinks and eye-closed event detection*. Blinks were defined as 20–500 millisecond events in which pupil size dropped below the threshold. The threshold was calculated relative to baseline pupil size distribution. We chose the baseline period to be the fragments of time during the inter-trial-intervals during the pre-injection period, to minimize task-related and drug-related biases. A Gaussian function was fitted to the distribution of baseline pupil size data. The threshold for blinks was set to 3 standard deviations below the mean of the Gaussian fit. This Gaussian fit method was selected because it was less affected by extreme samples in comparison to the simple mean and standard deviation calculation and gave more rigorous results. Eye-closed events were similarly detected but with a duration longer than 500 milliseconds.

*Saccade detection*. Since blinks manifested in the position data as sharp downward saccades, blinks were removed from the data along with the 10 millisecond fragment of time before and after a detected blink. Saccades were detected by an algorithm suggested by Engbert and Kliegl^57^. Briefly, saccades were defined as outliers in the two-dimensional velocity plane. The threshold was set to 10 times the velocity standard deviation. Minimum saccade duration was set to 12 milliseconds. For each saccade, saccade amplitude was defined as the distance between most extreme positions during the saccade.

*Pupil size estimation.* As a first step, all pupil-size data points below the blinks/closed-eye threshold (described above) were removed from the data, with limits on duration time. During this step, all blinks, eye-closed events, and sampling errors (such as misdetection of the pupil during recording) were removed. Then, we fitted a Gaussian function to the resulting pupil size distribution during drug time, and extracted its mean. Baseline mean and standard deviation were calculated as described in blink detection. Pupil size, raw data, and estimated mean were normalized using baseline mean and standard deviation.

*Statistical analysis*. To test for significant effect of dopamine modulation on eye behavior (including pupil size, probability of eye-closed state, blink frequency, saccade frequency and saccade amplitude) we preformed one-way ANOVA test followed by post-hoc Tukey test. Outliers, defined as samples that exceeded three SD difference from the mean, were excluded from the analysis. For the assessment of drug effect on eye behavior over time data was segmented into 5 min bins with 2.5 m overlap. For each drug condition, we performed a two-sample *t*-test to compare saline data to drug data at each bin and used Bonferroni correction to control for multiple comparisons.

#### Unit type identification

GPe units were identified as either high-frequency discharge (HFD) neurons or low-frequency discharge (LFD) neurons based on their firing rate and pattern of activity. Units with firing rate above 30 Hz were classified as HFD. Units with firing rate below 30 Hz were manually identified as either HFD’s or low-frequency discharge bursting (LFDB) based on absence or presence of bursts, respectively.

Cortical units were separated into putative-pyramidal cells and putative-interneurons based on the width of the spike shape^21^. The separation criteria was determined according to spike width distribution (Fig. S1). Units with spike width larger than 3 SD over the mean were excluded from further analysis.

#### Spectral analysis

*Power spectrum density (PSD)*. Power spectrum density (PSD) of LFPs and single-units was calculated using the welch method. For LFPs, the signal was first cleaned of high-amplitude artifacts, defined as deviation of over 5 SD from the signal mean. Once such deviation was detected, the surrounding points were also included in the artifact until the LFP resumed value within 3 SD from the mean. Artifacts were replaced with zeros, so as not to influence spectral analysis results. The clean LFP was parsed into 1-min segments with 54 s overlap. For each segment, the PSD in the range of 1–200 Hz was calculated using two-second window with a one second overlap, and frequency resolution of 1/3 Hz. For single units, we created a logical vector of 1000 Hz resolution, with 1 where a spike was detected and 0 elsewhere. The vector was high-pass filtered at 1 Hz using 4-pole Butterworth filter. We then calculated the PSD of spiking activity in the range of 1–200 Hz, using the welch method with a one second window, 500 milliseconds overlap, and frequency resolution of 1/2 Hz. For both LFP and spiking activity, PSD was divided by its sum to get the normalized PSD (nPSD). For LFP, prior to PSD normalization, the PSD portion that was influenced by line noise artifact (50 Hz, 100 Hz, 150 ± 10 Hz) was replaced with interpolated values using shape preserving piecewise cubic interpolation. The wide frequency range and interpolation method were selected to minimize the effect of line noise artifact on the results of beta oscillation properties analysis. Finally, for LFP, PSD was averaged over segments within the drug influence time (see above).

*Identification of oscillatory signals*. To classify either LFP sites or single-units as oscillatory, we examined their nPSDs and looked for a peak in the beta range (8–24 Hz). We required that the prominence of the beta-peak (a measurement of peak size relative to its surrounding) would be sufficiently larger than the noise-level peak-prominence of the nPSD. Noise level was estimated as the median of the peak-to-trough distance in the nPSD. A beta-peak that was two times larger than the noise level was considered sufficiently large, and the LFP/ single-unit was classified as oscillatory (Figs. S3,S7 and Table-S5). Effect of drug injection on percentage of oscillatory sites/units in the post-drug conditions was tested using chi-square test followed by post-hoc comparison with Bonferroni correction for multiple comparisons (Figs. S3,S7 and Table-S5).

*Beta oscillation properties*. Total beta-power was estimated in two ways: (1) As the nPSD’s area under curve (AUC) in the beta range, (2) as the peak value in the beta range. These methods were selected to overcome each other shortcomings. The peak value is a direct estimation of beta-power in its most prominent frequency, but it is affected by the typical 1/f shape of the nPSD. Thus, peak values in lower frequencies tend to be larger. The AUC measures the total power in the beta band and therefore it is a more general estimation that is less affected by the location of the peak. If no peak was found, mean nPSD value in the beta range was utilized instead. Mean nPSD value was preferred to maximum value because the later tends to detect power at the edges of the beta band and reflects the nPSD shape rather than the beta-power per se. We also extracted the beta-peak frequency. If no peak was found the frequency was set to an empty value (not a number; NaN). If more than one peak was found within the beta range, the peak with the highest prominence was chosen for the beta peak and frequency analysis. Signals with beta-AUC larger than 5 SD above the mean were defined as outliers and excluded from further analysis. To test for significant effect of dopamine modulation on beta properties (beta AUC, peak and frequency) we preformed Kruskal–Wallis test, followed by post-hoc Tukey test. Here and in other analyses below Kruskal–Wallis test was chosen as a non-parametric alternative for ANOVA since our data failed to fulfill the test assumptions. In addition, we calculated the Bayes factor to support our statistical inference. In the single-unit dataset, only a fraction of the recorded signals showed robust beta oscillations. Therefore, we repeated the frequency analysis with the subset of oscillatory units (see above). Analysis was conducted with matlab built-in functions. In the Tukey post-hoc test, matlab function (multcompare) did not deliver p values smaller than 10^−10^ due to parameters involving the estimation of the studentized range cumulative distribution function (CDF). We chose to use the built-in parameters since their accuracy is adequate for statistical inference. Beta properties of LFP and single-units in the saline condition were also compared to that of the drug-naïve animals using independent two-sample *t*-test, to test for a possible effect of the injection and recording procedure.

*Beta properties during eye open/closed states.* For each LFP site, clean LFP data (see above) was divided into segments according to eye state (open vs. close, blinks were considered as part of open-eye state). Normalized PSD was calculated for each segment and averaged across segments within each eye-state condition. Beta properties were calculated for the averaged normalized PSD as described above. Eye-state effect was assessed using two-way mixed-design ANOVA with eye state as a within-factor and drug condition as a between-factor, followed by post-hoc pairwise comparison of eye-state effect within each drug with Bonferroni correction for multiple comparisons.

*Coherence and phase-locking value*. For each simultaneously recorded LFP signal or single-unit, traces were segmented into 1 min bins with 30 s overlap, and magnitude-squared coherence was calculated for each bin using the Welch’s overlapped averaged periodogram method with 5 s window, 2.5 s overlap, and frequency resolution of 1/3 Hz in 1–200 Hz range.

Since coherence between two signals is affected by both oscillation amplitude and phase synchrony^58^, we further calculated the phase-locking value (PLV)^58^ between each pair of signals to directly measure the later. PLV was calculated after similar segmentation in the time domain as coherence, and with 1 Hz resolution in the range of 1–80 Hz.

*Beta coherence and phase-locking value (PLV) properties*. As for LFP and single-unit nPSD beta properties, we assessed beta synchronization in two ways: (1) as the coherence AUC in the beta range, and (2) as the peak value in beta range or mean value if no significantly large peak was found. We also extracted the coherence beta-peak frequency. If no peak was found the frequency was set to an empty value (not a number; NaN). We repeated the same analysis for PLV. To test for significant effect of dopamine modulation on beta properties we preformed Kruskal–Wallis test, followed by post-hoc Tukey test.

*Spike-to-LFP entrainment*. If an LFP site was classified as oscillatory, we further analyzed the entrainment of the spike discharge of local units to the LFP oscillations. LFP was band-pass filtered around its central beta-frequency (defined above) ±2 Hz using a four-pole Butterworth filter, and beta-phase was extracted using Hilbert transformation. For each unit, beta-phase at the time of each spike was extracted. The degree of unit-to-LFP entrainment can be measured by the vector-length of the circular average of spike phases. Vector-length values range from 0 to 1. A large vector-length indicates a high tendency of spikes to be clustered around a specific phase of the LFP oscillations. To evaluate dopamine modulation effect on vector-length we preformed Kruskal–Wallis test, followed by post-hoc Tukey test. We further evaluated dopamine modulation effect on entrained unit preferred phase. To classify units as entrained we preformed Rayleigh test for each unit followed by FDR correction for multiple comparison and used a conservative threshold of *p*-value = 0.0001. This threshold was chosen to minimize the effect of false detection on our results. Still the entrainment analysis was more sensitive than the oscillation analysis described above (see Fig. S13). The preferred phase of an entrained unit was defined as the circular mean of its spike-phase distribution. Dopamine modulation effect on phase preference was assessed by circular-median test followed by post-hoc pairwise comparison with Bonferroni correction for multiple comparisons. The circular-median test was chosen over the common Watson-Williams since our data did not fulfill the latter requirements.

We further divided the oscillatory entrained units (see Fig. S13A, second column) into low-beta and high-beta groups according to the unit central beta-frequency (below and above 15 Hz, respectively). We repeated the statistical test to assess the effect of the unit beta-frequency on entrained units’ preferred phase, for units in the saline condition and for all units regardless of their drug condition. We also repeated the same analysis for all entrained units with LFP beta-frequency as the grouping factor instead of unit beta-frequency.

#### Phase-amplitude coupling (PAC)

PAC was calculated for simultaneously recorded dlPFC and GPe LFPs (total: 353 LFP pairs). We sampled 10 m of stable recording. Since some drugs had long raise and decay time we defined for each drug its “most effective” time from which data was sampled. The “most effective” time was 5 m to 3 h post-injection (PI), 5 to 30 m PI, 1 to 3 h PI and 30 m to 3 h PI for amphetamine, apomorphine phase 1, apomorphine phase 2 and haloperidol conditions, respectively. PAC was calculated twice, with GPe as the phase data source and dlPFC as the amplitude data source and vice versa. PAC values were quantified using mean vector-length (MVL) method^59^, which is recommended for long data epochs, recorded at high sampling rates, with a high signal-to-noise ratio^60^. When tested on sample data, MVL was qualitatively equivalent to other methods (modulation index, phase-locking value, see review^60^).

As the first step of PAC analysis, LFPs were bandpass filtered with FIR1 filter (eeglab). For phase data, LFPs were bandpass filtered at theta-beta frequencies (from 4 to 30 Hz in 2 Hz steps) with constant narrow 2 Hz bandwidth. For amplitude data, LFP was bandpass filtered at high frequencies (80–200 Hz in 5 Hz steps) with bandwidth equals to two times the phase frequency^61^. Second, the instantaneous phase and the instantaneous amplitude were extracted from the low- and the high-frequency filtered signal, respectively, after applying the Hilbert transform. A new analytical signal was composed of the angle of the phase-data filtered signal and the amplitude of the amplitude-data filtered signal. Each point in the analytical signal can be presented in the polar (amplitude-phase) plane. In this representation, high phase-amplitude coupling is expressed as a cluster of high-amplitude points (i.e., long vectors) around a specific angle in the polar plane. Therefore, a large mean vector-length (MVL) indicates high degree of PAC. Lastly, a permutation test was performed. The amplitude signal was block-shuffled while the phase signal remained untouched, affecting the synchrony between the signals and preserving their electrophysiological properties. MVL wass calculated for the shuffled data, and the process wass repeated 200 times to create shuffled MVL distribution. The original MVL was normalized using the mean and standard deviation of the shuffled MVL distribution.

For each LFP pair, we extracted the maximal PAC value within the beta-phase frequency range (8–24 Hz), and its phase frequency. Pairs with maximum PAC > 3 SD over the mean were considered outliers and extracted from further analysis. Drug effect on maximum PAC value and phase frequency were assessed using one-way ANOVA, followed by post-hoc Tukey test.

#### Acute and chronic dopamine modulation experiments in patients with PD

The study using data from patients with PD was authorized and supervised by the IRB of Hadassah Medical Center (no. 0403-13-HMO) and the Israel Ministry of Health (no. HT6752). Clinical Trials Registration number: NCT01962194.

#### Patient selection

In this study, four PD patients underwent STN DBS surgery with implantation of the Activa PC + S pacemaker (Medtronic, Inc, Minneapolis, MN, USA)^47^. All patients met accepted inclusion criteria for DBS surgery and signed informed consent that included permission to use, analyze, and publish data. Patients had (i) advanced idiopathic PD; (ii) long-term levodopa use with reduced efficacy, on-off motor fluctuations and increased incidence of medication-induced side effects; (iii) normal cognitive function or mild-moderate cognitive decline as defined by Addenbrooke’s cognitive examination (ACE) > 75 and frontal assessment battery (FAB) > 10. Patient levodopa equivalent dose (LED) was calculated according to Tomilson et al.^62^. Unidentified patient demographic and clinical information is detailed in Table-S14. The study was authorized and supervised by the IRB of Hadassah Medical Center (no. 0403-13-HMO) and the Israel Ministry of Health (no. HT6752). Clinical Trials Registration number: NCT01962194.

#### Intra-operative procedure

STN target coordinates were chosen using Framelink 5 or Cranial software (Medtronic, Minneapolis, USA). In each STN trajectory two electrodes, separated by 2 mm each, were advanced simultaneously in the vertical dimension. The typical distance between vertical recording sites before the entrance to the STN was 200–400 μm. The typical distance between recording sites within the STN was 100 μm. Areas with smaller number of recording sites were postoperatively interpolated to the general number of recording sites. Neurophysiological activity was recorded via polyamide coated tungsten microelectrodes (AlphaOmega Engineering, Nazareth, Israel) with impedance of around of 0.3–0.7 MΩ (measured at 1 KHz). The data were acquired with the NeuroOmega system (AlphaOmega Engineering, Nazareth, Israel).

STN entry and exit were verified intraoperatively by microelectrode recording of multi-unit spiking activity along the trajectory. The final localization of the permanent DBS electrode was determined according to (1) analysis of spontaneous spiking activity, (2) response of spiking activity to passive and active movements and (3) clinical effects of stimulation at the target. In one of our patients (jur05), one contact was malfunctioning, and data from this contact were excluded from the analysis.

The permanent lead implanted during the surgery (model 3389; Medtronic, Inc., Minneapolis, MN) had four contacts. Each contact had a diameter of 1.27 mm and length of 1.5 mm spaced by 0.5 mm intervals. The lead was placed along the dorsal/lateral/anterior-ventral/medial/posterior axis of the STN (Fig. 1e), and contacts were numbered from 0 (ventral) to 3 (dorsal). Generally, contact 1 was placed dorsally to the border between the motor dorsal beta-oscillatory region and the non-motor ventral non-oscillatory region, detected automatically by a hidden Markov model (HMM), for details see^47^ (Fig. 1f).

#### Post-operative clinical assessment and electrophysiological recordings

Patients underwent recordings during 170–400 post-operative days. Recordings from the first week post-surgery were excluded from the dataset to avoid insertion effect. Only recordings from the first 250 days were included in the analysis because recordings after 250^th^ day all came from a single patient (jur01, Fig. S14–S17). Post-operative recordings were acquired in an outpatient setting. Patients had clinical evaluations and recording sessions every 2–4 weeks. During recordings, patients were instructed to sit quietly for the rest-state session, which lasted 3 min. The patients were fully awake and sitting upright during the 3 m recording. The arousal status of the patients was verified by clinical examination. In addition, sessions included recordings during performance of four tasks, which are out of the current paper scope (Provocative Images task, Doubt task, Auditory Go-NoGo task, Emotional Voice Recognition task.^47^).

Recordings took place during an off-medication and on-medication states (Fig. 1j–k, see Table-S2 for number of recording days and sessions). Off-medication recordings took place after overnight withdrawal of DRT. On-medication recordings took place after confirmation of a substantial improvement in the parkinsonian motor clinical symptoms by the patient and the examiner.

LFP activity was recorded from all the bipolar contact pair combinations (0-1, 0-2, 0-3,1-2, 1-3, 2-3) in both hemispheres through the Medtronic PC + S recording setting. Each contact pair recording lasted 30 s. The signal was amplified by 2000, band-passed from 0.5 to 100 Hz, using a 3 pole Butterworth filter, and sampled at 422 Hz by a 10-bit A/D converter (using ± 2 V input range).

#### Spectral analysis

*Signal preprocessing*. LFP signal was filtered between 0.5 and 200 Hz using four-pole Butterworth IIR filter. We observed three types of noise artifacts in our data: ECG artifact, transient high noise artifacts, and line noise artifact. First, we removed ECG artifacts related to the Activa PC + S device. As seen in our data^47^ and reported by Swann et al.^16^, bipolar recordings from the most ventro-medial electrode contact (Contact 0, Fig. 1i) were accompanied by electrocardiogram (ECG) artifact in three out of our four patients. This artifact probably originates from current leakage into the Activa PC + S at the insertion site of the device lead extender over the pectoralis muscle^16^. ECG pulses were identified by their high peak (above 1.2 SD over the mean) and regularity (coefficient of variation (CV) < 0.32). The ECG signal recorded from the bipolar contacts 0-1, 0-2, 0-3 was averaged to create a template for each bipolar recording for each visit. This template was subtracted from every occurrence of the ECG artifact using linear regression to achieve optimal fit with the data^47^. Second, we removed transient high noise artifacts from the data. Transient high noise artifacts were identified according to their large absolute amplitude (>5 SD over the mean). Noise start and end points were defined as return of the absolute amplitude to ≤3 SD distance from the mean. Line noise artifact was removed directly from the PSD (see below).

*Spectral analysis*. PSD was calculated using the welch method with 500 millisecond windows, 100 millisecond overlap, and frequency resolution of 1/2 Hz. PSD was divided by the total power to get the normalized PSD (nPSD). The total power was calculated as PSD sum across frequencies, excluding the PSD portion that was influenced by line noise artifact (46–57 Hz). The wide range of omitted frequencies was selected to minimize the effect of line noise artifact on the results of beta oscillation properties analysis.

*Beta oscillation properties*. In the analysis of PD patients’ LFP beta properties we took a similar approach as described above for the NHP LFP data. However, there are some differences between human and NHP characteristic beta oscillations. Human beta range spans higher frequencies, and it is common to find beta oscillations in two separate beta ranges, low (13–23 Hz) and high (23–35 Hz), in some but not all patients. Therefore, we defined active beta range (low-beta, high-beta or both) manually for each patient according to their mean nPSD. In patient jur03 there was only low-beta activity but its frequency range was exceptionally wide, so for this patient low-beta range was set to 13–25 Hz.

To confirm that low-beta activity was not mistakenly mislabeled alpha activity (8–12 Hz) we also examined the alpha range for any increase in activity expression. This analysis found that only one of the PD patients (jur06) had a peak in the alpha band. This alpha peak was in addition to the double peak beta activity in both the low and high beta sub-bands. This confirmed that our definition of low-beta activity did not overlap with alpha activity.

For each observation (recording from a single bipolar pair at a single session) we calculated beta-power and frequency. Beta-power was defined as the nPSD’s AUC in the beta range. To overcome patient variability in baseline power estimation we performed baseline correction. Beta-power in the first recording day at each bipolar pair was subtracted from all consecutive beta-power values of the same bipolar pair. Beta-frequency was defined as peak value frequency, if a significant peak was found. Significant peaks were defined relative to other peaks in the same nPSD. We considered a beta peak to be significant if its prominence *z*-score was equal to or greater than 0.67, equivalent to 75th percentile in a standard normal distribution.

To assess the contribution of dopamine-replacement therapy (DRT) and disease progress to beta-power and frequency we constructed a linear mixed effect model (MLEM). Our dataset is both nested, i.e., each patient has several recording sites with several observations per site, and unbalanced, i.e., there are different number of recordings per patient. MLEMs can be used on nested and unbalanced datasets and also overcome patient-specific variabilities. MLEM included fixed effects of DRT (on/off) and time from surgery and for the interaction between the DRT and time factors. The model also included random terms for intercept, DRT and time-effects for each patient and each bipolar pair in each hemisphere. We constructed a separate model for beta-frequency in low-beta and high-beta range, which included only significant peaks. We also constructed models for beta-power in low and high beta range, which included all recordings.

*Beta coherence properties*. Magnitude-squared coherence was calculated for each segment using the Welch’s overlapped averaged periodogram method with 250 milliseconds window, 125 milliseconds overlap, and frequency resolution of 1/10 Hz in 1–100 Hz range. Beta analysis was similar to that described above for PSD. Again, beta ranges were manually assigned to each patient according to their average coherence.

### Reporting summary

Further information on research design is available in the Nature Research Reporting Summary linked to this article.

## Supplementary information

Supplementary Information

Description of Additional Supplementary Files

Supplementary Movie 1

Supplementary Movie 2

Supplementary Movie 3

Supplementary Movie 4

Reporting Summary

## Data Availability

The minimum dataset can be accessed in Figshare data repository via 10.6084/m9.figshare.16660858. Source data are provided with this paper as a Source Data file. Source data are provided with this paper.

## Source data

Source Data

## Code availability

Data analyses were performed with custom written scripts in MATLAB (Mathworks, Natick, MA). The code was uploaded to the Figshare data repository and may be accessed via 10.6084/m9.figshare.16660819

## References

[1] Engel AK, Fries P (2010). Beta-band oscillations-signalling the status quo?. Curr. Opin. Neurobiol..

[2] Brown P (2007). Abnormal oscillatory synchronisation in the motor system leads to impaired movement. Curr. Opin. Neurobiol..

[3] Hahn PJ, McIntyre CC (2010). Modeling shifts in the rate and pattern of subthalamopallidal network activity during deep brain stimulation. J. Comput. Neurosci..

[4] Nevado Holgado AJ, Terry JR, Bogacz R (2010). Conditions for the generation of beta oscillations in the subthalamic nucleus-globus pallidus network. J. Neurosci..

[5] Deffains M (2016). Subthalamic, not striatal, activity correlates with basal ganglia downstream activity in normal and parkinsonian monkeys. Elife.

[6] Raz A, Vaadia E, Bergman H (2000). Firing patterns and correlations of spontaneous discharge of pallidal neurons in the normal and the tremulous 1-methyl-4-phenyl-1,2,3,6-tetrahydropyridine vervet model of parkinsonism. J. Neurosci..

[7] Ivica N (2018). Changes in neuronal activity of cortico-basal ganglia-thalamic networks induced by acute dopaminergic manipulations in rats. Eur. J. Neurosci..

[8] Ray NJ (2008). Local field potential beta activity in the subthalamic nucleus of patients with Parkinson’s disease is associated with improvements in bradykinesia after dopamine and deep brain stimulation. Exp. Neurol..

[9] Bergman H, Wichmann T, Karmon B, DeLong MR (1994). The primate subthalamic nucleus. II. Neuronal activity in the MPTP model of Parkinsonism. J. Neurophysiol..

[10] Mallet N (2008). Disrupted dopamine transmission and the emergence of exaggerated beta oscillations in subthalamic nucleus and cerebral cortex. J. Neurosci..

[11] Degos B, Deniau JM, Chavez M, Maurice N (2009). Chronic but not acute dopaminergic transmission interruption promotes a progressive increase in cortical beta frequency synchronization: relationships to vigilance state and akinesia. Cereb. Cortex.

[12] Sharott A (2005). Dopamine depletion increases the power and coherence of β-oscillations in the cerebral cortex and subthalamic nucleus of the awake rat. Eur. J. Neurosci..

[13] Kuhn, A. Pathological synchronisation in the subthalamic nucleus of patients with Parkinson’sdisease relates to both bradykinesia and rigidity.pdf. *Exp. Neurol*. **215**, 380–387 (2009). 10.1016/j.expneurol.2008.11.008 19070616

[14] Connolly AT (2015). Modulations in oscillatory frequency and coupling in globus pallidus with increasing Parkinsonian severity. J. Neurosci..

[15] Nini A, Feingold A, Slovin H, Bergman H (1995). Neurons in the globus pallidus do not show correlated activity in the normal monkey, but phase-locked oscillations appear in the MPTP model of Parkinsonism. J. Neurophysiol..

[16] Swann NC (2018). Chronic multisite brain recordings from a totally implantable bidirectional neural interface: experience in 5 patients with Parkinson’s disease. J. Neurosurg..

[17] Wang DD (2018). Pallidal deep-brain stimulation disrupts pallidal beta oscillations and coherence with primary motor cortex in Parkinson’s disease. J. Neurosci..

[18] Moshel, S. et al. Subthalamic nucleus long-range synchronization—an independent hallmark of human Parkinson’s disease. *Front. Syst. Neurosci*. **7**, 79 (2013). 10.3389/fnsys.2013.00079 PMC3832794 24312018

[19] Yu C, Cassar IR, Sambangi J, Grill WM (2020). Frequency-specific optogenetic deep brain stimulation of subthalamic nucleus improves parkinsonian motor behaviors. J. Neurosci..

[20] Martin, R. & Bowden, D. *Primate brain maps: structure of the macaque brain*. (Elsevier Sci, 2000).

[21] Nowak LG, Azouz R, Sanchez-Vives MV, Gray CM, McCormick DA (2003). Electrophysiological classes of cat primary visual cortical neurons in vivo as revealed by quantitative analyses. J. Neurophysiol..

[22] Lemon, R. N., Baker, S. N. & Kraskov, A. Classification of cortical neurons by spike shape and the identification of pyramidal neurons. *Cereb. Cortex* 1–8 10.1093/cercor/bhab147 (2021). 10.1093/cercor/bhab147 PMC8491674 34117760

[23] Kori A (1995). Eye movements in monkeys with local dopamine depletion in the caudate nucleus. II. Deficits in spontaneous saccades. J. Neurosci..

[24] Spiers ASD, Calne DB (1969). Action of dopamine on the human iris. Br. Med. J..

[25] Taylor JR (1999). Spontaneous blink rates correlate with dopamine levels in the caudate nucleus of MPTP-treated monkeys. Exp. Neurol..

[26] Sam E, Jeanjean AP, Maloteaux JM, Verbeke N (1995). Apomorphine pharmacokinetics in Parkinsonism after intranasal and subcutaneous application. Eur. J. Drug Metab. Pharmacokinet..

[27] Litvak V (2011). Resting oscillatory cortico-subthalamic connectivity in patients with Parkinson’s disease. Brain.

[28] Priori A, Foffani G, Rossi L, Marceglia S (2013). Adaptive deep brain stimulation (aDBS) controlled by local field potential oscillations. Exp. Neurol..

[29] Priori A (2004). Rhythm-specific pharmacological modulation of subthalamic activity in Parkinson’s disease. Exp. Neurol..

[30] Beck MH (2016). Short- and long-term dopamine depletion causes enhanced beta oscillations in the cortico-basal ganglia loop of parkinsonian rats. Exp. Neurol..

[31] Oswal A, Brown P, Litvak V (2013). Synchronized neural oscillations and the pathophysiology of Parkinson’s disease. Curr. Opin. Neurol..

[32] Costa RM (2006). Rapid alterations in corticostriatal ensemble coordination during acute dopamine-dependent motor dysfunction. Neuron.

[33] Herning RI, Jones RT, Hooker WD, Mendelson J, Blackwell L (1985). Cocaine increases EEG beta: a replication and extension of Hans Berger’s historic experiments. Electroencephalogr. Clin. Neurophysiol..

[34] Chemali JJ (2008). Active emergence from propofol general anesthesia is induced by methylphenidate. Bone.

[35] Rice ME, Patel JC (2015). Somatodendritic dopamine release: Recent mechanistic insights. Philos. Trans. R. Soc. B Biol. Sci..

[36] Christiansen J, Squires RF (1974). Antagonistic effects of apomorphine and haloperidol on rat striatal synaptosomal tyrosine hydroxylase. J. Pharm. Pharm..

[37] Farnebo, L. -O. & Hamberger, B. Drug‐induced changes in the release of 3H‐monoamines from field stimulated rat brain slices. *Acta Physiol. Scand*. **84**, 35–44 (1971). 10.1111/j.1748-1716.1971.tb05213.x 4112831

[38] Iversen LL, Rogawski MA, Miller RJ (1976). Comparison of the effects of neuroleptic drugs on pre- and postsynaptic dopaminergic mechanisms in the rat striatum. Mol. Pharmacol..

[39] Canessa A, Palmisano C, Isaias IU, Mazzoni A (2020). Gait-related frequency modulation of beta oscillatory activity in the subthalamic nucleus of parkinsonian patients. Brain Stimul..

[40] Stoffers D (2007). Slowing of oscillatory brain activity is a stable characteristic of Parkinson’s disease without dementia. Brain.

[41] Connolly AT, Jensen AL, Baker KB, Vitek JL, Johnson MD (2015). Classification of pallidal oscillations with increasing parkinsonian severity. J. Neurophysiol..

[42] Wang J (2017). Network-wide oscillations in the parkinsonian state: Alterations in neuronal activities occur in the premotor cortex in parkinsonian nonhuman primates. J. Neurophysiol..

[43] Abosch A (2012). Long-term recordings of local field potentials from implanted deep brain stimulation electrodes. Neurosurgery.

[44] Avila I (2010). Beta frequency synchronization in basal ganglia output during rest and walk in a hemiparkinsonian rat. Exp. Neurol..

[45] Torrecillos F (2020). Motor cortex inputs at the optimum phase of beta cortical oscillations undergo more rapid and less variable corticospinal propagation. J. Neurosci..

[46] Schilberg L (2018). Phase of beta-frequency tACS over primary motor cortex modulates corticospinal excitability. Cortex.

[47] Rappel, P. et al. Subthalamic theta activity: A novel human subcortical biomarker for obsessive compulsive disorder. *Transl. Psychiatry* **8**, 118 (2018). 10.1038/s41398-018-0165-z PMC6006433 29915200

[48] Deffains M, Iskhakova L, Katabi S, Israel Z, Bergman H (2018). Longer β oscillatory episodes reliably identify pathological subthalamic activity in Parkinsonism. Mov. Disord..

[49] Meidahl AC (2019). Synchronised spiking activity underlies phase amplitude coupling in the subthalamic nucleus of Parkinson’s disease patients. Neurobiol. Dis..

[50] Self MW, van Kerkoerle T, Supèr H, Roelfsema PR (2013). Distinct roles of the cortical layers of area V1 in figure-ground segregation. Curr. Biol..

[51] Vanegas, M. I., Hubbard, K. R., Esfandyarpour, R. & Noudoost, B. Microinjectrode system for combined drug infusion and electrophysiology. *J. Vis. Exp*. 10.3791/60365 (2019). 10.3791/60365 PMC7246035 31789311

[52] Castner SA, Goldman-Rakic PS (1999). Long-lasting psychotomimetic consequences of repeated low-dose amphetamine exposure in rhesus monkeys. Neuropsychopharmacology.

[53] Lublin H, Gerlach J, Peacock L (1993). Chronic treatment with the D1 receptor antagonist, SCH 23390, and the D2 receptor antagonist, raclopride, in cebus monkeys withdrawn from previous haloperidol treatment-extrapyramidal syndromes and dopaminergic supersensitivity. Psychopharmacol. (Berl.).

[54] Lewis MH, Gluck JP, Beauchamp AJ, Keresztury MF, Mailman RB (1990). Long-term effects of early social isolation in Macaca mulatta: changes in dopamine receptor function following apomorphine challenge. Brain Res..

[55] Joshua M, Elias S, Levine O, Bergman H (2007). Quantifying the isolation quality of extracellularly recorded action potentials. J. Neurosci. Methods.

[56] Benazzouz A (1995). Riluzole prevents MPTP-induced parkinsonism in the rhesus monkey: a pilot study. Eur. J. Pharmacol..

[57] Engbert R, Kliegl R (2003). Microsaccades uncover the orientation of covert attention. Vis. Res..

[58] Lachaux J-P, Rodriguez E, Martinerie J, Varela FJ (1999). Measuring phase synchrony in brain signals. Hum. Brain Mapp..

[59] Canolty RT (2006). High gamma power is phase-locked to theta oscillations in human neocortex. Science.

[60] Hülsemann MJ, Naumann E, Rasch B (2019). Quantification of phase-amplitude coupling in neuronal oscillations:comparison of phase-locking value, mean vector length, modulation index, and generalized-linear-modeling-cross-frequency-coupling. Front. Neurosci..

[61] Aru J (2015). Untangling cross-frequency coupling in neuroscience. Curr. Opin. Neurobiol..

[62] Tomlinson CL (2010). Systematic review of levodopa dose equivalency reporting in Parkinson’s disease. Mov. Disord..

