# Supplementary material for: Modulation of dopamine tone induces frequency shifts in cortico-basal ganglia beta oscillations
Source: Nat Commun. 2021 Dec 2;12:7026. doi: 10.1038/s41467-021-27375-5 (PMC8640051; doi:10.1038/s41467-021-27375-5)
Supplement: Supplementary file 1 — Supplementary Information [file 41467_2021_27375_MOESM1_ESM.pdf]

Supplementary Figures

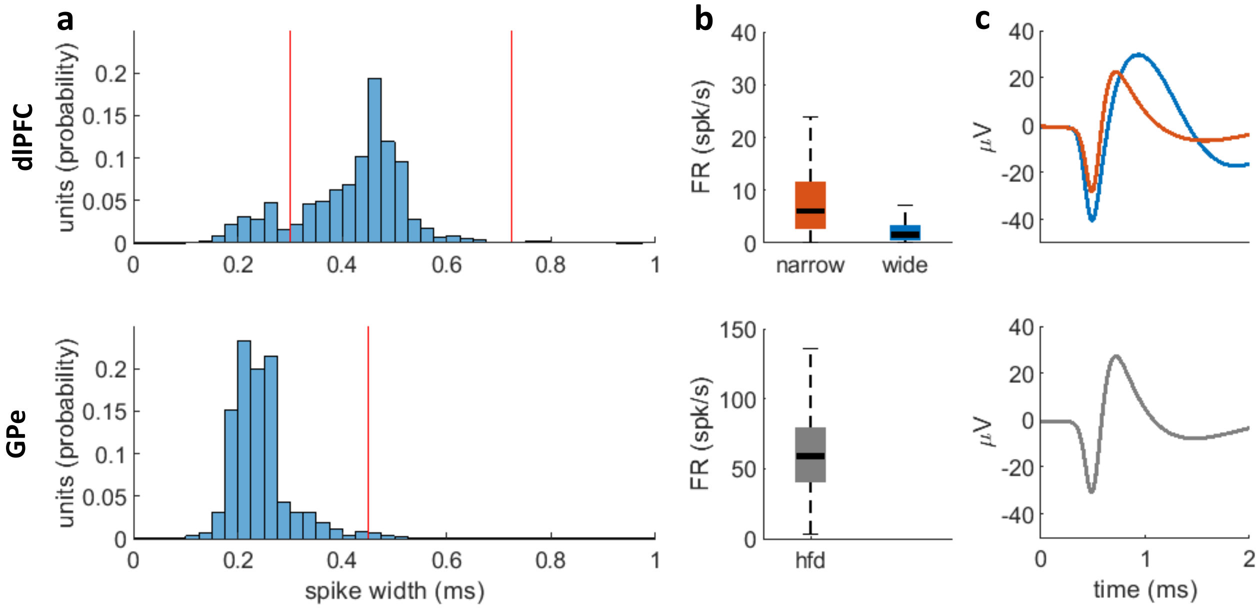

Supplementary Fig.1. Description of cortical and pallidal single unit spike-shape and firing rate properties. dlPFC units and GPe units. (a) Spike width histogram. Cortical wide and narrow units were defined according to their spike width (trough to peak). Units with spike width that exceeded 3 SD over the mean were considered as outliers and excluded from the dataset. (b) Single unit firing rate (N_{narrow}=321, N_{wide}=1736, N_{hfd}=1636 units before outlier exclusion). On each box, the central line indicates the median, and the bottom and top edges of the box indicate the 25th and 75th percentiles, respectively. The whiskers extend to the most extreme data points within 1.5*IQR (interquartile range, equal to the length of the box) distance from the edge of the box. Narrow units had higher firing rate relative to wide units, in-line with their identification as putative interneurons and pyramidal cells, respectively. (c) Average spike shape of cortical wide (blue), narrow (orange), and pallidal high-frequency discharge (HFD) (gray) units. Source data are provided as a Source Data file. dlPFC: dorsolateral prefrontal cortex, GPe: globus pallidus pars externa, FR: firing rate, spk: spikes, s: second, ms: millisecond, uV: microvolt

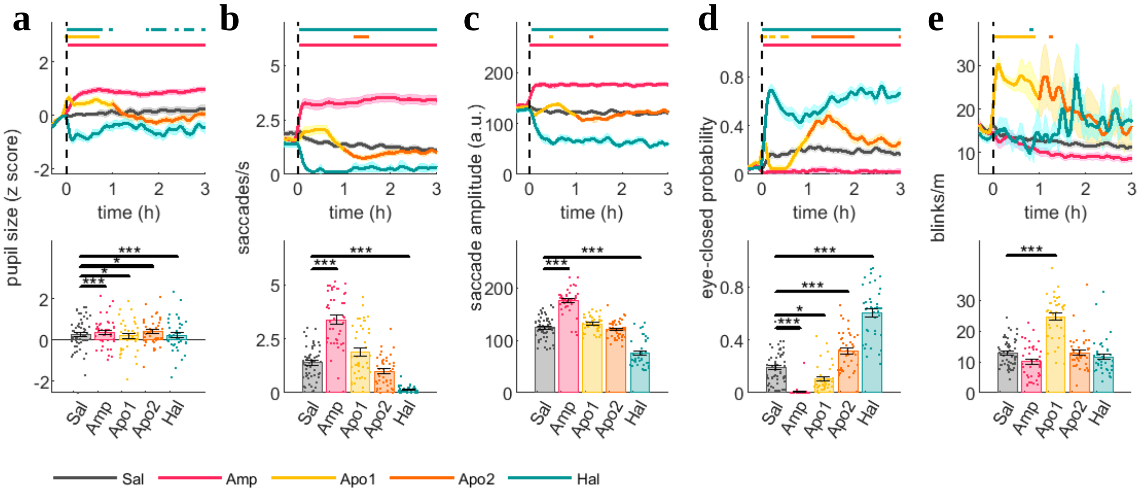

Supplementary Fig.2. Behavioral effect of dopamine tone modulation. Dopamine modulation had an ongoing effect on eye behavior parameters including (a) pupil size, (b) saccade frequency, (c) saccade amplitude, (d) the percentage of time in which the monkey eyes were closed, (e) blink frequency. Upper row: average behavior as a function of time locked to drug injection (dashed line). N=224 days. Shadows indicate standard error of the mean (STE). Straight lines in the upper part of the panel mark time in which behavior post drug injection was significantly different from saline (Two-sample t-test with Bonferroni correction for multiple comparisons. See methods). Lower row: average over time (mean±STE). Each dot marks a single day and bars represent average over days. After outlier exclusion of samples that exceed 3 SD distance from the mean, N=216, 223, 224, 222, and 221 for pupil size, saccade frequency, saccade amplitude, eye-closed probability, and blink frequency, respectively. Drug effects were assessed by one-way ANOVA followed by post-hot Tukey test (Table-S3). Dopamine up-modulation (Amp, Apo1) led to increased pupil size (a; Amp: p=8.3e-8, Apo1: p=0.032), saccade frequency (b; Amp: p=9.9e-9[a]) and amplitude (c; Amp: p=9.9e-9[a]), and blink frequency (E; Apo1: p=9.9e-9[a]), and decreased eye-closure probability (d; Amp: p=1.0e-8, Apo1: p=0.014). Dopamine down-modulation by haloperidol led to an opposite effect in all parameters excluding blink frequency (pupil size: p=1.3e-8, saccade frequency: p=1.0e-8, saccade amplitude: p=9.9e-9[a], eye-closure probability: p=9.9e-9[a]). The behavioral profile during Apo2 was similar to that of dopamine down-modulated post-haloperidol recording (Hal) (pupil size: p=0.018, eye-closure probability: p=1.2e-5).Results are detailed in Table-S3. Source data are provided as a Source Data file. *p<0.05, **p<0.01, ***p<0.001, [a]post-hoc p-value resolution was limited to 9.9e-9. Sal: saline, Amp: amphetamine, Apo1/2: Apomorphine phase 1/2, Hal: haloperidol, h: hours

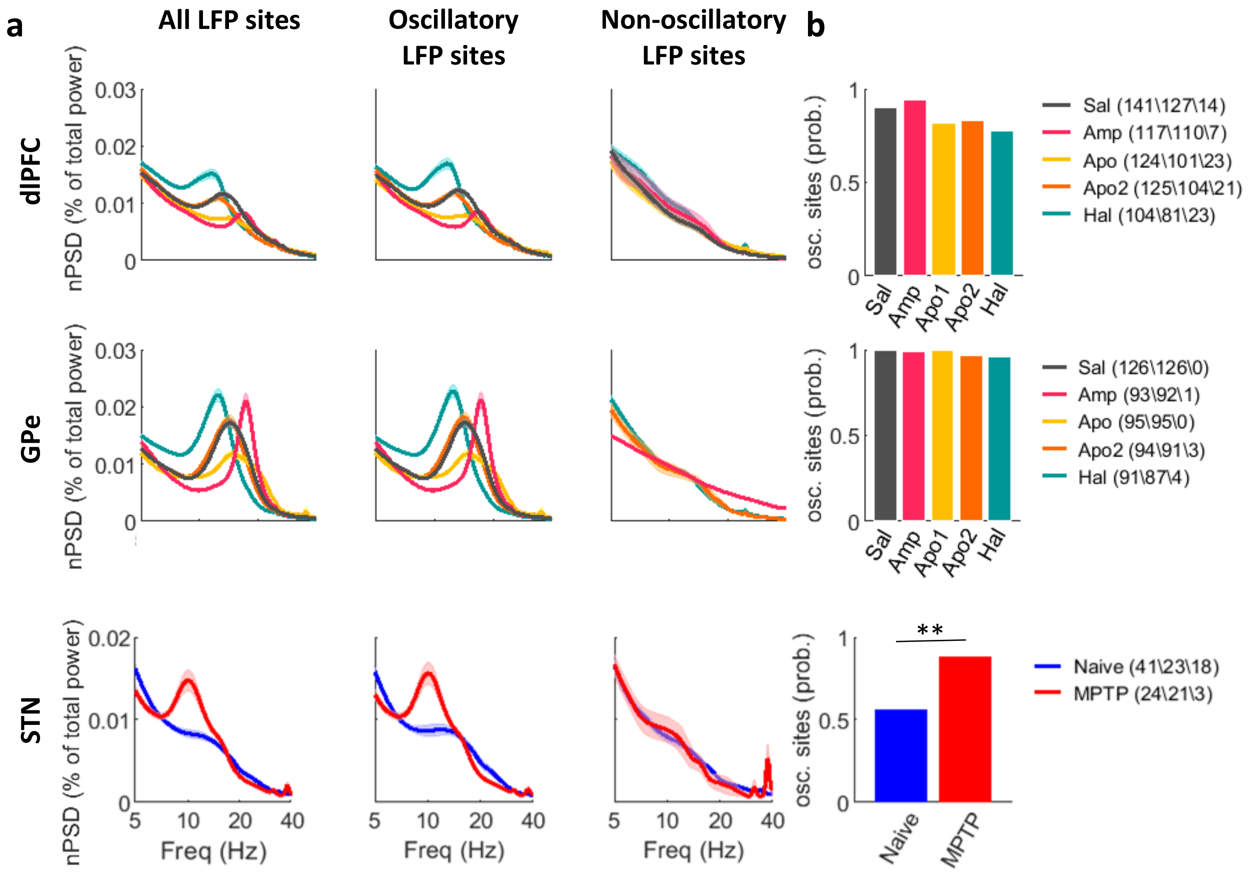

Supplementary Fig.3 Identification of LFP oscillatory sites in NHPs. (a) Average normalized spectrogram (nPSD) of all (left), oscillatory (middle), and non-oscillatory (right) LFP sites in all drug conditions. (b) Fraction of oscillatory sites out of all the recorded sites in each condition. Drug effect was tested with chi-square test followed by pairwise comparisons with Bonferroni correction for multiple comparisons. Top row: dlPFC acute dopamine modulation. Middle row: GPe acute dopamine modulation. Bottom row: STN chronic dopamine modulation. Shadow indicates standard error of the mean. Legend includes counts of all, oscillatory, and non-oscillatory sites, respectively. Chronic MPTP increased the number of oscillatory LFP sites in the STN (chi-square test, N=63 LFP sites, p=0.009). Results are detailed in Supplementary Table 5. Source data are provided as a Source Data file. *p<0.05, **p<0.01,***p<0.001. LFP: local field potential, NHP: non-human primate, dlPFC: dorsolateral prefrontal cortex, GPe: globus pallidus pars externa, STN: subthalamic nucleus, Sal: saline, Amp: amphetamine, Apo1/2: Apomorphine phase 1/2, Hal: haloperidol, (n)PSD: (normalized) power spectrum density, osc: oscillatory, Freq: frequency, prob: probability

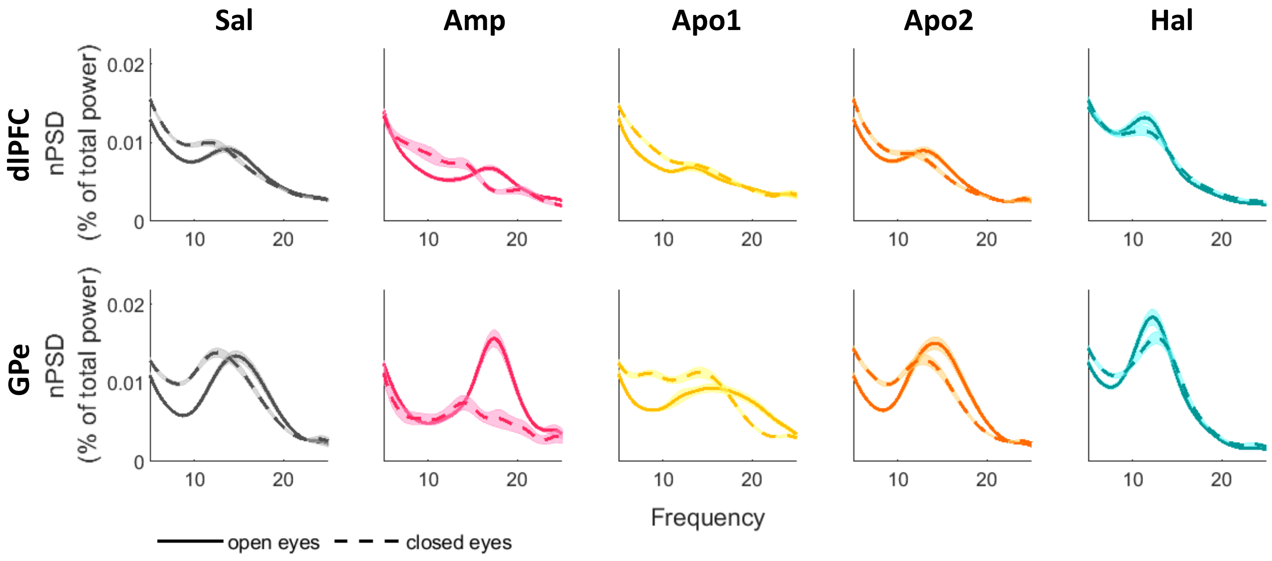

Supplementary Fig.4. Beta activity during eye-open and eye-closed states. Normalized LFP PSD in the dlPFC (top) and GPe (bottom) at times in which the monkey eyes were open (solid line) or closed (dashed line), presented for each drug condition. Shadows mark standard error of the mean (STE). Source data are provided as a Source Data file. nPSD: normalized power spectral density, Sal: saline, Amp: amphetamine, Apo: apomorphine, Hal: haloperidol

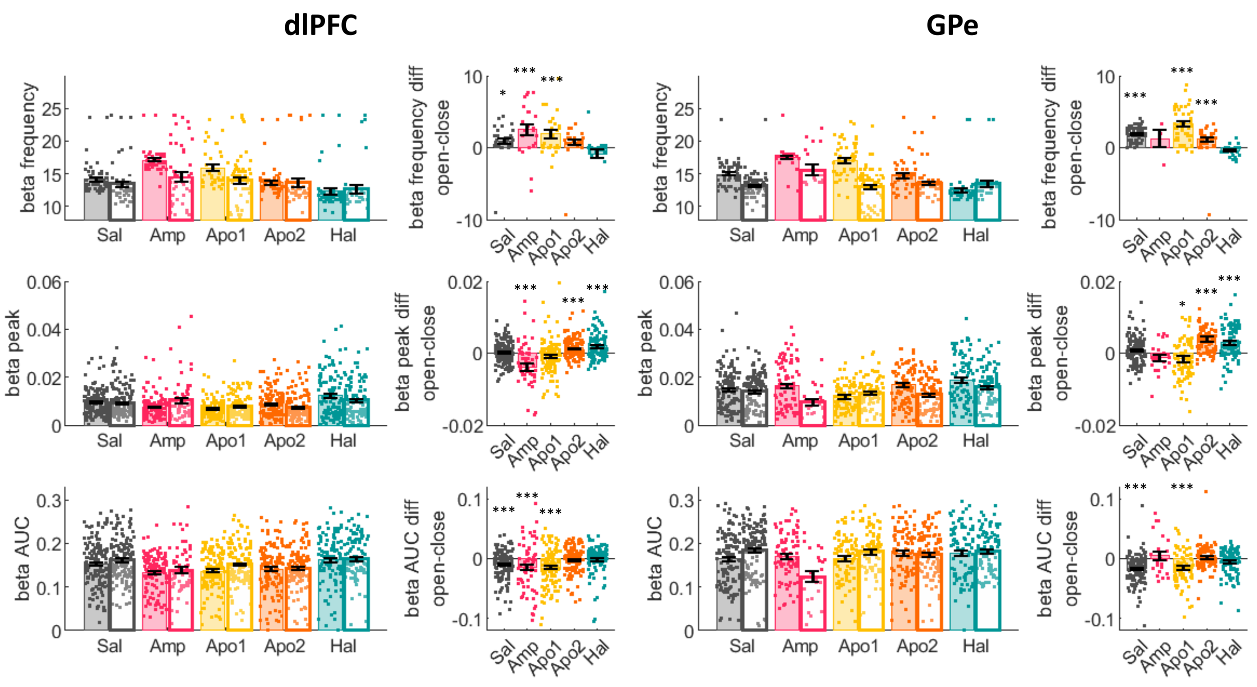

Supplementary Fig.5. Effect of eye state, open vs. closed, on beta oscillation properties. Eye state, open vs. closed, modulated beta frequency, beta peak, and area under curve (AUC) in the dlPFC and GPe. First and third columns: For each day, recording was divided into times at which eyes were open (filled bars) or closed (empty bars), normalized PSD (nPSD) and beta properties were calculated for each state. Each dot represents a single day and bars represent average over days. Whiskers mark the standard error of the mean (STE). Second and fourth columns: Each dot represents the difference in a given parameter between eye-open and eye-closed states for a single LFP site. First row: N_{dlPFC}=142, N_{GPe}=186 LFP sites which were oscillatory in both eye-state conditions. Second and third row: N_{dlPFC}=565, N_{GPe}=414 LFP sites. Bars mark the average difference over days and whiskers mark the STE. Eye-state effect was assessed using a two-way mixed-design ANOVA with eye-state as a within-factor and drug condition as a between-factor, followed by post-hoc pairwise comparison of eye-state effect within each drug with Bonferroni correction for multiple comparisons. Results reveal that within the different drug conditions, eye-closure was associated with a decrease in LFP beta-frequency in the dlPFC (Sal: p=0.035, Amp: p=2.1e-5, Apo1: p=4.0e-4) and GPe (Sal: p=6.2e-16, Apo1: p=7.5e-28, Apo2: p=2.2e-5). Eye-closure effect on beta power depended on drug condition. During saline, Amp and Apo1 conditions eye-closure was associated with increase in beta power in the dlPFC (beta-peak: Amp: p=8.3e-11; beta AUC: Sal: p=2.1e-5, Amp: p=7.8e-5, Apo1: p=1.6e-10) and GPe (beta-peak: Apo1: p=0.02; beta AUC: Sal: p=6.2e-15, Apo1: p=6.5e-9). Conversely, During Apo2 and Hal conditions, eye-closure was associated with decrease in beta peak in the dlPFC (Apo2: p=4.5e-4, Hal: p=1.0e-5) and GPe (Apo2: p=7.4e-11, Hal: p=2.0e-6). Results are detailed in Table-S6. Source data are provided as a Source Data file. *p<0.05, **p<0.01, ***p<0.001. AUC: area under the curve, Sal: saline, Amp: amphetamine, Apo: apomorphine, Hal: haloperidol, diff: difference.

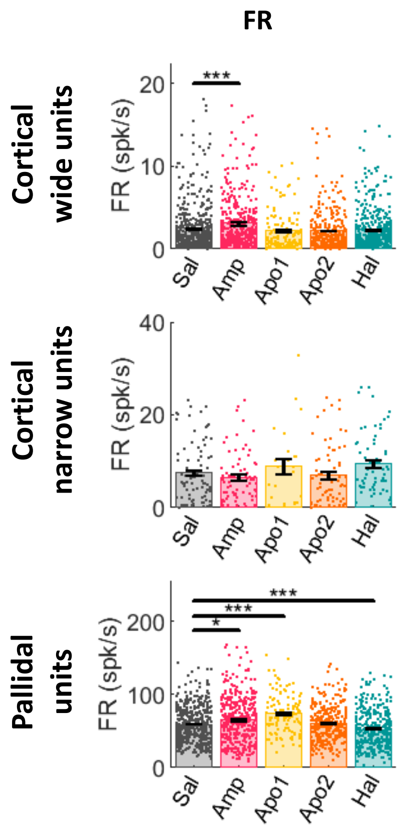

Supplementary Fig.6. Single unit FR. Top: cortical wide units. Amphetamine increased FR. Middle: cortical narrow units. No statistically significant results. Bottom: pallidal units. FR was increased by amphetamine and apomorphine (Apo1), and decreased by haloperidol. Bars indicate average values. Single points indicate individual unit values within the range of mean ± 3 SD. N=1715, 318 and 1627 wide, narrow and pallidal units. Black vertical lines indicate standard error of the mean. Drug influence was evaluated by Kruskal-Wallis test followed by post-hoc Tukey test. FR of cortical wide units was increased by Amp ($p=2.8e-4$). FR of pallidal units was increased by Amp ($p=0.023$) and Apo1 ($p=9.4e-7$) and decreased by Hal ($p=6.0e-4$). Results are detailed in Table-S7. Source data are provided as a Source Data file. *$p<0.05$, **$p<0.01$, ***$p<0.001$ Sal: saline, Amp: amphetamine, Apo1/2: Apomorphine phase 1/2, Hal: haloperidol, FR: firing rate.

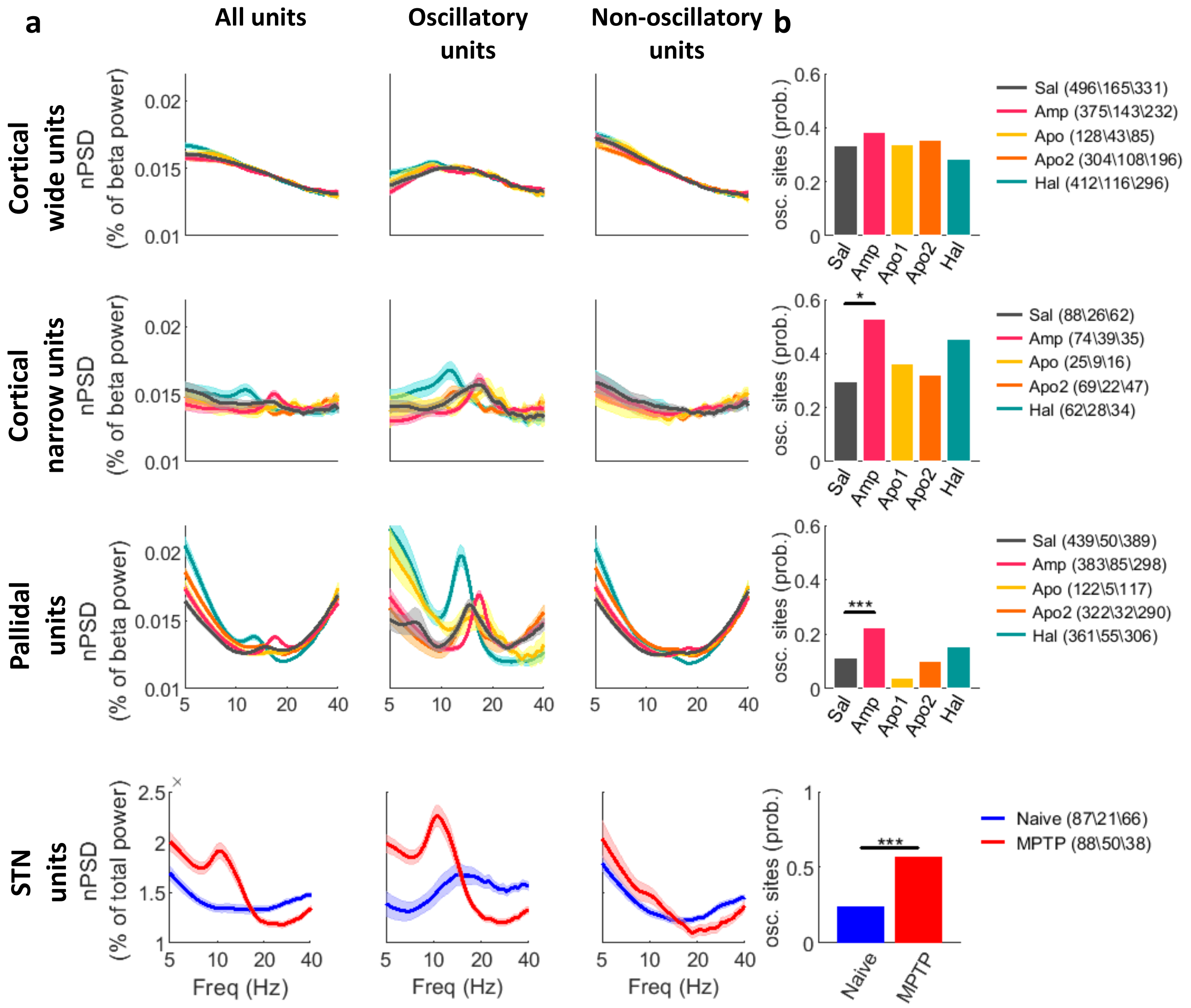

Supplementary Fig.7. Identification of oscillatory units. (a) Average normalized spectrogram (nPSD) of all (left), oscillatory (middle), and non-oscillatory (right) single units for cortical wide (1st row), narrow (2nd row), pallidal (3rd row), and subthalmic (4th row) single units under acute (upper three rows) and chronic (4th row) dopamine modulation. Acute nPSDs normalized to extended range beta-power (5-40Hz) for presentation purposes. Shadow indicates standard error of the mean. (b) Percentage of oscillatory units out of total recorded units in each condition. N_{wide} =1715, N_{narrow}=318, $N_{pallidal}$=1627, N_{STN}=175 units. Drug effects were evaluated using chi-square test followed by pairwise comparisons with Bonferroni correction for multiple comparisons. Amphetamine increased the number of oscillatory units in cortical narrow units (p=0.027) and pallidal units (p=3.0e-4). Chronic MPTP increase the number of oscillatory units in the STN (p=1.1e-5). Legend includes counts of all, oscillatory, and non-oscillatory sites, respectively. Results are detailed in Table-S5. Source data are provided as a Source Data file. *$p<0.05$, **$p<0.01$, ***$p<0.001$. Sal: saline, Amp: amphetamine, Apo1/2: Apomorphine phase 1/2, Hal: haloperidol, nPSD: normalized power spectral density, Freq: frequency, osc: oscillatory, prob: probability.

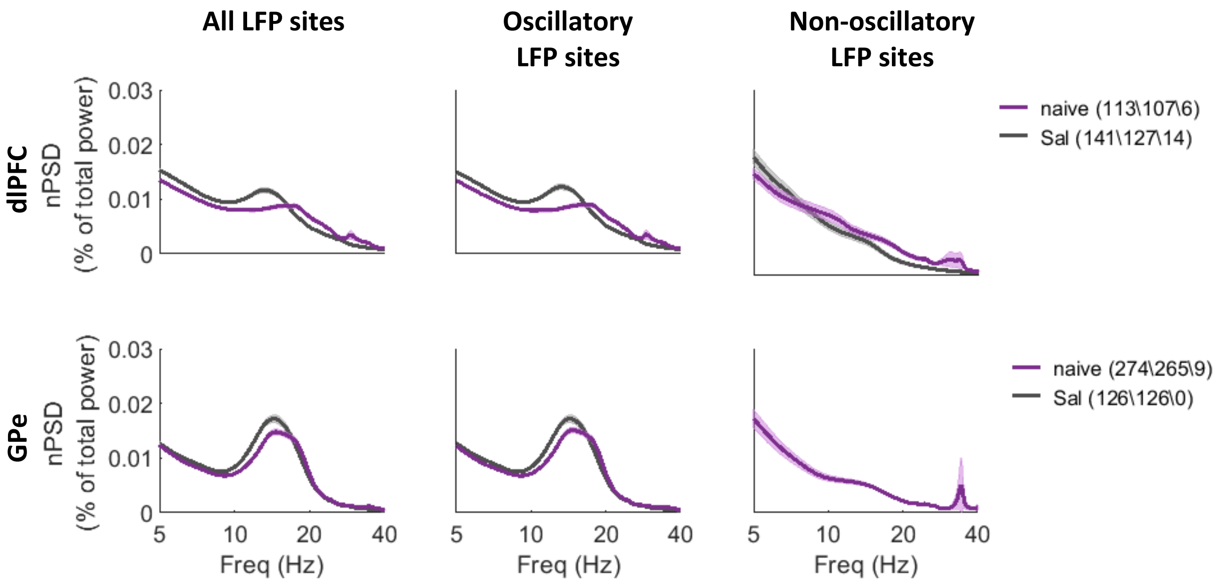

Supplementary Fig.8. Comparisons of LFP beta activity in drug-naïve and control-saline conditions. Average normalized PSD (nPSD) of all, oscillatory, and non-oscillatory LFP sites in drug-naïve and saline conditions within the dlPFC and GPe. Shadow indicates standard error of the mean. Legend includes counts of all, oscillatory, and non-oscillatory LFP sites for each condition, respectively. Results are detailed in Table-S8. Source data are provided as a Source Data file. nPSD: normalized power spectral density, Sal: saline, Freq:frequency.

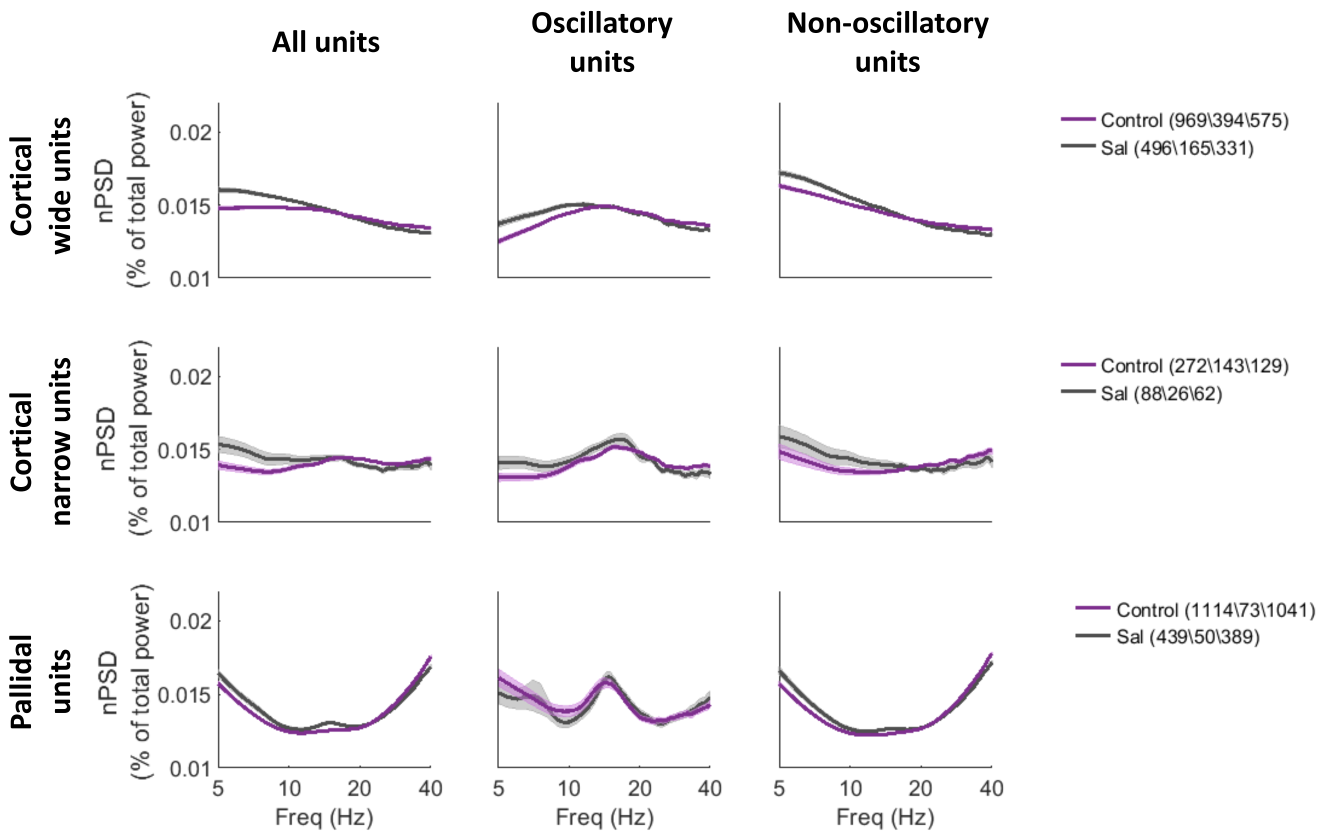

Supplementary Fig.9. Comparisons of SUA beta activity in drug-naïve and control-saline conditions. Average normalized PSD (nPSD) of all, oscillatory, and non-oscillatory single units in drug-naïve and control-saline conditions within the cortical wide units, cortical narrow units, and pallidal units. Shadow indicates standard error of the mean. Legend includes counts of all, oscillatory, and non-oscillatory single units for each condition, respectively. Results are detailed in Table-S8. Source data are provided as a Source Data file. nPSD: normalized power spectral density, Freq: frequency, Sal: saline.

.

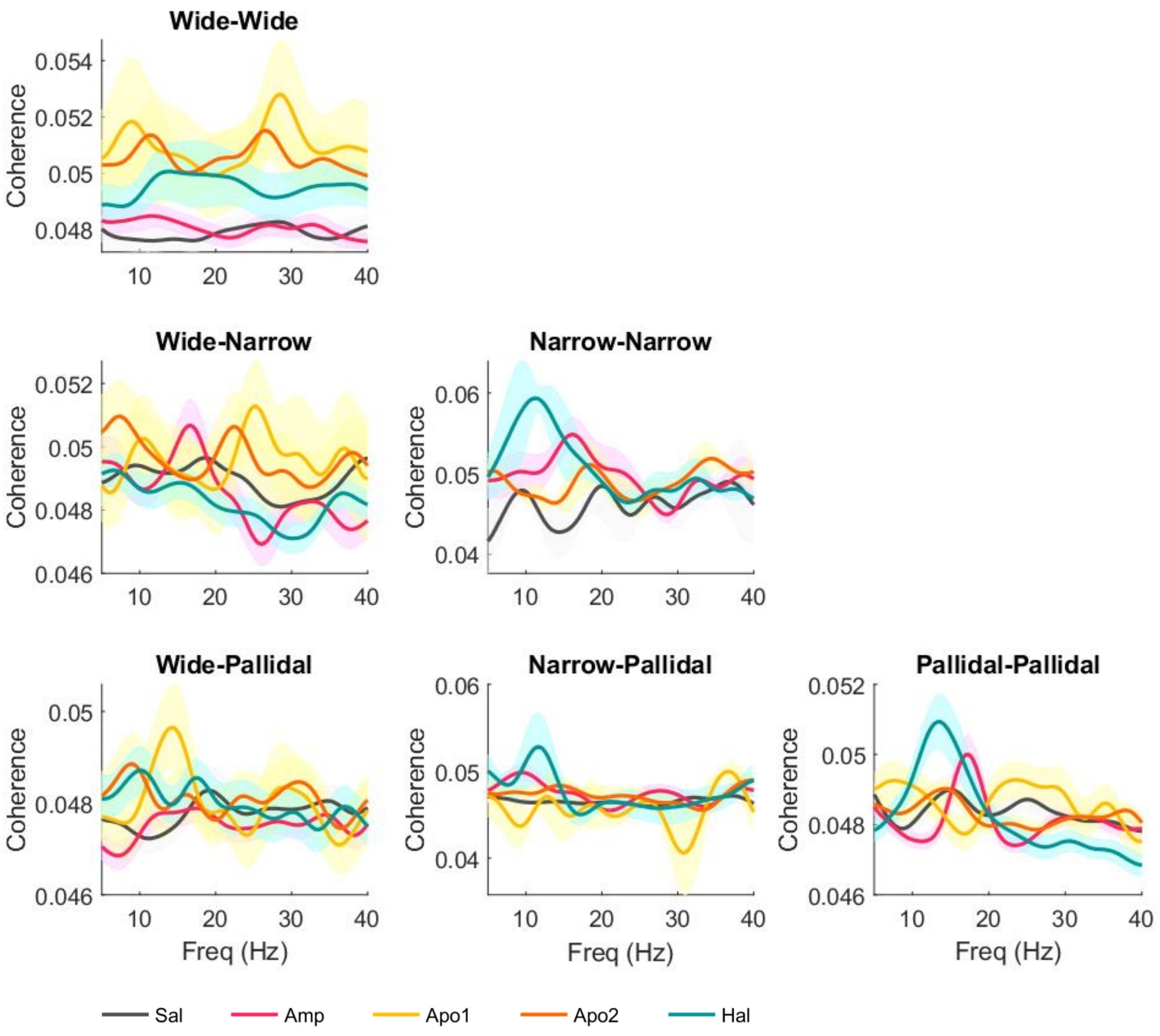

Supplementary Fig.10: Single unit magnitude-squared coherence in cortical narrow and pallidal pairs shows dopamine tone dependent shifts in beta frequency. Shadow indicates standard error of the mean. Only unit pairs that were simultaneously recorded for at least five minutes were included in this analysis. Source data are provided as a Source Data file. Freq: frequency, Sal: saline, Amp: amphetamine, Apo: apomorphine, Hal: haloperidol.

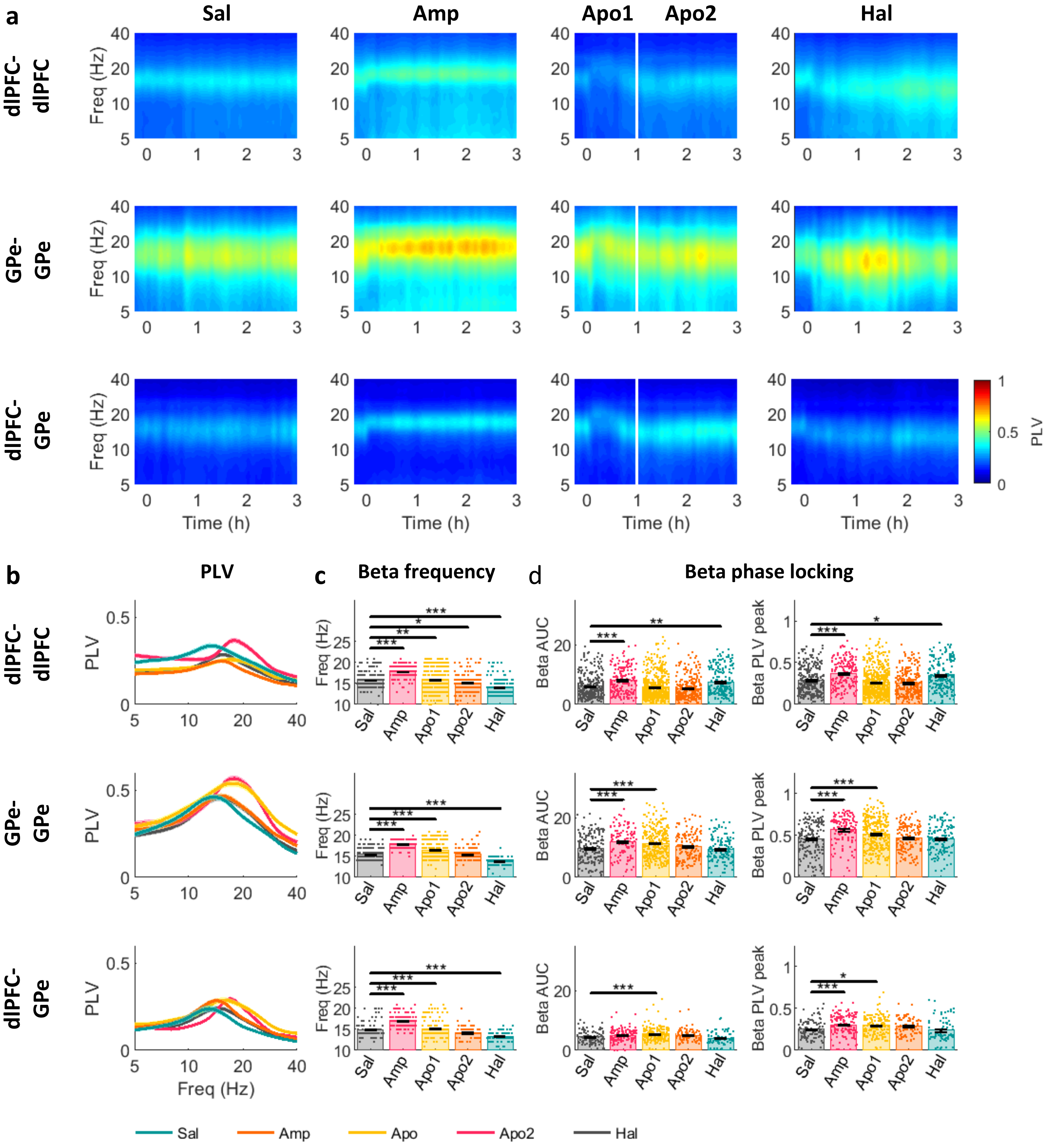

Supplementary Fig.11. **Acute up- and down-modulation of dopamine tone up- and down-shifts the beta frequency of maximal PLV in LFP pairs within the CBG network of NHPs.** (a) Average PLV of dlPFC-dlPFC, GPe-GPe, and dlPFC-GPe LFP pairs. Time 0 indicates injection time. White line divides the post-apomorphine period into Apo1 and Apo2 phases. (b-d) Properties of PLV beta peak in dlPFC-dlPFC, GPe-GPe, and dlPFC-GPe LFP pairs under each drug condition. $N_{dlPFC\text{-}dlPFC}$=1162, $N_{GPe\text{-}GPe}$=745, $N_{dlPFC\text{-}}$

$_{GPe}$=480 LFP pairs. (b) Average PLV during drug influence period (mean±STE) (c) Frequency of PLV peaks (mean±STE). Beta frequency was increased by Amp and Apo1 and reduced by Apo2 and Hal in dlPFC-dlPFC (Amp: p=9.9e-9[a], Apo1: p=0.007, Apo2: p=0.019, Hal: p=9.9e-9[a]) GPe-GPe (Amp: p=9.9e-9[a], Apo1: p=9.9e-9[a], Hal: p=9.9e-9[a]) and dlPFC-GPe (Amp: p=9.9e-9[a], Apo1: p=2.9e-4, Hal: p=1.8e-6) LFP pairs. (d) Overall beta phase locking in the beta range was evaluated as area under the PLV curve (AUC) in 8-24Hz range, and as the PLV peak within 8-24Hz frequency band (mean±STE). dlPFC-dlPFC PLV was increased by Amp (AUC: p=5.1e-6, peak: p=2.1e-6) and Hal (AUC: p=0.010, peak: p=0.012). GPe-GPe PLV was increased by Amp (AUC: p=7.8e-4, peak: p=5.3e-6) and Apo1 (AUC: p=1.9e-5, peak: p=2.4e-4). dlPFC-GPe PLV was increased by Amp (peak: p=7.6e-4), and Apo1 (AUC: p=1.7e-4, peak: p=0.014). (c-d) Single points indicate individual LFP pairs. Outlier values were excluded from the figure, for presentation purposes. Outlier values were defined as data points exceeding 8 standard deviations above the mean. Drug influence was evaluated by Kruskal-Wallis test followed by post-hoc Tukey test. Test Results are detailed in Table-S10. Source data are provided as a Source Data file. *p<0.05, **p<0.01, ***p<0.001, [a]post-hoc p-value resolution was limited to 9.9e-9. PLV: phase locking value, LFP: local field potential, CBG: cortico-basal ganglia, NHP: non-human primate, dlPFC: dorsolateral prefrontal cortex, GPe: globus pallidus pars externa, Sal: saline, Amp: amphetamine, Apo1/2: Apomorphine phase 1/2, Hal: haloperidol

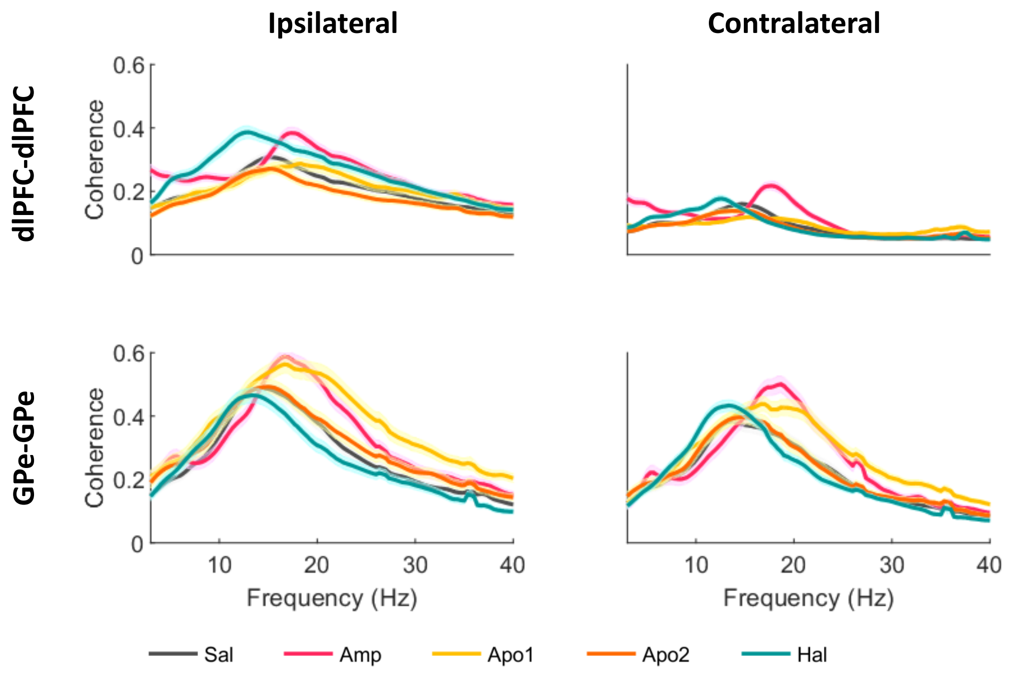

Supplementary Fig.12: Coherence in the ipsilateral pairs is greater than in the contralateral pairs in the dlPFC, but not in the GPe. Shadow indicates standard error of the mean. Source data are provided as a Source Data file. dlPFC dorsolateral prefrontal cortex, GPe globus pallidis pars externa, Sal: saline, Amp: amphetamine, Apo: apomorphine, Hal: haloperidol.

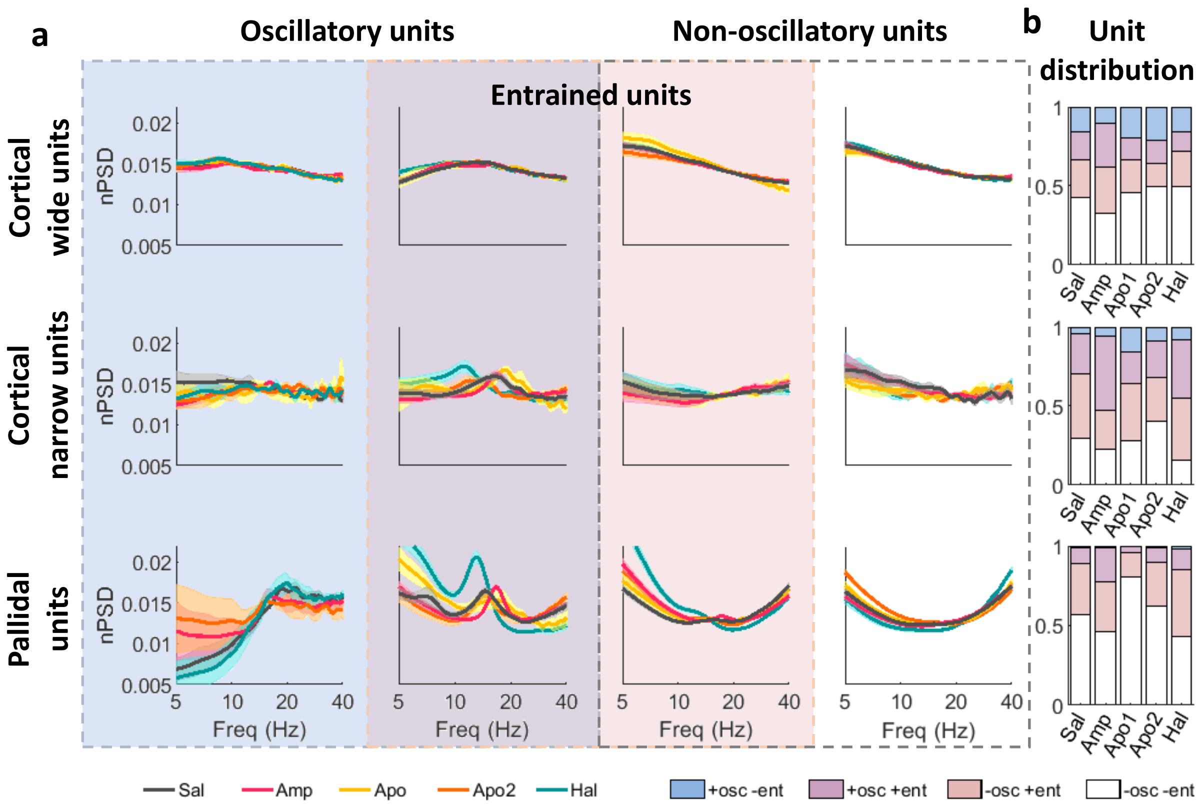

Supplementary Fig.13. Spectral activity of single units grouped by their oscillatory and LFP-entrainment classification. Units were classified in two independent processes as oscillatory or not, and as entrained to LFP beta activity or not (see methods). (A) Average normalized PSD (nPSD) of oscillatory and not entrained units (first column), oscillatory and entrained units (second column), non-oscillatory and entrained units (third column), non-oscillatory and not entrained units (fourth column). (B) Distribution of units into the aforementioned groups. Note the higher sensitivity of the entrainment analysis relative to the oscillation analysis. Entrainment analysis was based on LFP phase during spike occurrence, while oscillation analysis was based on beta peak prominence in the power spectrum. Top row: cortical wide units. Middle row: cortical narrow units. Bottom row: pallidal units. Shadow indicates standard error of the mean. Source data are provided as a Source Data file. Sal: saline, Amp: amphetamine, Apo: apomorphine, Hal: haloperidol, nPSD: normalized power spectral density, osc: oscillatory, ent: entrained, freq: frequency.

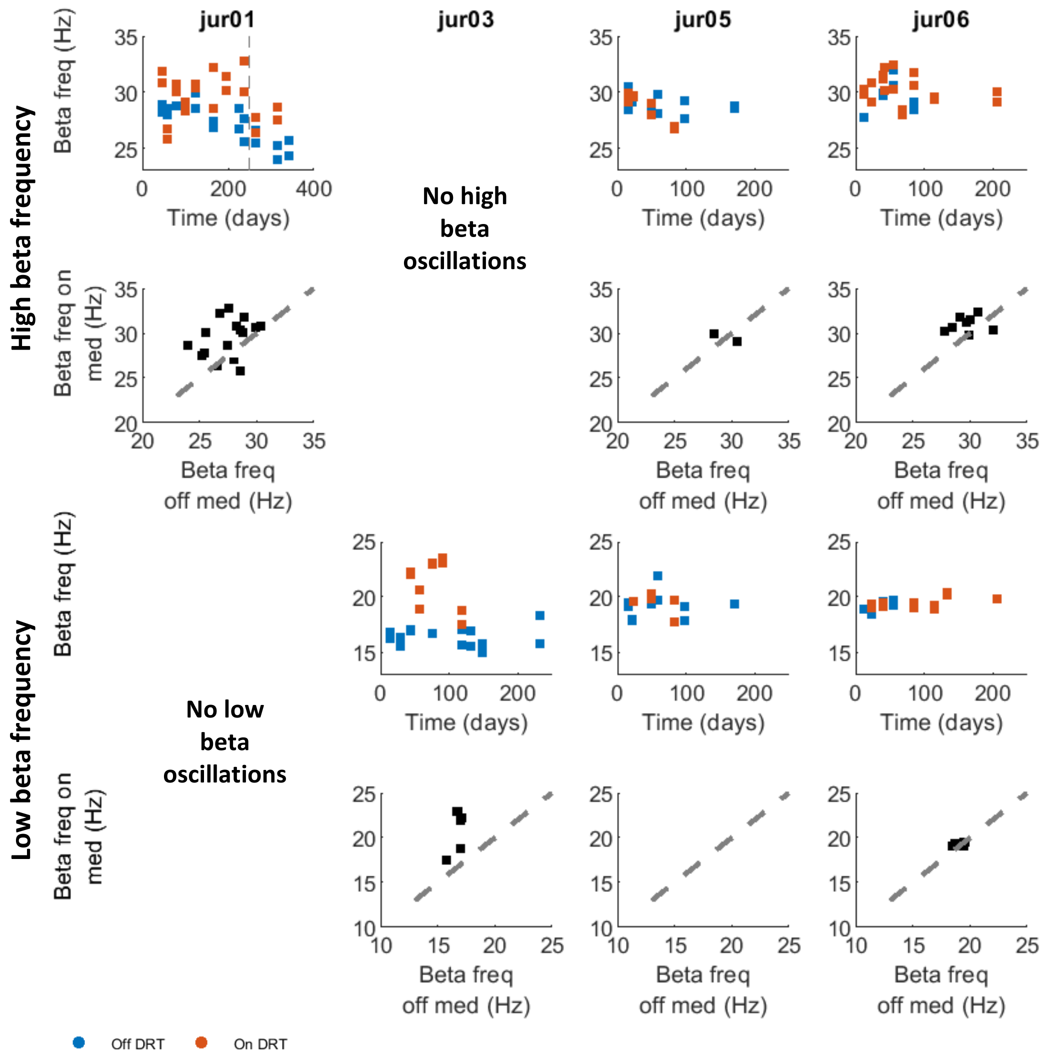

Supplementary Fig.14. Effect of acute and chronic dopamine modulation on LFP beta frequency in individual patients with PD. Each column shows data of a single patient. First row: Frequency of beta peak in the high beta domain as a function of time post-surgery. Each point represents average per day of beta peak frequency in one STN on (red) and off (blue) DRT. Second row: Comparison of beta frequency on and off DRT. Each point represents average per day of beta peak frequency in one STN in days with both off and on DRT sessions. X axis – off DRT. Y axis – on DRT. Clustering of data-points above the diagonal line indicates a shift up in beta frequency in the on DRT condition relative to the off DRT condition. Third row: same as first row for low beta domain. Fourth row: same as second row for low beta domain. Patients can exhibit a peak in one or both beta domains. Gray dashed line indicates day 250 post-surgery. Recordings after this day were not included in the model to avoid exaggerated influence of jur01 data on MLEM results. Source data are provided as a Source Data file. freq: frequency, med:medication.

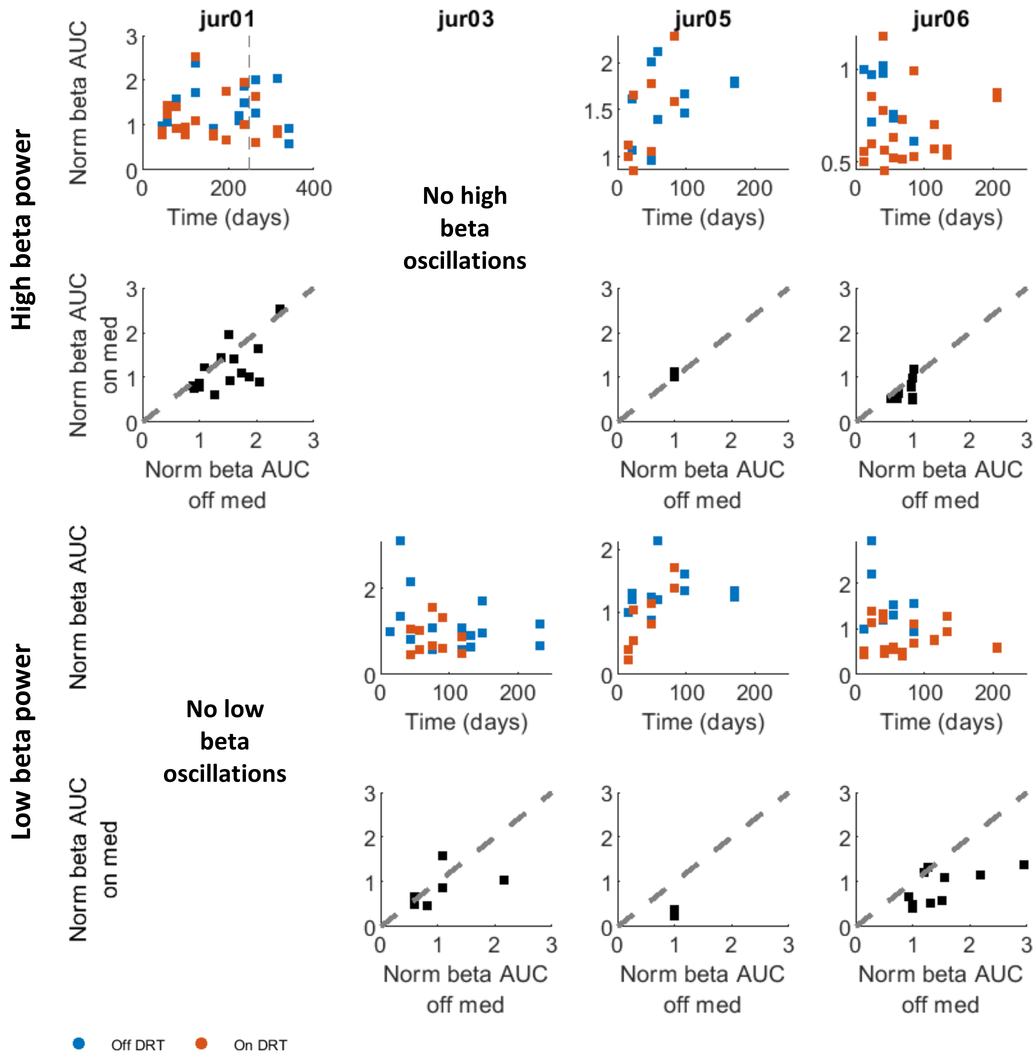

Fig S15. Effect of acute and chronic dopamine modulation on LFP beta power in individual patients with PD. Beta power was evaluated as area under the curve (AUC) of the normalized PSD (nPSD) in the high and low beta domains. AUC values were normalized relative to those collected during the first recording day after the surgery. Each column shows data of a single patient. First row: Beta power in the high-beta domain as a function of time post-surgery. Each point represents average per day of high-beta AUC on (red) and off (blue) DRT. Second row: Comparison of beta power on and off DRT. Each point represents average high-beta AUC in days with both off and on DRT sessions. X axis – off DRT. Y axis – on DRT. Clustering of data-points below the diagonal line indicates a decrease in beta power in the on DRT condition relative to the off DRT condition. Third row: same as first row for low beta domain. Fourth row: same as second row for low beta domain. Patients can exhibit a peak in one or both beta domains. Gray dashed line indicates day 250 post-surgery. Recordings after this day were not included in the model to avoid exaggerated influence of jur01 data on MLEM results. Source data are provided as a Source Data file. Norm: normalized, AUC: area under the curve, med: medication.

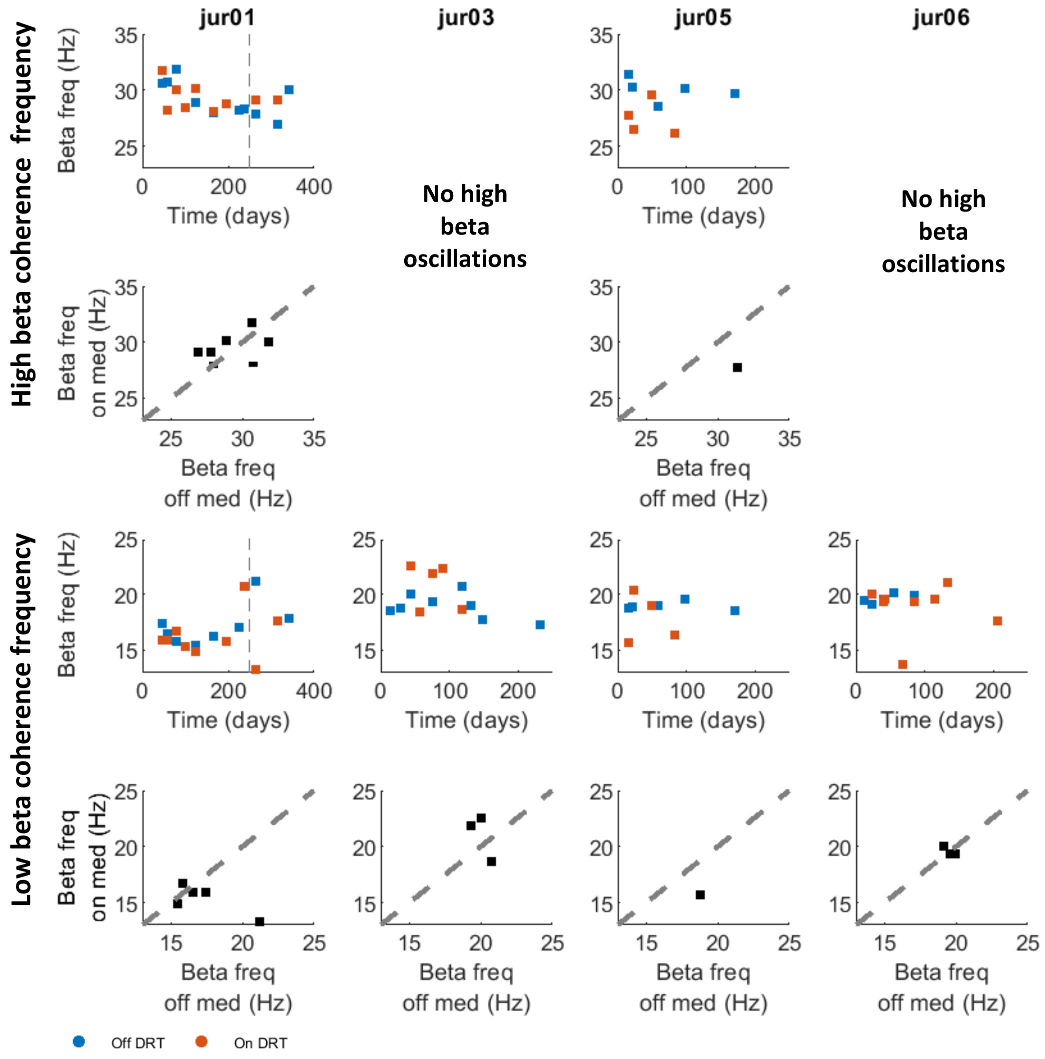

Supplementary Fig.16. Effect of acute and chronic dopamine modulation on LFP coherence beta frequency in individual patients with PD. Each column shows data of a single patient. First row: Frequency of beta coherence peak in the high beta domain as a function of time post-surgery. Each point represents average per day of beta coherence peak frequency on (red) and off (blue) DRT. Second row: Comparison of beta coherence frequency on and off DRT. Each dot represents average of beta coherence frequency in the high beta domain in days with both off and on DRT sessions. X axis – off DRT. Y axis – on DRT. Clustering of data-points above the diagonal line indicates a shift up in beta coherence frequency in the on DRT condition relative to the off DRT condition. Third row: same as first row for low beta domain. Fourth row: same as second row for low beta domain. Patients can exhibit a peak in one or both beta domains. Gray dashed line indicates day 250 post-surgery. Recordings after this day were not included in the model to avoid exaggerated influence of jur01 data on MLEM results. Source data are provided as a Source Data file. freq: frequency, med: medication.

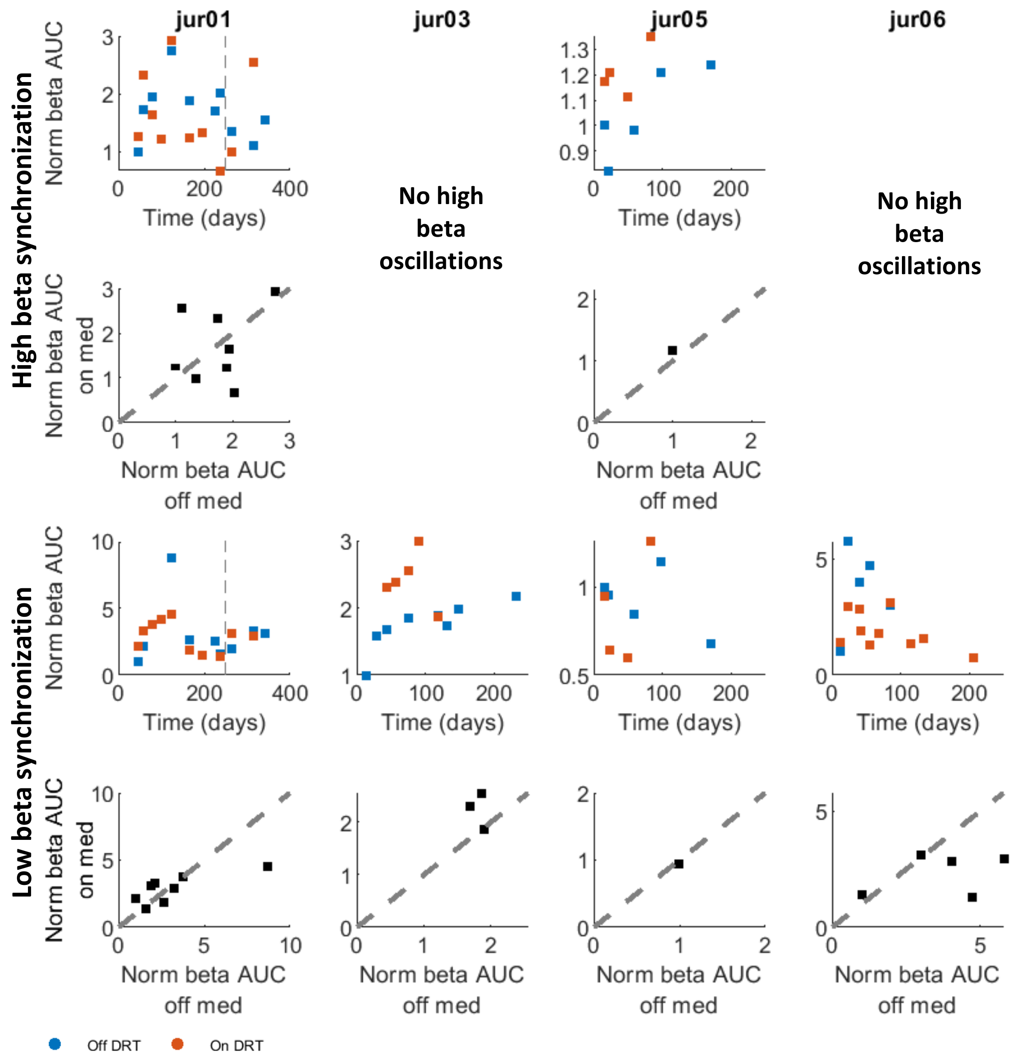

Supplementary Fig.17. Effect of acute and chronic dopamine modulation on beta synchrony in individual patients with PD. Beta synchrony is evaluated as area under the curve (AUC) of the coherence in the high and low beta domains. AUC values were normalized relative to those collected during the first recording day after the surgery. Each column shows data of a single patient. First row: Beta synchrony in the high beta domain as a function of time post-surgery. Each point represents average per day of normalized beta AUC on (red) and off (blue) DRT. Second row: Comparison of beta synchrony on and off DRT. Each dot represents average per day of normalized beta AUC in the high-beta domain in days with both off and on DRT sessions. X axis – off DRT. Y axis – on DRT. Clustering of data-points below the diagonal line indicates a decrease in beta synchrony in the on DRT condition relative to the off DRT condition. Third row: same as first row for low beta domain. Fourth row: same as second row for low beta domain. Patients can exhibit a peak in one or both beta domains. Gray dashed line indicates day 250 post-surgery. Recordings after this day were not included in the model to avoid exaggerated influence of jur01 data on MLEM results. Source data are provided as a Source Data file. AUC: area under the curve, med: medication, Norm: normalized.

Supplementary Tables

a

			Sal	Amp	Apo1	Apo2	Hal
LFP sites	Monkey G	dlPFC	54	59	46	47	53
		GPe	68	52	55	54	58
	Monkey D	dlPFC	87	58	78	78	51
		GPe	58	41	40	40	53
	Total	dlPFC	141	117	124	125	104
		GPe	126	93	95	94	91
Single units	Monkey G	wide	198	221	61	153	221
		narrow	27	27	8	19	30
		HFD	267	253	72	185	175
	Monkey D	wide	298	154	67	151	191
		narrow	61	47	17	50	32
		HFD	172	130	50	137	186
	Total	wide	496	375	128	304	412
		narrow	88	74	25	69	62
		HFD	439	383	122	322	361

b

		naive	MPTP
LFP sites	Monkey G	15	15
	Monkey D	26	9
	Total	41	24
Single units	Monkey G	33	35
	Monkey D	54	53
	Total	87	88

Supplementary Table 1. Number of LFP sites and single units in the NHP dataset. (a) Acute dopamine modulation experiment. (b) Chronic dopamine modulation experiment. LFP: local fiels potential, Sal: saline, Amp: amphetamine, Apo: apomorphine, Hal: haloperidol.

Pts.	Number of recording days OFF/ON (both) DRT	Number of recording sessions OFF/ON DRT	Number of observations (sessions*sites) OFF/ON DRT
Jur 01	10/11 (8)	13/19	156/228
Jur 03	8/5 (3)	10/6	120/72
Jur 05	6/4 (1)	9/4	81/36
Jur 06	5/10 (5)	11/19	132/228
Total	29/30 (17)	43/48	489/564

Supplementary Table 2. Patient recording dataset. Each recording day has either only on DRT recording sessions, only off DRT sessions, or both. In column two, days with on and off sessions are counted both as off day and as an on day. Pts: patients, DRT: dopamine replacement therapy.

a

		Sal	Amp	Apo1	Apo2	Hal
Pupil size	mean	0.29	0.97	0.63	0.07	0.47
	SD	0.38	0.64	0.5	0.48	0.84
Saccade frequency	Mean	1.38	3.38	1.87	1	0.13
	SD	0.73	1.27	1.19	0.69	0.15
Saccade amplitude	mean	124.36	175.85	131.55	121.12	76.25
	SD	20.96	23.22	15.33	16.72	26.18
Eye closed probability	mean	0.19	0.01	0.1	0.32	0.61
	SD	0.12	0.04	0.1	0.15	0.2
Blink frequency	mean	12.81	10.08	24.68	12.92	11.66
	SD	4.41	5.23	6.7	5.42	5.79

b

	F	df1	df2	p value	η^2
Pupil size	38.78	4	211	2.6e-24	0.42
Saccade frequency	74.68	4	218	9.0e-40	0.58
Saccade amplitude	119.94	4	219	5.1e-54	0.69
Eye closed probability	131.24	4	217	9.1e-57	0.71
Blink frequency	47.13	4	216	1.9e-28	0.47

c

		Pupil size		Saccade frequency		Saccade amplitude		Eye closed probability		Blink frequency	
		p	g	p	g	P	g	p	g	P	g
Sal	Amp	**8.3e-8**	**-1.32**	**9.9e-9**	**-1.99**	**9.9e-9**	**-2.33**	**1.0e-8**	**1.94**	0.0998	0.57
Sal	Apo1	**0.0319**	**-0.78**	0.0591	-0.51	0.4346	-0.38	**0.0140**	**0.75**	**9.9e-9**	**-2.15**
Sal	Apo2	**0.0182**	**0.85**	0.2169	0.54	0.9410	0.17	**1.2e-5**	**-0.97**	1.0000	-0.02
Sal	Hal	**1.3e-8**	**1.26**	**1.0e-8**	**2.17**	**9.9e-9**	**2.05**	**9.9e-9**	**-2.65**	0.8506	0.23
Amp	Apo1	0.0579	0.58	**9.9e-9**	**1.22**	**9.9e-9**	**2.23**	**0.0049**	**-1.32**	**9.9e-9**	**-2.41**
Amp	Apo2	**9.9e-9**	**1.81**	**9.9e-9**	**2.03**	**9.9e-9**	**2.68**	**9.9e-9**	**-2.89**	0.1223	-0.53
Amp	Hal	**9.9e-9**	**1.93**	**9.9e-9**	**3.47**	**9.9e-9**	**4.00**	**9.9e-9**	**-4.23**	0.6926	-0.28
Apo1	Apo2	**3.3e-7**	**1.41**	**8.5e-5**	**0.89**	0.1465	0.64	**9.9e-9**	**-1.68**	**9.9e-9**	**1.91**
Apo1	Hal	**9.9e-9**	**1.60**	**9.9e-9**	**1.99**	**9.9e-9**	**2.57**	**9.9e-9**	**-3.19**	**9.9e-9**	**2.06**
Apo2	Hal	**0.0183**	**0.59**	**1.5e-4**	**1.69**	**9.9e-9**	**2.03**	**9.9e-9**	**-1.66**	0.8396	0.22

Supplementary Table 3. Drug effects on eye physiology. (a) Descriptive statistics (b) One-way ANOVA test results. Effect size was estimated by η^2 measurement (c) Post-hoc results. p: p value, result of Tukey post-hoc test. g: effect size estimated by Hedge's g. Comparisons that did not reach statistical significance and didn't require post-hoc test are marked with a dash. Results are presented in Fig.S2. Source data are provided as a Source Data file. Sal: saline, Amp: amphetamine, Apo: apomorphine, Hal: haloperidol,

a

		dlPFC					GPe					STN	
		Sal	Amp	Apo1	Apo2	Hal	Sal	Amp	Apo1	Apo2	Hal	naive	MPTP
LFP beta freq	mean	14.41	16.74	15.71	13.52	12.54	14.47	16.84	15.82	14.51	12.34	13.80	10.88
	SD	2.8	1.48	3.5	2.25	2.95	1.72	1.07	3.08	2.18	1.08	2.44	2.13
LFP Beta AUC	mean	0.185	0.157	0.16	0.168	0.192	0.203	0.188	0.188	0.2	0.209	0.10	0.12
	SD	0.046	0.038	0.04	0.046	0.047	0.055	0.055	0.059	0.063	0.05	0.023	0.028
LFP beta peak	mean	0.012	0.009	0.008	0.011	0.014	0.019	0.022	0.015	0.019	0.023	0.0080	0.0149
	SD	0.006	0.004	0.003	0.006	0.009	0.008	0.013	0.007	0.009	0.011	0.0035	0.0067
LFP beta peak (top 20%)	Mean						0.032	0.041	0.024	0.031	0.040		
	SD						0.005	0.004	0.004	0.002	0.005		

b

	dlPFC					GPe					STN				
	χ^2	df	p	η^2	BF	χ^2	df	P	η^2	BF	t	df	p	g	BF
LFP beta freq	**185.13**	**(4,522)**	**5.9e-39**	**0.23**	**1.1e+25**	**228.69**	**(4,486)**	**2.5e-48**	**0.35**	**2.1e+41**	**4.27**	**43**	**1.1e-4**	**1.25**	**226.75**
LFP Beta AUC	**55.54**	**(4,606)**	**2.5e-11**	**0.09**	**3.9e+8**	8.98	(4,494)	0.0616	0.02	0.071	**-3.28**	**63**	**0.0017**	**-0.83**	**16.16**
LFP beta peak	**59.36**	**(4,606)**	**3.9e-12**	**0.12**	**6.7e+12**	**30.02**	**(4,494)**	**4.8e-6**	**0.07**	**4.1e+4**	**-5.50**	**63**	**7.3e-7**	**-1.4**	**2.1e+4**
LFP beta peak (top 20%)						**72.85**	**(4,95)**	**5.7e-15**	**0.69**	**2.9e+20**					

c

		dlPFC									GPe									
		LFP beta freq.			LFP beta AUC			LFP beta peak			LFP beta freq.			LFP beta AUC	LFP beta peak			LFP beta peak (top 20%)		
		p	g	BF	p	g	BF	p	g	BF	p	g	BF		p	g	BF	p	g	BF
Sal	Amp	**9.9e-9**	**-1.01**	**3.3e+10**	**2.5e-6**	**0.67**	**4.6e+4**	**0.003**	**0.56**	**1.1e+3**	**9.9e-9**	**-1.60**	**5.0e+21**		0.8195	-0.27	0.549	**1.6e-4**	**-1.92**	**5.1e+4**
Sal	Apo1	**0.023**	**-0.41**	**8.607**	**2.8e-5**	**0.57**	**2.1e+3**	**1.1e-8**	**0.85**	**2.4e+8**	**0.003**	**-0.56**	**270.43**		**0.003**	**0.59**	**640.63**	**0.033**	**1.73**	**8.5e+3**
Sal	Apo2	0.061	0.34	2.406	**0.017**	**0.36**	4.473	0.138	0.24	0.439	0.999	-0.02	0.077		1.000	0.03	0.078	0.991	0.32	0.284
Sal	Hal	**1.6e-8**	**0.65**	**1.6e+3**	0.875	-0.16	**0.155**	0.982	-0.29	0.869	**9.9e-9**	**1.42**	**1.4e+17**		0.3635	-0.35	1.945	**0.002**	**-1.45**	**634.70**
Amp	Apo1	**8.5e-4**	**0.38**	**3.555**	0.984	-0.09	**0.092**	0.067	0.40	8.019	**2.4e-4**	**0.44**	**6.723**	---.	**1.3e-4**	**0.73**	**8.9e+3**	**9.9e-9**	**3.99**	**3.2e+12**
Amp	Apo2	**9.9e-9**	**1.69**	**6.8e+23**	0.234	-0.28	**0.741**	0.696	-0.29	0.874	**9.9e-9**	**1.35**	**5.7e+13**		0.803	0.28	0.52	**1.8e-5**	**3.085**	**2.3e+9**
Amp	Hal	**9.9e-9**	**1.88**	**3.7e+24**	**1.3e-7**	**-0.85**	**5.3e+6**	**9.9e-4**	**-0.74**	**9.2e+4**	**9.9e-9**	**4.17**	**2.5e+63**		0.957	-0.05	0.087	0.977	0.27	0.244
Apo1	Apo2	**6.9e-7**	**0.74**	**4.8e+4**	0.524	-0.19	**0.224**	**6.7e-4**	**-0.58**	**1.7e+3**	**0.004**	**0.49**	**16.118**		**0.010**	**-0.52**	**41.021**	**0.113**	**-2.00**	**1.1e+5**
Apo1	Hal	**9.9e-9**	**0.97**	**1.4e+7**	**1.4e-6**	**-0.75**	**1.7e+5**	**1.1e-8**	**-0.96**	**1.1e+9**	**9.9e-9**	**1.48**	**9.3e+15**		**5.0e-6**	**-0.86**	**5.3e+5**	**1.0e-8**	**-3.21**	**6.6e+9**
apo2	Hal	**0.003**	**0.38**	**2.080**	**0.001**	**-0.52**	**110.98**	0.056	-0.48	37.882	**1.0e-8**	**1.25**	**3.5e+11**		0.373	-0.36	1.566	**2.8e-4**	**-2.16**	**5.2e+5**

Supplementary Table 4. Properties of LFP beta oscillations. (a) Descriptive statistics of LFP beta properties in NHP acute (dlPFC, GPe) and chronic (STN) dopamine modulation experiments (b) Results of Kruskal-Wallis test and two-sided student's t-test for the acute and chronic experiments, respectively. η^2 – effect size. (c) Post-hoc comparison results. p: p value, result of Tukey post-hoc test. g: effect size estimated by Hedge's g. BF: Bayes factor. Comparisons that did not reach statistical significance and didn't require post-hoc test are marked with ---. Results are presented in Fig. 2. Source data are provided as a Source Data file. LFP: local field potential, SD: standard deviation; dlPFC dorsolateral prefrontal cortex, GPe: globus pallidis pars externa, Sal: saline, Amp: amphetamine, Apo: apomorphine, Hal: haloperidol, df: degrees of freedom.

a

		dlPFC LFP sites	GPe LFP sites	Cortical wide units	Cortical narrow units	Pallidal units
Oscillatory site/unit probability	sal	0.90	1.00	0.35	0.30	0.11
	amp	0.94	0.99	0.38	0.53	0.22
	apo1	0.81	1.00	0.34	0.36	0.04
	apo2	0.83	0.97	0.36	0.32	0.10
	hal	0.78	0.96	0.28	0.45	0.15

b

		STN LFP sites	STN units
Oscillatory site/unit probability	Naïve	0.56	0.24
	MPTP	0.875	0.57

c

		experiment	X^2	df	P value	Effect size
LFP	dlPFC	Acure	**16.35**	**4**	**0.0026**	**0.16**
	GPe	Acute	**9.77**	**4**	**0.0446**	**0.14**
	STN	Chronic	**6.83**	**1**	**0.0090**	**0.32**
Single units	Cortical wide	Acute	9.47	4	0.0505	0.07
	Cortical narrow	Acute	**11.70**	**4**	**0.0197**	**0.19**
	Pallidal	Acute	**38.75**	**4**	**7.8e-8**	**0.15**
	Subthalamic	Chronic	**19.38**	**1**	**1.1e-5**	**0.33**

d

		dlPFC		GPe		Cortical wide units		Cortical narrow units		Pallidal units	
		p	Φ	p	Φ	p	Φ	p	Φ	p	Φ
sal	amp	1.0000	-0.07	1.0000	0.08	---		**0.0274**	**-0.24**	**3.0e-4**	**-0.15**
sal	apo1	0.4339	-0.12	--	--			1.0000	0.06	0.1659	-0.10
sal	apo2	0.9802	-0.10	0.4347	-0.14			1.0000	0.03	1.0000	-0.02
sal	hal	0.0846	0.17	0.1753	-0.16			0.4975	-0.16	1.0000	0.06
amp	apo1	**0.0315**	**-0.19**	1.0000	0.07			1.0000	-0.15	1.0e-4	-0.20
amp	apo2	0.0857	-0.17	1.0000	-0.07			0.1189	-0.21	1.0e-4	-0.16
amp	hal	**0.0047**	**-0.24**	1.0000	-0.10			1.0000	-0.08	0.1523	-0.09
apo1	apo2	1.0000	0.02	0.7922	-0.13			1.0000	0.04	0.4688	-0.09
apo1	hal	1.0000	0.04	0.3885	-0.15			1.0000	-0.08	0.0126	0.15
apo2	hal	1.0000	0.07	1.0000	-0.03			1.0000	-0.14	0.3818	0.08

Supplementary Table 5. Probability of oscillatory LFP sites/single units. (a) Descriptive statistics, acute dopamine modulation experiment. (b) Descriptive statistics, chronic dopamine modulation experiment. (c) Chi-square test results. Effect size was estimated by Cramer's V for the acute modulation experiment, and Φ for the chronic modulation experiment (d) Results of post-hoc comparison with Bonferroni correction for multiple comparisons. p: p-value of 2x2 Chi-square test with drug condition and oscillation status (oscillatory vs non-oscillatory) as factors. Φ: effect size. Results are presented in Figures S3 (LFP) and S7 (SUA). Source data are provided as a Source Data file. LFP: local field potential, dlPFC dorsolateral prefrontal cortex, GPe globus pallidis pars externa, STN: subthalamic nucleus, Sal: saline, Amp: amphetamine, Apo: apomorphine, Hal: haloperidol.

a

			F	DF	sig	Partial η^2
dlPFC	Beta frequency	Eye state	21.486	(1,137)	8.0e-6	0.136
		Drug	4.811	(4,137)	0.001	0.123
		Eye state*drug	5.912	(4,137)	2.0e-4	0.147
GPe		Eye state	48.392	(1,181)	6.2e-11	0.211
		Drug	5.931	(4,181)	1.6e-4	0.116
		Eye state*drug	21.820	(4,181)	1.0e-14	0.325
dlPFC	Beta peak	Eye state	0.791	(1,560)	0.374	0.001
		Drug	7.995	(4,560)	3.0e-6	0.054
		Eye state*drug	19.597	(4,560)	4.1e-15	0.123
GPe		Eye state	9.768	(1,409)	0.002	0.023
		Drug	7.418	(4,409)	9.0e-6	0.068
		Eye state*drug	13.034	(4,409)	5.3e-10	0.113
dlPFC	Beta AUC	Eye state	50.084	(1,560)	4.4e-12	0.082
		Drug	7.127	(4,560)	1.3e-5	0.048
		Eye state*drug	6.713	(4,560)	2.8e-5	0.046
GPe		Eye state	18.158	(1,409)	2.5e-5	0.043
		Drug	4.648	(4,409)	0.001	0.043
		Eye state*drug	13.189	(4,409)	4.1e-10	0.114

b

		dlPFC									GPe								
		Beta frequency			Beta peak			Beta AUC			Beta frequency			Beta peak			Beta AUC		
Drug		mean	ste	P (o-c)	mean	Ste	P (o-c)	mean	ste	P (o-c)	mean	Ste	P (o-c)	mean	ste	P (o-c)	mean	ste	P (o-c)
Sal	o	14.45	0.47	0.035	.010	.001	0.570	.153	.004	2.1e-5	15.25	0.28	6.2e-16	.015	.002	0.078	.166	.005	6.2e-15
	c	13.48	0.62		.009	.001		.163	.004		13.25	0.31		.014	.001		.184	.005	
Amp	o	17.08	0.60	2.1e-5	.007	.001	8.3e-11	.124	.007	7.8e-5	18.50	1.12	0.162	.009	.001	0.327	.129	.012	0.330
	c	14.50	0.79		.011	.001		.138	.006		17.25	1.22		.010	.001		.124	.011	
Apo1	o	15.92	0.55	4.0e-4	.007	.001	0.098	.136	.005	1.6e-10	16.78	0.32	7.5e-28	.012	.001	0.020	.165	.006	6.5e-9
	c	13.97	0.72		.008	.001		.152	.004		13.46	0.35		.013	.001		.181	.006	
Apo2	o	13.88	0.52	0.145	.009	.001	4.5e-4	.140	.005	0.397	14.99	0.34	2.2e-5	.017	.001	7.4e-11	.178	.006	0.333
	c	13.14	0.69		.007	.001		.142	.004		13.79	0.38		.013	.001		.175	.006	
Hal	o	12.01	0.47	0.102	.012	.001	1.0e-5	.163	.004	0.711	13.02	0.42	0.159	.019	.001	2.0e-6	.178	.006	0.081
	c	12.85	0.62		.010	.001		.164	.004		13.50	0.46		.016	.001		.183	.006	

Supplementary Table 6. Beta properties as a function of drug condition and eye-state. (a) Two-way mixed-design ANOVA results with beta properties as dependent factors, drug condition as between-observation independent factor, and eye-state as within-observation independent factor. Eye state*drug marks the interaction effect. The test was conducted separately for each beta property. Only sites that were oscillatory in both eye-state conditions were included in the beta frequency analysis. (b) Post-hoc results. Post-hoc test compared the beta properties between the two eye-states (open vs closed) within each drug condition, and used Bonferroni correction for multiple comparisons. Source data are provided as a Source Data file. o: open; c: closed; STE: standard error of the mean. dlPFC dorsolateral prefrontal cortex, GPe globus pallidis pars externa, Sal: saline, Amp: amphetamine, Apo: apomorphine, Hal: haloperidol, AUC: area under the curve, F: frequency, DF: degrees of freedom; o-c: open-closed, sig: significance

a

		Cortical wide units					Cortical narrow units					Pallidal units					STN units	
		sal	amp	apo1	apo2	hal	sal	amp	apo1	apo2	hal	sal	amp	apo1	apo2	hal	Naïve	MPTP
Firing rate	Mean	2.50	3.10	2.18	2.18	2.33	7.59	6.61	9.05	7.12	9.51	59.02	66.32	74.53	60.08	52.17	23.50	29.95
	SD	2.86	3.06	2.35	2.41	2.45	6.28	5.41	8.32	6.55	6.72	24.89	31.12	26.55	24.45	25.45	11.72	17.26
SUA beta freq. (osc)	Mean	14.18	14.71	15.36	14.38	13.71	16.73	15.82	18.17	14.2	12.7	15.87	17.04	17.80	14.84	13.65	17.49	11.52
	SD	4.27	4.50	4.71	4.60	4.30	4.45	3.69	3.19	3.5	3.13	2.59	1.66	3.82	1.82	1.86	4.06	2.72
SUA beta freq. (all)	Mean	16.04	16.33	16.43	15.91	16.23	16.62	16.88	18.56	16.08	14.42	16.79	17.34	17.51	16.76	15.84	17.36	14.09
	SD	4.53	4.66	4.78	4.53	4.66	4.48	4.34	4.05	4.53	4.60	3.52	2.98	3.72	3.66	4.25	3.94	4.98
SUA beta AUC	Mean	0.078	0.078	0.081	0.08	0.08	0.071	0.071	0.07	0.066	0.072	0.043	0.043	0.033	0.04	0.045	0.021	0.024
	SD	0.016	0.012	0.016	0.015	0.016	0.017	0.006	0.014	0.011	0.017	0.013	0.013	0.01	0.012	0.017	0.005	0.007
SUA beta peak	Mean	0.0028	0.0028	0.003	0.0029	0.0029	0.0026	0.0026	0.0026	0.0024	0.0028	0.0014	0.0015	0.0011	0.0013	0.0016	0.0014	0.0019
	SD	0.0007	0.0005	0.0008	0.0007	0.0008	0.0009	0.0005	0.0006	0.0005	0.0009	0.0006	0.0006	0.0004	0.0005	0.0009	0.0004	0.0009

b

	Cortical wide units					Cortical narrow units					Pallidal units					STN units				
	χ^2	df	p	η^2	BF	χ^2	df	p	η^2	BF	χ^2	df	p	η^2	BF	t	df	p	g	BF
Firing rate	**31.56**	**(4,1710)**	**2.4e-6**	**0.008**	**24.44**	7.78	(4,313)	0.1001	0.022	23.94	**80.94**	**(4,1622)**	**1.1e-16**	**0.05**	**24.14**	**-2.89**	**173**	**0.0043**	**-0.44**	**4.68**
SUA beta freq. (osc)	5.58	(4,570)	0.2330	0.010	7.5e-3	**23.76**	**(4,119)**	**8.9e-5**	**0.179**	**1.7e+2**	**105.52**	**(4,222)**	**6.6e-22**	**0.32**	**1.8e+15**	**7.25**	**69**	**4.6e-10**	**1.87**	**2.3e+7**
SUA beta freq. (all)	2.35	(4,1681)	0.6715	0.001	2.0e-4	**18.35**	**(4,298)**	**0.001**	**0.059**	**5.6**	**52.36**	**(4,1495)**	**1.2e-10**	**0.02**	**1.6e+3**	**4.77**	**169**	**4.0e-6**	**0.73**	**3.1e+3**
SUA Beta AUC	5.59	(4,1710)	0.2316	0.005	3.6e-3	**12.66**	**(4,313)**	**0.0130**	**0.024**	**0.046**	**76.72**	**(4,1622)**	**8.6e-16**	**0.05**	**2.3e+12**	**-2.38**	**173**	**0.0186**	**-0.36**	**1.30**
SUA beta peak	**11.72**	**(4,1710)**	**0.0196**	**0.008**	**0.045**	8.23	(4,313)	0.0834	0.025	0.060	**64.29**	**(4,1622)**	**3.6e-13**	**0.04**	**8.4e+8**	**-4.85**	**173**	**2.7e-6**	**-0.73**	**4.5e+3**

c

		Cortical wide units									Cortical narrow units											Pallidal units														
		Firing rate			SUA beta freq. (osc)	SUA beta freq. (all)	SUA beta AUC	SUA beta peak			FR	SUA beta freq. (osc)			SUA beta freq. (all)			SUA beta AUC			SUA beta peak	Firing rate			SUA beta freq. (osc)			SUA beta freq. (all)			SUA beta AUC			SUA beta peak		
		p	g	BF				p	g	BF		p	g	BF	p	g	BF	p	g	BF		p	g	BF	p	g	BF	p	g	BF	p	g	BF	p	g	BF
Sal	Amp	**2.8e-4**	**-0.14**	**0.30**				0.965	0.10	0.11		0.988	0.22	0.20	0.996	-0.06	0.10	0.324	0.05	0.09		**0.023**	**-0.22**	**4.997**	**8.6e-4**	**-0.57**	**11.7**	**0.013**	**-0.17**	**0.61**	0.9975	-0.01	0.04	0.527	-0.08	0.07
Sal	Apo1	0.686	0.11	0.09				0.338	-0.20	0.35		0.769	-0.34	0.26	0.338	-0.44	0.61	1.000	0.06	0.11		**9.4e-7**	**-0.55**	**5.5e+4**	0.692	-0.70	0.44	0.224	-0.20	0.31	**9.9e-9**	**0.75**	**6.3e+9**	**1.2e-8**	**0.61**	**1.5e+6**
Sal	Apo2	0.872	0.08	0.07				0.119	-0.13	0.20		0.177	0.61	1.30	0.965	0.12	0.12	0.361	0.35	0.94		0.982	-0.03	0.045	0.276	0.44	0.745	0.998	0.01	0.04	**0.017**	**0.25**	**12.13**	**0.040**	**0.22**	**3.02**
Sal	Hal	1.000	0.03	0.04				0.996	-0.05	0.05		**0.003**	**1.04**	**82.8**	**0.033**	**0.48**	**4.61**	0.993	-0.02	0.09		**6.0e-4**	**0.29**	**188.95**	**2.4e-6**	**0.99**	**8.8e+3**	**6.3e-4**	**0.25**	**9.15**	0.6135	-0.17	0.70	0.994	-0.20	1.86
Amp	Apo1	**4.7e-4**	**0.29**	**2.68**				0.163	-0.34	13.11		0.524	-0.64	0.66	0.514	-0.39	0.43	0.758	0.04	0.11		**0.010**	**-0.27**	**1.621**	1.000	-0.42	0.18	1.000	-0.05	0.06	**9.9e-9**	**0.73**	**6.6e+8**	**9.9e-9**	**0.67**	**1.9e+7**
Amp	Apo2	**3.9e-5**	**0.24**	**6.32**	---	---	---	**0.037**	**-0.26**	**10.52**	---	0.300	0.44	0.52	0.853	0.18	0.16	**0.004**	**0.50**	**6.46**	---	0.166	0.19	0.938	**3.0e-7**	**1.28**	**1.2e+6**	**0.011**	**0.18**	**0.53**	**0.009**	**0.25**	**9.05**	**3.0e-4**	**0.29**	**61.15**
Amp	Hal	**5.5e-4**	**0.19**	**1.32**				0.860	-0.14	0.31		**0.005**	**0.89**	**44.8**	**0.014**	**0.55**	**10.90**	0.685	-0.09	0.11		**1.0e-8**	**0.49**	**9.9e+7**	**9.9e-9**	**1.94**	**1.8e+18**	**1.0e-8**	**0.41**	**4.5e+4**	0.825	-0.16	0.43	0.8135	-0.13	0.203
Apo1	Apo2	0.980	-0.05	0.06				1.000	0.08	0.07		**0.046**	**1.13**	**6.45**	0.153	0.56	1.60	0.659	0.34	0.33		**1.9e-5**	**0.52**	**6.0e+3**	0.177	1.35	5.81	0.173	0.20	0.31	**2.4e-5**	**-0.54**	**1.4e+4**	**3.2e-4**	**-0.44**	**202.1**
Apo1	Hal	0.709	-0.11	0.09				0.514	0.15	0.14		**0.002**	**1.70**	**374.5**	**0.001**	**0.92**	**90.8**	1.000	-0.09	0.13		**9.9e-9**	**0.87**	**4.0e+12**	**0.005**	**2.00**	**356.86**	**1.9e-5**	**0.41**	**49.83**	**9.9e-9**	**-0.77**	**5.1e+9**	**1.1e-8**	**-0.60**	**3.3e+5**
Apo2	Hal	0.892	-0.06	0.06				0.285	0.08	0.07		0.763	0.45	0.51	0.208	0.36	0.74	0.226	-0.40	1.21		**2.2e-4**	**0.33**	**366.6**	0.09	0.65	6.26	**0.006**	**0.23**	**2.75**	**1.9e-4**	**-0.38**	**9.1e+3**	**0.018**	**-0.36**	**1.9e+3**

Table S7. Firing rate and beta properties of single unit activity. (a) Descriptive statistics of single unit properties in NHP acute (cortical and pallidal units) and chronic (STN units) dopamine modulation experiments (b) Results of Kruskal-Wallis test and two-sided student's t-test for the acute and chronic modulation experiments, respectively . η^2 – effect size. (c) Post-hoc comparison results. p - p value, result of Tukey post-hoc test. g – effect size estimated by Hedge's g. BF: Bayes factor. Comparisons that did not reach statistical significance and didn't require post-hoc test are marked with ---. Results are presented in Fig. 3 and Fig. S6. Source data are provided as a Source Data file. SUA: sngle unit activity, AUC: area under the curve, osc: oscillatory, dlPFC dorsolateral prefrontal cortex, GPe globus pallidis pars externa, Sal: saline, Amp: amphetamine, Apo: apomorphine, Hal: haloperidol

a

		LFP				Single units					
		dlPFC		GPe		Cortical wide		Cortical narrow		Pallidal	
		drug naïve	Sal	Drug naive	Sal	Drug naïve	Sal	Drug naive	Sal	Drug naïve	Sal
SUA beta freq. (osc)	Mean	16.77	14.41	15.69	14.47	15.20	14.18	16.65	16.73	15.32	15.87
	SD	2.93	2.80	2.38	1.72	4.38	4.27	3.95	4.45	3.02	2.59
SUA beta freq. (all)	Mean	16.77	14.41	15.66	14.47	16.40	16.04	16.95	16.62	16.58	3.92
	SD	2.93	2.80	2.41	1.72	4.55	4.53	4.38	4.48	16.79	3.52
SUA beta AUC	Mean	0.186	0.185	0.191	0.203	0.077	0.078	0.071	0.071	0.040	0.043
	SD	0.040	0.046	0.059	0.055	0.013	0.0155	0.014	0.017	0.013	0.013
SUA beta peak	Mean	0.0105	0.0123	0.0181	0.0194	0.0028	0.0028	0.0026	0.0026	0.0013	0.0014
	SD	0.0052	0.0062	0.0109	0.0084	0.0006	0.0007	0.0008	0.0009	0.0005	0.0006

b

	LFP								Single units											
	dlPFC				GPe				Cortical wide				Cortical narrow				Pallidal			
	t	df	p	g	t	df	p	g	t	df	p	g	t	df	p	g	t	df	P	g
SUA beta freq. (osc)	**6.30**	**232**	**1.5e-9**	**0.82**	**5.17**	**389**	**3.8e-7**	**0.56**	**2.52**	**557**	**0.0120**	**0.23**	-0.09	167	0.9288	-0.02	-1.05	121	0.2976	-0.19
SUA beta freq. (all)	**6.30**	**232**	**1.5e-9**	**0.82**	**4.99**	**391**	**9.1e-7**	**0.54**	1.40	1443	0.1617	0.08	0.58	349	0.5632	0.07	-0.92	1421	0.3591	-0.05
SUA Beta AUC	0.265	252	0.7913	0.03	-1.92	398	0.0554	-0.21	-1.90	1463	0.0576	-0.10	0.19	358	0.8477	-0.02	**-3.74**	**1551**	**0.0002**	**-0.21**
SUA beta peak	**-2.505**	**252**	**0.0129**	**0.32**	-1.14	398	0.2559	-0.12	-1.73	1463	0.0841	-0.09	0.59	358	0.5554	0.07	**-4.13**	**1551**	**3.8e-5**	**-0.23**

Table S8. Comparison of NHP LFP and single unit beta properties in drug-naïve and control-saline conditions. (a) Descriptive statistics (b) Results of two-samples student's t-test. p – p value. g – effect size estimated by Hedge's g. Results are presented in Fig.S8 and S9. Source data are provided as a Source Data file. SUA: single unit activity, freq: frequency, AUC: area under the curve, dlPFC dorsolateral prefrontal cortex, GPe: globus pallidis pars externa, Sal: saline, Amp: amphetamine, Apo: apomorphine, Hal: haloperidol, osc: iscillatory, SD: standard deviation,

a

		dlPFC-dlPFC					GPe-GPe					dlPFC-GPe				
		Sal	Amp	Apo1	Apo2	Hal	Sal	Amp	Apo1	Apo2	Hal	Sal	Amp	Apo1	Apo2	Hal
LFP coherence beta frequency	mean	15.39	17.3	15.89	15.16	13.74	14.96	17.53	16.48	15.23	13.19	14.28	16.69	14.77	13.49	13.09
	SD	2.07	1.73	3	2.55	2.09	1.73	1.29	2.56	2	0.62	2.12	1.38	2.53	1.18	1.8
LFP coherence beta AUC	mean	4.648	6.028	4.401	4.182	5.804	8.3	9.993	9.79	8.807	7.988	3.055	3.023	3.708	3.526	2.841
	SD	3.755	4.023	3.538	3.275	4.267	4.677	4.722	5.306	4.746	3.973	1.395	1.347	1.894	1.66	1.456
LFP coherence beta peak	mean	0.247	0.333	0.22	0.217	0.302	0.453	0.563	0.504	0.461	0.453	0.209	0.261	0.25	0.256	0.204
	SD	0.17	0.177	0.161	0.152	0.191	0.206	0.184	0.215	0.19	0.175	0.108	0.12	0.117	0.111	0.144

b

	dlPFC-dlPFC				GPe-GPe				dlPFC-GPe			
	χ^2	df	p	η^2	χ^2	df	P	η^2	χ^2	df	P	η^2
LFP beta frequency	**281.07**	**(4,1157)**	**1.3e-59**	**0.18**	**399.93**	**(4,740)**	**2.9e-85**	**0.47**	**213.52**	**(4,475)**	**4.6e-45**	**0.37**
LFP Beta AUC	**43.64**	**(4,1157)**	**7.6e-9**	**0.03**	**26.55**	**(4,740)**	**2.4e-5**	**0.05**	**26.63**	**(4,475)**	**2.4e-5**	**0.05**
LFP beta peak	**75.84**	**(4,1157)**	**1.3e-15**	**0.06**	**41.09**	**(4,740)**	**2.6e-8**	**0.05**	**27.78**	**(4,475)**	**1.4e-5**	**0.04**

c

		dlPFC-dlPFC						Gpe-GPe						dlPFC-GPe					
		beta freq.		beta AUC		Beta peak		beta freq.		beta AUC		Beta peak		beta freq.		beta AUC		Beta peak	
		p	g	p	g	p	g	p	g	p	g	p	g	p	g	p	g	p	g
Sal	Amp	**9.9e-9**	**-0.98**	**5.6e-5**	**-0.36**	**6.0e-7**	**-0.50**	**9.9e-9**	**-1.64**	**0.033**	**-0.36**	**4.0e-5**	**-0.56**	**9.9e-9**	**-1.38**	1.000	0.02	**0.004**	**-0.45**
Sal	Apo1	**5.8e-5**	**-046**	0.975	0.01	0.405	0.14	**9.9e-9**	**-1.31**	**0.001**	**-0.47**	**0.001**	**-0.43**	**2.9e-5**	**-0.74**	**0.003**	**-0.48**	0.258	-0.31
Sal	Apo2	0.711	0.10	0.779	0.13	0.324	0.18	0.851	-0.14	0.964	-0.11	1.000	-0.04	0.160	0.44	0.260	-0.31	**0.040**	**-0.44**
Sal	Hal	**9.9e-9**	**0.79**	**0.004**	**-0.29**	**0.007**	**-0.31**	**9.9e-9**	**1.30**	0.980	0.07	0.999	-0.00	**7.8e-4**	**0.59**	0.780	0.15	0.791	0.04
Amp	Apo1	**1.2e-6**	**0.24**	**7.7e-4**	**0.36**	**1.0e-8**	**0.63**	0.837	-0.09	0.966	-0.14	0.871	0.08	**0.013**	**0.28**	**0.001**	**-0.52**	0.807	0.14
Amp	Apo2	**9.9e-9**	**0.95**	**3.8e-7**	**0.51**	**9.9e-9**	**0.71**	**9.9e-9**	**1.34**	0.197	0.25	**1.7e-4**	**0.55**	**9.9e-9**	**2.44**	0.184	-0.34	0.998	0.04
Amp	Hal	**9.9e-9**	**1.84**	0.835	0.05	0.282	0.17	**9.9e-9**	**4.40**	**0.010**	**0.46**	**3.5e-5**	**0.62**	**9.9e-9**	**2.32**	0.773	0.13	**1.2e-4**	**0.44**
Apo1	Apo2	**1.3e-7**	**0.51**	0.420	0.12	1.000	0.04	**9.9e-9**	**1.10**	**0.024**	**0.37**	**0.004**	**0.40**	**1.0e-8**	**1.20**	0.541	0.20	0.952	-0.10
Apo1	Hal	**9.9e-9**	**1.03**	**0.031**	**-0.30**	**9.3e-6**	**-0.44**	**9.9e-9**	**2.48**	**3.5e-4**	**0.56**	**8.8e-4**	**0.45**	**9.9e-9**	**1.25**	**9.4e-5**	**0.58**	**0.027**	**0.30**
Apo2	Hal	**1.1e-8**	**0.60**	**7.0e-5**	**-0.43**	**4.7e-6**	**-0.50**	**9.9e-9**	**1.36**	0.768	0.19	0.995	0.04	0.454	0.26	**0.025**	**0.44**	**0.002**	**0.41**

Table S9. Properties of LFP beta coherence. (a) Descriptive statistics (b) Results of Kruskal-Wallis test (c) Post-hoc comparison results. p - p value, result of Tukey post-hoc test. g – effect size estimated by Hedge's g. Results are presented in Fig. 4. Source data are provided as a Source Data file. LFP: local field potential, AUC: area under the curve, dlPFC dorsolateral prefrontal cortex, GPe globus pallidis pars externa, Sal: saline, Amp: amphetamine, Apo: apomorphine, Hal: haloperidol,

a

		dlPFC-dlPFC					GPe-GPe					dlPFC-GPe				
		Sal	Amp	Apo1	Apo2	Hal	Sal	Amp	Apo1	Apo2	Hal	Sal	Amp	Apo1	Apo2	Hal
LFP coherence beta frequency	mean	15.781	17.846	15.881	15.212	14.033	15.519	17.975	16.559	15.537	13.852	14.971	17.044	15.22	14.226	13.382
	SD	1.602	1.408	2.354	1.8	1.892	1.444	0.908	1.985	1.346	0.725	1.812	1.48	2.072	1.283	1.211
LFP coherence beta AUC	mean	6.115	8.06	5.808	5.397	7.426	9.73	11.996	11.445	10.253	9.526	4.487	4.959	5.446	5.015	4.1
	SD	4.084	4.278	4.143	3.737	4.551	4.735	4.512	4.924	4.354	3.931	1.98	2.093	2.364	2.014	2.051
LFP coherence beta peak	mean	0.29	0.374	0.264	0.254	0.343	0.461	0.57	0.514	0.47	0.46	0.25	0.306	0.296	0.289	0.242
	SD	0.159	0.161	0.161	0.147	0.173	0.193	0.165	0.18	0.159	0.16	0.106	0.118	0.109	0.097	0.136

b

	dlPFC-dlPFC				GPe-GPe				dlPFC-GPe			
	χ^2	df	p	η^2	χ^2	df	P	η^2	t	df	p	g
LFP beta frequency	329.26	4	5.3e-70	0.268	414.20	4	2.4e-88	0.527	223.60	4	3.1e-47	0.407
LFP Beta AUC	60.30	4	2.5e-12	0.045	44.16	4	5.9e-9	0.069	32.86	4	1.3e-6	0.063
LFP beta peak	76.48	4	9.7e-16	0.063	52.31	4	1.2e-10	0.071	30.66	4	3.6e-6	0.051

c

		dlPFC-dlPFC						Gpe-GPe						dlPFC-GPe					
		beta freq.		beta AUC		Beta peak		beta freq.		beta AUC		Beta peak		beta freq.		beta AUC		Beta peak	
		p	g	p	g	p	g	p	g	p	g	p	g	p	g	p	g	p	g
Sal	Amp	9.9e-9	-1.35	5.1e-6	-0.47	2.1e-6	-0.52	9.9e-9	-1.95	7.8e-4	-0.48	5.3e-6	-0.60	9.9e-9	-1.27	0.339	-0.23	7.6e-4	-0.49
Sal	Apo1	0.007	-0.36	1.000	-0.03	0.512	0.10	9.9e-9	-1.17	1.9e-5	-0.58	2.4e-4	-0.51	2.9e-4	-0.64	1.7e-4	-0.62	0.014	-0.47
Sal	Apo2	0.019	0.33	0.214	0.18	0.082	0.24	0.996	-0.01	0.969	-0.11	1.000	-0.05	0.112	0.46	0.525	-0.26	0.131	-0.38
Sal	Hal	9.9e-9	1.01	0.010	-0.31	0.012	-0.32	9.9e-9	1.43	0.971	0.05	0.982	0.00	1.8e-6	1.00	0.609	0.19	0.942	0.07
Amp	Apo1	1.0e-8	0.58	5.5e-6	0.42	1.1e-8	0.59	0.152	0.26	0.987	-0.12	0.844	0.07	0.011	0.43	0.039	-0.41	0.998	0.02
Amp	Apo2	9.9e-9	1.59	1.0e-8	0.67	9.9e-9	0.78	9.9e-9	2.08	0.012	0.39	8.0e-6	0.61	9.9e-9	1.99	1.000	0.03	0.737	0.15
Amp	Hal	9.9e-9	2.27	0.441	0.14	0.316	0.18	9.9e-9	5.08	1.6e-4	0.59	1.4e-6	0.67	9.9e-9	2.63	0.012	0.41	1.1e-4	0.51
Apo1	Apo2	1.4e-8	0.59	0.257	0.20	0.881	0.12	9.9e-9	1.17	8.4e-4	0.49	3.1e-4	0.50	1.4e-8	1.12	0.066	0.38	0.929	0.13
Apo1	Hal	9.9e-9	1.07	0.009	-0.27	4.3e-5	-0.40	9.9e-9	2.43	3.5e-6	0.67	6.5e-5	0.56	9.9e-9	1/59	9.4e-7	0.76	0.002	0.47
Apo2	Hal	7.8e-8	0.64	2.7e-6	-0.49	4.5e-7	0.56	9.9e-9	1.57	0.752	0.17	0.995	0.06	0.047	0.67	0.040	0.45	0.030	0.40

Table S10. Properties of LFP beta PLV. (a) Descriptive statistics (b) Results of Kruskal-Wallis test (c) Post-hoc comparison results. p - p value, result of Tukey post-hoc test. g – effect size estimated by Hedge's g. Results are presented in Fig. S11. Source data are provided as a Source Data file.

freq: frequency, dlPFC dorsolateral prefrontal cortex, GPe: globus pallidis pars externa, Sal: saline, Amp: amphetamine, Apo: apomorphine, Hal: haloperidol, HF: high frequency, AUC: area under the curve

a

			Wide					narrow					HFD				
			Descriptive statistics					Descriptive statistics					Descriptive statistics				
			Sal	Amp	Apo1	Apo2	Hal	Sal	Amp	Apo1	Apo2	Hal	Sal	Amp	Apo1	Apo2	Hal
Ent. Probability			0.45	0.57	0.37	0.31	0.38	0.66	0.73	0.59	0.53	0.78	0.42	0.52	0.19	0.37	0.53
Vector length		mean	0.1	0.095	0.095	0.085	0.087	0.101	0.122	0.079	0.083	0.111	0.018	0.021	0.008	0.015	0.023
		SD	0.07	0.065	0.063	0.057	0.062	0.075	0.086	0.044	0.048	0.073	0.016	0.024	0.007	0.017	0.022
Preferred phase	Group by drugs	mean	-2.83	-2.82	-2.96	-2.83	-2.82	-2.11	-2.1	-2.47	-2.04	-2.2	2.24	3.13	1.43	1.09	-0.42
		ang. SD	0.65	0.63	0.6	0.86	0.87	1.06	0.74	0.64	0.85	1.27	1.37	1.17	1.03	1.34	1.35
			Low beta			High beta		Low beta			High beta		Low beta			High beta	
	Group by beta (Sal)	mean	-2.82			-2.82		-1.66			-2.19		0.58			2.68	
		ang. SD	0.62			0.68		1.11			1.04		1.31			1.31	
	Group by beta (all)	Mean	-2.81			-2.83		-1.94			-2.2		0.77			2.92	
		ang. SD	0.75			0.71		1.16			0.87		1.3			1.3	
	Group by LFP beta (all)	Mean	-2.73			-2.91		-1.65			-2.37		0.42			2.90	
		ang. SD	0.84			0.59		1.15			0.67		1.37			1.27	

b

				Cortical wide units			Cortical narrow units			Pallidal units		
		test	Test statistic	Statistic value	P value	Effect size	Statistic value	P value	Effect size	Statistic value	P value	Effect size
Entrainment probability		χ^2	χ^2	**51.87**	**1.5e-10**	**0.18**	**10.95**	**0.027**	0.19	**51.52**	**1.7e-10**	0.19
Vector length		KW	χ^2	**11.02**	**0.026**	**0.009**	8.12	0.087	0.04	**99.83**	**1.1e-20**	0.04
Preferred phase	Grouped by drugs	CM	P	9.32	0.0535		5.51	0.239		**33.45**	**9.7e-07**	
	Grouped by unit beta (Sal)	C,M	P	0.13	0.719		0.57	0.449		**3.88**	**0.049**	
	Grouped by unit beta (all)	CM	P	0.10	0.752		0.20	0.657		**11.91**	**6.0e-4**	
	Grouped by LFP beta (all)	CM	P	**8.48**	**0.004**		**13.87**	**0.0002**		**12.51**	**0.0004**	

c

		wide					narrow					HFD				
		Entrainment probability		Vector length		Preferred phase	Entrainment probability		Vector length		Preferred phase	Entrainment probability		Vector length		Preferred phase
		p	Φ	p	g	p	p	Φ	p	g	p	p	Φ	p	g	p
Sal	Amp	**0.0049**	**-0.12**	0.955	0.07		1.0000	-0.08				0.0749	-0.10	1.0000	-0.15	0.274
Sal	Apo1	1.0000	-0.06	0.996	0.08		1.0000	-0.06				**1.9e-4**	**-0.20**	**9.9e-9**	**0.68**	0.273
Sal	Apo2	**0.0017**	**-0.14**	0.087	0.23		1.0000	-0.13				1.0000	-0.05	**0.0047**	**0.16**	0.359
Sal	Hal	0.4696	0.07	0.054	0.20		1.0000	-0.13				**0.0452**	**0.11**	**0.0305**	**-0.25**	0.486
Amp	Apo1	**0.0018**	**-0.17**	1.000	0.01		1.0000	-0.13				**2.5e-8**	**-0.29**	**9.9e-9**	**0.60**	0.110
Amp	Apo2	**3.7e-10**	**-0.27**	0.401	0.17	--	0.1523	-0.21	--		--	**0.0017**	**-0.15**	**0.0049**	**0.27**	0.123
Amp	Hal	**2.0e-6**	**-0.19**	0.343	0.13		1.0000	0.06				1.0000	0.01	**0.0442**	**-0.07**	0.274
Apo1	Apo2	2.0971	0.06	0.640	0.17		1.0000	0.05				**0.0071**	**-0.17**	**2.0e-5**	**-0.48**	0.273
Apo1	Hal	1.0000	-0.01	0.623	0.12		0.8143	-0.19				**1.5e-8**	**0.31**	**9.9e-9**	**-0.76**	0.597
Apo2	Hal	0.5619	-0.07	1.000	-0.04		**0.0320**	**-0.26**				**0.0010**	**0.16**	**2.5e-8**	**-0.37**	0.538

Table S11. Properties of SUA-LFP beta entrainment. (a) Descriptive statistics (b) Results of statistical tests. χ2: Chi-square test. KW: Kruskal-Wallis test. CM: circular median test. For each test the appropriate effect size estimator was selected. χ2: Cramer's v. KW: η^2. For CM test there is no defined effect size estimator for the best of our knowledge (c) Post-hoc comparison results. p - p value, result of Tukey post-hoc test. g – effect size estimated by Hedge's g. Φ - effect size estimated by phi coefficient. Comparisons that did not reach statistical significance and didn't require post-hoc test are marked with --. Results are presented in Fig. 5. Source data are provided as a Source Data file. SD: standard deviation, Sal: saline, Amp: amphetamine, Apo: apomorphine, Hal: haloperidol, HF: high frequency,

a

		dlPFC HF amplitude to GPe beta phase					GPe HF amplitude to dlPFC beta phase				
		Sal	Amp	Apo1	Apo2	Hal	Sal	Amp	Apo1	Apo2	Hal
Max PAC beta f	mean	15.41	17.35	17.64	15.27	12.93	15.525	14.80	16.08	15.23	15.03
	SD	3.62	4.72	4.21	4.15	3.26	5.03	4.96	5.41	5.20	4.62
Max PAC	mean	6.701	5.649	4.960	4.957	8.009	3.055	3.926	2.895	2.978	3.420
	SD	3.803	3.202	2.874	2.727	5.138	1.160	1.785	0.818	1.019	1.724

b

		F	df1	df2	p	η^2
dlPFC HF – GPe beta	Max PAC frequency	**17.71**	**4**	**454**	**1.6e-13**	**0.135**
	Max PAC	**10.12**	**4**	**454**	**7.5e-8**	**0.082**
GPe HF – dlPFC beta	Max PAC frequency	**0.88**	**4**	**452**	**0.48**	**0.008**
	Max PAC	**10.07**	**4**	**452**	**8.2-8**	**0.082**

c

		dlPFC HF amplitude to GPe beta phase				GPe HF amplitude to dlPFC beta phase			
		Beta frequency of maximum PAC		Maximum PAC		Beta frequency of maximum PAC		Maximum PAC	
		p	g	p	g	p	g	p	g
Sal	Amp	**0.0032**	**-0.45**	0.1727	0.30	---		**2.6e-5**	**-0.56**
Sal	Apo1	**0.0028**	**-0.57**	**0.0112**	**0.51**			0.9439	0.16
Sal	Apo2	0.9995	0.03	**0.0115**	**0.51**			0.9963	0.07
Sal	Hal	**9.3e-4**	**0.71**	0.1276	-0.30			0.4496	-0.26
Amp	Apo1	0.9875	-0.06	0.6629	0.22			**3.2e-6**	**0.68**
Amp	Apo2	**0.0039**	**0.46**	0.6630	0.23			**2.2e-5**	**0.61**
Amp	Hal	**9.9e-9**	**1.03**	**7.6e-5**	**-0.59**			0.1045	0.29
Apo1	Apo2	**0.0030**	**0.56**	1.0000	0.00			0.9960	-0.09
Apo1	Hal	**9.9e-9**	**1.24**	**2.2e-6**	**-0.74**			0.1574	-0.39
Apo2	Hal	**0.0048**	**0.62**	**2.3e-6**	**-0.75**			0.3097	-0.31

Supplementary Table 12. Properties of LFP HF beta PAC. (a) Descriptive statistics. (b) Results of one-way anova tests with PAC properties as dependent factors and drug condition as independent factor. Test was conducted for each PAC property (i.e. each row) separately. (c) Post-hoc Tukey test results. p-p value, results of Tukey post-hoc test. g-effect size estimated by Hedge's g. Comparisons that did not reach statistical significance and didn't require post-hoc test are marked with ---. Results are presented in Figure 6. Source data are provided as a Source Data file. PAC: phase amplitude coupling, f: frequency, SD: standard deviation, dlPFC dorsolateral prefrontal cortex, GPe globus pallidis pars externa, Sal: saline, Amp: amphetamine, Apo: apomorphine, Hal: haloperidol, HF: high frequency.

	Dependent variable	Factor	F	DF 1	DF 2	p
Beta nPSD	High-beta frequency	**Time**	**33.949**	**1**	**462**	**1.1e-08**
		DRT	2.1604	1	462	0.1423
		**Time x DRT**	**25.36**	**1**	**462**	**6.8e-07**
	Low-beta frequency	Time	0.101	1	414	0.7508
		DRT	3.736	1	414	0.0539
		Time x DRT	1.1366	1	414	0.287
	High-beta AUC	Time	0.7346	1	736	0.3917
		DRT	3.3476	1	736	0.0677
		Time x DRT	3.558	1	736	0.0597
	Low-beta AUC	Time	1.4921	1	665	0.2223
		**DRT**	**40.934**	**1**	**665**	**2.9e-10**
		**Time x DRT**	**6.3891**	**1**	**665**	**0.0117**
Beta coherence	High-beta frequency	**Time**	**8.0614**	**1**	**120**	**0.0053**
		DRT	3.266	1	120	0.0732
		Time x DRT	0.4526	1	120	0.5024
	Low-beta frequency	Time	0.5466	1	201	0.4606
		DRT	0.6182	1	201	0.4327
		Time x DRT	2.0826	1	201	0.1505
	High-beta AUC	Time	1.9532	1	200	0.1638
		DRT	2.5503	1	200	0.11185
		**Time x DRT**	**5.6426**	**1**	**200**	**0.0185**
	Low-beta AUC	Time	0.0221	1	476	0.88185
		DRT	0.2927	1	476	0.5887
		Time x DRT	3.7752	1	476	0.0526

Supplementary Table 13: Time and DRT effects on beta properties in PD patients. To estimate the effect of time and DRT on beta properties in patients with PD, a mixed linear effect model (MLEM) was constructed for each dependent variable (columns 1 and 2). The model included fixed effect terms for time, DRT and their interaction (column 3). The resulted estimated coefficients are presented in column 4. One-way ANOVA was used on the model output to determine the significance of each factor. ANOVA results are presented in columns 4-7. Note that a separate model was constructed for high-beta and low-beta properties. Subjects were clustered as having low-beta, high-beta or both and traces were included in the analysis accordingly. i.e. If a subject had low-beta, all his traces were included in the low-beta analysis and the same for high-beta. Only traces with significant beta peaks were included in the frequency models. Time x DRT estimated-coefficient represents time effect given on DRT condition, in addition to the main time effect. A significant positive estimated-coefficient indicated that time slope in the on DRT condition was significantly more positive (or less negative) than time slope in the off DRT condition, and vice versa for negative values. Source data are provided as a Source Data file. nPSD: normalized power spectral density, DF: degrees of freedom, AUC: area under the curve, DRT: dopamine replacement therapy

Pts.	Age	Gender	Duration of disease (years)	Baseline Medications (dose)	Levodopa Equivalent Dose (LED)	Baseline UPDRS III motor score (PD)	DBS Lead Target [x,y,z]	Optimal stimulation parameters: (Frequency (Hz); Pulse Duration (μs); Contact configuration; Voltage(V))
Jur 01	60-70	F	8-10	Stalevo 50 mg q4d<br>Rasagiline 2 mg qid	1066	35	Left:<br>[-12, -4, -4.5]<br>Right:<br>[12.25, -3.5, -5.5]	Left:<br>(180; 60; c+9-; 2)<br>Right:<br>(180; 60; c+1-; 1.9)
Jur 03	50-60	M	8-10	Carbidopa 12.5 mg q3h<br>Levodopa 125 mg q3h<br>Biperiden 1mg q4h<br>Ropinirole 4mg qid	1257.5	31	Left:<br>[-11.25, -1.75, -4.5]<br>Right:<br>[11, -2.5, -5]	Left:<br>(130; 60; c+9-; 2.1)<br>Right:<br>(130; 60; c+1-; 1.6)
Jur 05	50-60	F	8-10	Carbidopa 25 mg q6h<br>Levodopa 250 mg q6h	1125	42	Left:<br>[-10.5, -2.5, -4]<br>Right:<br>[10.75, -3.5, -5]	Left:<br>(130; 60; c+8-11-; 1.9)<br>Right:<br>(130; 60; c+1-2-; 1.3)
Jur 06	50-60	F	8-10	Carbidopa 25 mg q5h<br>Levodopa 250 mg q5h<br>Rasagiline 1mg qid<br>Ropinirole 8mg qid	2165	43	Left:<br>[-11, -3, -4]<br>Right:<br>[11.5, -3, -4.5]	Left:<br>(130; 60; c+9-11-; 2.2)<br>Right:<br>(130; 60; c+1-3-; 1.8)

Supplementary Table 14: Patient demographics and treatment.
