# Supplementary material for: Modulation of dopamine tone induces frequency shifts in cortico-basal ganglia beta oscillations
Source: Nat Commun. 2021 Dec 2;12:7026. doi: 10.1038/s41467-021-27375-5 (PMC8640051; doi:10.1038/s41467-021-27375-5)
Supplement: Supplementary file 2 — Description of Additional Supplementary Files [file 41467_2021_27375_MOESM2_ESM.pdf]

Description of Additional Supplementary Files

File Name: Supplementary Movie 1
Description: Eye movement during control recordings.

File Name: Supplementary Movie 2
Description: Eye movement during amphetamine recordings.

File Name: Supplementary Movie 3
Description: Eye movement during apomorphine recordings.

File Name: Supplementary Movie 4
Description: Eye movement during haloperidol recordings.
