# Supplementary material for: Modulation of dopamine tone induces frequency shifts in cortico-basal ganglia beta oscillations
Source: Nat Commun. 2021 Dec 2;12:7026. doi: 10.1038/s41467-021-27375-5 (PMC8640051; doi:10.1038/s41467-021-27375-5)
Supplement: Supplementary file 7 — Reporting Summary [file 41467_2021_27375_MOESM7_ESM.pdf]

nature research

Corresponding author(s): Liliya Iskhakova
Last updated by author(s): Sep 22, 2021

Reporting Summary

Nature Research wishes to improve the reproducibility of the work that we publish. This form provides structure for consistency and transparency in reporting. For further information on Nature Research policies, see our Editorial Policies and the Editorial Policy Checklist.

Statistics

For all statistical analyses, confirm that the following items are present in the figure legend, table legend, main text, or Methods section.

n/a	Confirmed	
☐	☒	The exact sample size (n) for each experimental group/condition, given as a discrete number and unit of measurement
☐	☒	A statement on whether measurements were taken from distinct samples or whether the same sample was measured repeatedly
☐	☒	The statistical test(s) used AND whether they are one- or two-sided<br>*Only common tests should be described solely by name; describe more complex techniques in the Methods section.*
☐	☒	A description of all covariates tested
☐	☒	A description of any assumptions or corrections, such as tests of normality and adjustment for multiple comparisons
☐	☒	A full description of the statistical parameters including central tendency (e.g. means) or other basic estimates (e.g. regression coefficient) AND variation (e.g. standard deviation) or associated estimates of uncertainty (e.g. confidence intervals)
☐	☒	For null hypothesis testing, the test statistic (e.g. F, t, r) with confidence intervals, effect sizes, degrees of freedom and P value noted<br>*Give P values as exact values whenever suitable.*
☒	☐	For Bayesian analysis, information on the choice of priors and Markov chain Monte Carlo settings
☐	☒	For hierarchical and complex designs, identification of the appropriate level for tests and full reporting of outcomes
☐	☒	Estimates of effect sizes (e.g. Cohen's d, Pearson's r), indicating how they were calculated

Our web collection on statistics for biologists contains articles on many of the points above.

Software and code

Policy information about availability of computer code

Data collection	AlphaLab SnR Stimulation and Recording System, AlphaOmega Engineering, Israel
Data analysis	MATLAB R2018B<br>In addition to matlab's built-in functions we used matlab circ_stat toolbox (P. Berens, CircStat: A Matlab Toolbox for Circular Statistics, Journal of Statistical Software, Volume 31, Issue 10, 2009) for circular statistics

For manuscripts utilizing custom algorithms or software that are central to the research but not yet described in published literature, software must be made available to editors and reviewers. We strongly encourage code deposition in a community repository (e.g. GitHub). See the Nature Research guidelines for submitting code & software for further information.

Data

Policy information about availability of data

All manuscripts must include a data availability statement. This statement should provide the following information, where applicable:

- Accession codes, unique identifiers, or web links for publicly available datasets
- A list of figures that have associated raw data
- A description of any restrictions on data availability

Data analyses were performed with custom written scripts in MATLAB (Mathworks, Natick, MA). Codes used for analysis are available here:https://doi.org/10.6084/m9.figshare.16660819. A minimal dataset is available here: https://doi.org/10.6084/m9.figshare.16660858. Source data for all relevant figures is uploaded with manuscript.

Field-specific reporting

Please select the one below that is the best fit for your research. If you are not sure, read the appropriate sections before making your selection.

☒ Life sciences ☐ Behavioural & social sciences ☐ Ecological, evolutionary & environmental sciences

For a reference copy of the document with all sections, see nature.com/documents/nr-reporting-summary-flat.pdf

Life sciences study design

All studies must disclose on these points even when the disclosure is negative.

Sample size

sample size is describes in tables S1 and S2 in the supplementary information

NHP study:
number of animals: 4 - 2 in acute experiments, 2 in chronic experiments

number of cortical LFP sites per condition:
sal: 141, amp: 117, apo1: 124, apo2: 125, hal: 104
number of pallidal LFP sites per condition:
sal: 126, amp: 93, apo1: 95, apo2: 94, hal: 91

number of cortical wide units per condition:
sal: 496, amp: 375, apo1: 128, apo2: 304, hal: 412
number of cortical narrow units per condition:
sal: 88, amp: 74, apo1: 25, apo2: 69, hal: 62
number of pallidal units per condition:
sal: 439, amp: 383, apo1: 122, apo2: 322, hal: 361

number of subthalamic units:
naive: 128
MPTP: 102

PD patients study:
number of patients: 4

number of recording sites per patient: 12 bipolar contacts (6 per hemisphere), one patient had only 1 malfunctioning contact and therefore had only 3 bipolar contacts in one hemisphere

number of recording sessions per patient (on/off dopamine replacement therapy):
jur 01: on: 13, off: 19
jur 03: on: 10, off: 6
jur 05: on: 9, off: 4
jur 06: on: 11, off: 19

Data exclusions

NHP study:
Units with spike width larger than 3 SD over the mean were excluded from further analysis. This units didn't have typical spike shape and probably represented noise.
LFP sites and single units with beta area under curve (AUC) larger than 5 standard deviations above the mean were defined as outliers and excluded from further analysis.

PD patients study:
Data from the first week after the surgery was excluded to avoid insertion effect.
Data recorded more than 250 days after the surgery was excluded from analysis. Only one patient had data recorded from this time period so we excluded it to avoid bias

Replication

no replication was made

Randomization

injection order was pseudo-randomized for each animal

Blinding

the study didn't include blind design

Reporting for specific materials, systems and methods

We require information from authors about some types of materials, experimental systems and methods used in many studies. Here, indicate whether each material, system or method listed is relevant to your study. If you are not sure if a list item applies to your research, read the appropriate section before selecting a response.

April 2020

Materials & experimental systems

n/a	Involved in the study
☒	☐ Antibodies
☒	☐ Eukaryotic cell lines
☒	☐ Palaeontology and archaeology
☐	☒ Animals and other organisms
☐	☒ Human research participants
☐	☒ Clinical data
☒	☐ Dual use research of concern

Methods

n/a	Involved in the study
☒	☐ ChIP-seq
☒	☐ Flow cytometry
☐	☒ MRI-based neuroimaging

Animals and other organisms

Policy information about studies involving animals; ARRIVE guidelines recommended for reporting animal research

Laboratory animals	four healthy, young-adult, female vervet monkeys (Chlorocebus aethiops sabaeus), The age of monkeys at the time of the experiment was 5-8 years (G: 7-8, D: 5-6, K: 6-7, S: 6-7).
Wild animals	n/a
Field-collected samples	n/a
Ethics oversight	All experimental protocols were conducted in accordance with the National Institutes of Health Guide for the Care and Use of Laboratory Animals (National Research Council, 2011) and with the Hebrew University guidelines for the use and care of laboratory animals in research. The experiments were supervised by the Institutional Animal Care and Use Committee of the Faculty of Medicine, the Hebrew University. The Hebrew University is an Association for Assessment and Accreditation of Laboratory Animal Care internationally accredited institute.

Note that full information on the approval of the study protocol must also be provided in the manuscript.

Human research participants

Policy information about studies involving human research participants

Population characteristics	Patient demographic is described in table S9 in the supplementary information. Cohort included 3 female and 1 male, age 52-66, with disease duration of 8-10 years.<br>Patients had (i) advanced idiopathic PD; (ii) long-term levodopa use with reduced efficacy, on-off motor fluctuations and increased incidence of medication-induced side effects; (iii) normal cognitive function or mild-moderate cognitive decline as defined by Addenbrooke's cognitive examination (ACE) >75 and frontal assessment battery (FAB) >10.
Recruitment	Patients who met the inclusion criteria described above were suggested to participate in the study by their physicians and signed an informed consent form.
Ethics oversight	The study was authorized and supervised by the IRB of Hadassah Medical Center (no. 0403-13-HMO) and the Israel Ministry of Health (no. HT6752).

Note that full information on the approval of the study protocol must also be provided in the manuscript.

Clinical data

Policy information about clinical studies

All manuscripts should comply with the ICMJE guidelines for publication of clinical research and a completed CONSORT checklist must be included with all submissions.

Clinical trial registration	Clinical Trials Registration number: NCT01962194.
Study protocol	NCT01590056
Data collection	Patients underwent recordings during 170-400 post-operative days. Recordings from the first week post-surgery were excluded from the dataset to avoid insertion effect. Only recordings from the first 250 days were included in the analysis because recordings after 250th day all came from a single patient (jur01).<br>Post-operative recordings were acquired in an outpatient setting. Patients had clinical evaluations and recording sessions every 2–4 . During recordings, patients were instructed to sit quietly for the rest-state session, which lasted three minutes. In addition, sessions included recordings during performance of four tasks, which are out of the current paper scope.<br>Recordings took place during an off-medication and on-medication states. Off-medication recordings took place after overnight withdrawal of DRT. On-medication recordings took place after confirmation of a substantial improvement in the parkinsonian motor clinical symptoms by the patient and the examiner.
Outcomes	No clinical measures were included in current study.<br>Patient STN LFP activity was recorded from all the bipolar contact pair combinations in both hemispheres through the Medtronic PC

+S recording setting.

Magnetic resonance imaging

Experimental design

Design type	resting state
Design specifications	N/A
Behavioral performance measures	N/A

Acquisition

Imaging type(s)	Structural, MRI scan was performed on NHPs post surgery to assist in trajectory selection. No analysis of MRI data was performed.
Field strength	3T Skyra Siemens MRI
Sequence & imaging parameters	Coronal T2 sequence: TR = 5510ms, TE = 66 ms, slice thickness = 1 mm, 6 averages,FA = 180 degrees, FOV = 240*240, matrix = 320*320, (0.75*0.75)
Area of acquisition	Whole brain scan was used

Diffusion MRI ☐ Used ☒ Not used

Preprocessing

Preprocessing software	N/A
Normalization	N/A
Normalization template	N/A
Noise and artifact removal	N/A
Volume censoring	N/A

Statistical modeling & inference

Model type and settings	N/A
Effect(s) tested	N/A

Specify type of analysis: ☐ Whole brain ☐ ROI-based ☐ Both

Statistic type for inference (See Eklund et al. 2016)	N/A
Correction	N/A

Models & analysis

n/a	Involved in the study
☒	☐ Functional and/or effective connectivity
☒	☐ Graph analysis
☒	☐ Multivariate modeling or predictive analysis
